# Global burden of pancreatitis among individuals aged 15–39 years: a systematic analysis from the 2021 GBD study

**DOI:** 10.3389/fmed.2025.1572346

**Published:** 2025-05-27

**Authors:** Biwu Xu, Kaiyuan Li, Peng Liu

**Affiliations:** ^1^Department of Hepatobiliary Surgery, Taizhou Hospital of Zhejiang Province Affiliated to Wenzhou Medical University, Taizhou, China; ^2^Graduate School of Dalian Medical University, Dalian Medical University, Dalian, China; ^3^Jiangxi Medical College, Nanchang University, Nanchang, China

**Keywords:** pancreatitis, youth, Global Burden of Disease, socio-demographic index, epidemiological trends

## Abstract

**Background:**

Pancreatitis represents a significant global public health challenge, yet there is a lack of comprehensive analyses focusing on the burden of pancreatitis and its long-term trends among young individuals aged 15–39 years.

**Methods:**

This study utilized data from the Global Burden of Disease (GBD 2021) database to analyze the prevalence, incidence, and disability-adjusted life years (DALYs) associated with pancreatitis in the 15–39 age group from 1990 to 2021. Temporal trends in disease burden were assessed by calculating the estimated annual percentage change (EAPC), with point estimates and their 95% uncertainty intervals (UIs) reported.

**Results:**

Between 1990 and 2021, the global number of cases related to pancreatitis—including prevalence, incidence, DALYs, and deaths—substantially increased in the 15–39 age group. However, age-standardized rates for prevalence, incidence, DALYs, and mortality showed a declining trend. Gender-specific analysis revealed that females had lower prevalence and incidence rates compared to males. Socio-demographic Index (SDI)-based subgroup analysis indicated that low-SDI regions experienced the largest increases in DALYs and deaths, while high-SDI regions showed the most significant declines in age-standardized DALYs and mortality rates. Geographically, East Asia demonstrated the largest decrease in the burden of pancreatitis, whereas Western Sub-Saharan Africa exhibited the highest increases in case numbers and deaths. Age-stratified analysis showed that individuals aged 35–39 years had the greatest increases in case numbers and disease burden, despite experiencing the most notable decline in incidence rates. Conversely, the 15–19 age group exhibited reductions in disease burden and mortality rates.

**Conclusion:**

This study highlights that, while the global number of pancreatitis cases among young individuals aged 15–39 has risen from 1990 to 2021, the overall disease burden has declined, particularly in high-income regions. In contrast, the disease burden in low-income regions continues to rise.

## 1 Introduction

Pancreatitis is a severe gastrointestinal disease characterized by inflammatory damage to the pancreas, which can lead to multiple health complications, including organ failure, chronic pain, and significantly reduced quality of life (1).

Recent epidemiological studies have shown a rising trend in the incidence of pancreatitis among adolescents and young adults. For example, in the United States, hospitalizations due to acute pancreatitis in individuals aged 18–30 years increased by approximately 25% between 2002 and 2015 (2). Similar upward trends have been observed in Europe and parts of Asia, particularly among populations with high rates of alcohol consumption and obesity (3, 4). These findings suggest that pancreatitis is no longer confined to older populations and has become an emerging health challenge among younger age groups. Such evidence reinforces the urgency of monitoring the disease burden in this population segment and informing age-specific preventive strategies (5, 6). Despite growing recognition of the clinical significance of pancreatitis in this age group, epidemiological studies focusing on its burden and trends at the global, regional, and national levels remain limited (7, 8).

The Global Burden of Disease (GBD) study provides a comprehensive platform for analyzing the burden of various diseases across regions, countries, and populations (9, 10). By estimating prevalence, incidence, mortality, and disability-adjusted life years (DALYs), GBD data offers a unique perspective on the epidemiological patterns of pancreatitis.

This study leverages data from the GBD 2021 database to quantify the global burden of pancreatitis in individuals aged 15–39 years from 1990 to 2021, focusing on long-term trends in incidence, mortality, and DALYs. Additionally, this analysis incorporates stratifications by gender, region, and Socio-demographic index (SDI) to identify high-burden populations and regions. The findings aim to provide evidence-based guidance for allocating global health resources and shaping policies. Moreover, the results are expected to inform future public health interventions, clinical management, and epidemiological research on pancreatitis, ultimately contributing to reducing the burden of this global health challenge.

## 2 Methods

### 2.1 Data source and disease definition

This study analyzed pancreatitis data for individuals aged 15–39 years from the Global Burden of Disease Study 2021 (GBD 2021) (11). GBD 2021 provides the latest estimates of the epidemiological burden of more than 369 diseases and injuries across 21 GBD regions and 204 countries and territories from 1990 to 2021 (12). All data are freely accessible through the Global Health Data Exchange^1^, with detailed information on data, methods, and statistical models available in previous reports (13).

In the GBD 2021 database, pancreatitis is defined as a severe gastrointestinal disease characterized by pancreatic inflammation, which can be triggered by various factors and is commonly associated with symptoms such as abdominal pain, indigestion, and organ failure. Pancreatitis can result in severe health consequences, including acute kidney failure, respiratory failure, SIRS, and death. Due to its complex etiology and potential for recurrent episodes, pancreatitis has emerged as a critical global public health concern. According to the International Classification of Diseases (ICD), pancreatitis is coded as 577.0 (acute pancreatitis) and 577.1 (chronic pancreatitis) in ICD-9, and as K85 (acute pancreatitis) and K86 (chronic pancreatitis) in ICD-10. These codes encompass both acute and chronic forms of pancreatitis, reflecting the disease’s diverse clinical manifestations and its significant health impact worldwide.

### 2.2 SDI

The SDI is a composite indicator introduced in 2015 by the Institute for Health Metrics and Evaluation to assess a country or region’s level of socioeconomic development and its correlation with population health outcomes. Based on the SDI scores, the 204 countries and regions in the GBD 2021 study were categorized into five SDI groups: low, medium-low, medium, medium-high, and high.

### 2.3 DALYs

DALYs were calculated as the sum of years of life lost (YLLs) due to premature mortality and years lived with disability (YLDs), in accordance with the standard methods employed in the GBD 2021 study (11). Detailed methodology is available in previously published reports (14).

### 2.4 Estimated annual percentage change and percentage change (EAPC)

Temporal trends in prevalence, incidence, DALYs, and mortality were assessed using the EAPC, based on a log-linear regression model as outlined in prior GBD methodological literature (15). The calculation of EAPC values and 95% confidence intervals were calculated to determine the direction and significance of change over time (16).

### 2.5 Death rate calculation

The “deaths” indicator in this study was measured mainly by the death rate. The formula for calculating the death rate is as follows: Death Rate (per 100,000 population) = number of deaths from pancreatitis/total population in the 15–39 age group x 100,000. Data were stratified by country, region and time period.

### 2.6 Data analysis

All analyses were conducted using R software (version 4.3.1), with visualizations produced using the ggplot2 package. Final figure editing was performed using Adobe Illustrator (CS5 version).

## 3 Results

### 3.1 Global level

**Figure 1:**
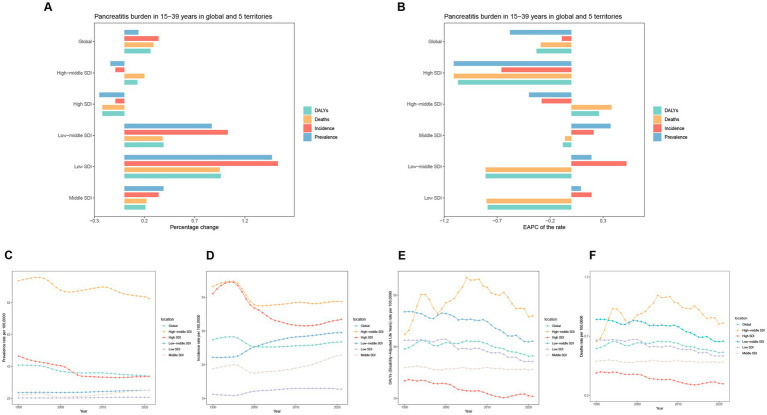
Temporal trend of pancreatitis burden in 15–39 years in global and 5 territories. **(A)** Percentage change in cases of prevalence, incidence, DALYs, and Deaths in 1990 and 2021. **(B)** The EAPC of prevalence, incidence, and DALY rates from 1990 to 2021. **(C–F)** The rates of prevalence, incidence, DALYs, and Deaths in global and 5 territories.

Globally, the number of pancreatitis cases—including prevalence, incidence, DALYs, and deaths among individuals aged 15–39 years increased significantly from 1990 to 2021. Specifically, the number of prevalent cases rose from 940.26 thousand in 1990 to 1,069.42 thousand in 2021, an increase of 42.9%. Incident cases increased from 607.83 thousand in 1990 to 814.5 thousand in 2021, reflecting a growth of 27.73%. DALYs increased from 840 thousand in 1990 to 1.06 million in 2021, an increase of 26%. Similarly, deaths rose from 13.07 thousand in 1990 to 16.8 thousand in 2021, a 29% increase (Figure 1A; Table 1; Supplementary Tables 1–3).

**Table 1:** The prevalence of pancreatitis burden in people aged 15–39 years in global and 5 cases and rates, and the trends from 1990 to 2021.

location	Prevalent cases			Prevalent rates		
	1990 thousand (95%UI)	2021 thousand (95%UI)	percentage Change (100%)	1990 per (95%UI)	2021 per (95%UI)	EAPC (95% CI)
Global	940.26 (602.13–1374.39)	1069.42 (688.08–1574.63)	0.14	42.9 (27.47–62.71)	35.95 (23.13–52.93)	−0.58 (−0.65–−0.51)
High-middle SDI	433.03 (275.71–650.85)	372.38 (240.15–558.54)	−0.14	95.69 (60.93–143.82)	84.58 (54.55–126.86)	−0.4 (−0.48–−0.32)
High SDI	168.29 (104.65–253.52)	126.35 (83.78–181.24)	−0.25	48.5 (30.16–73.07)	35.77 (23.72–51.31)	−1.11 (−1.29–−0.93)
Low-middle SDI	116.09 (71.93–176.25)	216.96 (134.26–333.99)	0.87	25.6 (15.86–38.87)	27.04 (16.73–41.62)	0.19 (0.17–0.22)
Low SDI	41.13 (24.6–63.4)	101.61 (60.79–157.74)	1.47	22.31 (13.35–34.4)	22.63 (13.54–35.13)	0.09 (0.07–0.1)
Middle SDI	180.91 (115.06–272.06)	251.4 (159.93–375.83)	0.39	24.04 (15.29–36.15)	27.11 (17.24–40.52)	0.37 (0.22–0.53)
Andean Latin America	2.31 (1.52–3.37)	4.12 (2.67–6.18)	0.78	14.94 (9.83–21.77)	15.22 (9.87–22.83)	0.1 (0.05–0.14)
Australasia	2.15 (1.31–3.28)	2.7 (1.61–4.19)	0.26	26.31 (16.08–40.25)	25.74 (15.4–40)	−0.06 (−0.09–−0.04)
Caribbean	3.82 (2.25–6.09)	5.08 (2.98–8.26)	0.33	25.69 (15.15–40.98)	27.89 (16.4–45.35)	0.14 (0.08–0.2)
Central Asia	31.58 (19.15–46.65)	46.26 (27.53–68.58)	0.46	110.97 (67.31–163.96)	123.73 (73.64–183.43)	0.21 (0.14–0.27)
Central Europe	32.98 (19.64–51.17)	18.18 (12.08–26.33)	−0.45	70.4 (41.92–109.23)	51.9 (34.48–75.17)	−0.87 (−1.19–−0.54)
Central Latin America	14.81 (9.72–21.12)	22.45 (15.54–31.78)	0.52	21.7 (14.23–30.94)	22.19 (15.36–31.41)	0.08 (0.02–0.15)
Central Sub-Saharan Africa	3.66 (2.11–5.64)	9.61 (5.44–14.62)	1.63	17.61 (10.17–27.16)	17.76 (10.06–27.03)	0.14 (0.1–0.18)
East Asia	99.71 (59.35–157.82)	50.44 (31.4–78.4)	−0.49	17.63 (10.49–27.9)	10.53 (6.55–16.37)	−2.04 (−2.71–−1.37)
Eastern Europe	348.12 (214.03–528.08)	300.54 (184.92–455.67)	−0.14	405.88 (249.54–615.71)	454.17 (279.45–688.6)	0.32 (0.22–0.42)
Eastern Sub-Saharan Africa	14.86 (8.78–22.95)	37.9 (22.52–59.08)	1.55	20.97 (12.39–32.37)	21.64 (12.85–33.73)	0.14 (0.11–0.17)
High-income Asia Pacific	45.62 (26.83–69.11)	23.8 (15.15–34.28)	−0.48	67.59 (39.75–102.39)	47.1 (29.99–67.83)	−0.56 (−0.84–−0.27)
High-income North America	61.86 (36.47–95.79)	46.36 (30.97–67.08)	−0.25	54.59 (32.18–84.53)	37.64 (25.14–54.46)	−1.62 (−1.92–−1.32)
North Africa and Middle East	45.81 (27.89–71.71)	92.04 (55.49–141.66)	1.01	34.23 (20.84–53.58)	36.2 (21.82–55.71)	0.25 (0.22–0.28)
Oceania	0.39 (0.23–0.62)	0.84 (0.49–1.33)	1.15	14.69 (8.52–23.51)	14.87 (8.71–23.54)	0.05 (0.03–0.07)
South Asia	115.53 (69.61–179.2)	226.38 (138.44–350.61)	0.96	26.77 (16.13–41.52)	28.62 (17.5–44.33)	0.24 (0.22–0.27)
Southeast Asia	38.9 (23.65–61.17)	63.09 (37.86–100.94)	0.62	19.74 (12–31.05)	22.75 (13.65–36.4)	0.47 (0.45–0.49)
Southern Latin America	5.45 (3.38–8.15)	7.24 (5.19–9.72)	0.33	28.55 (17.69–42.72)	28.08 (20.11–37.69)	−0.07 (−0.13–0)
Southern Sub-Saharan Africa	6.42 (3.91–9.9)	10.36 (6.18–15.89)	0.61	29.71 (18.07–45.82)	30.44 (18.17–46.67)	0.01 (−0.07–0.09)
Tropical Latin America	16.72 (12.18–22.62)	26.33 (18.61–36.34)	0.57	26 (18.94–35.17)	29.81 (21.07–41.15)	0.51 (0.38–0.63)
Western Europe	33.32 (21.38–48.58)	31 (21.32–43.41)	−0.07	23.12 (14.84–33.71)	23.89 (16.43–33.45)	−0.22 (−0.34–−0.1)
Western Sub-Saharan Africa	16.24 (9.62–25.11)	44.7 (26.7–68.47)	1.75	22.69 (13.44–35.09)	23.38 (13.96–35.81)	0.12 (0.09–0.14)

Despite the notable increase in case numbers, age-standardized rates of prevalence, incidence, DALYs, and mortality all showed a declining trend. Based on the EAPC, the prevalence, incidence, DALYs, and mortality rates declined at EAPCs of −0.58 (95% CI: −0.65 to −0.51), −0.09 (95% CI: −0.16 to −0.03), −0.33 (95% CI: −0.47 to −0.02), and −0.29 (95% CI: −0.44 to −0.15), respectively (Figure 1B; Table 1; Supplementary Tables 1–3).

Sex-specific analysis revealed that females had higher numbers of prevalent and incident cases compared to males, but lower numbers of DALYs and deaths. Further evaluation indicated that females had lower age-standardized prevalence and incidence rates, but higher DALYs and mortality rates than males (Table 2). These gender differences may reflect greater disease progression and severity risks among males, although the underlying mechanisms warrant further investigation.

**Table 2:** Global prevalence, incidence, DALYs, and death rates and trends of pancreatitis in men and women aged 15–39 years, 1990 to 2021.

Sex	Cases			Rates		
	1990 thousand (95%UI)	2021 thousand (95%UI)	Percentage Change (100%)	1990 per (95%UI)	2021 per (95%UI)	EAPC (95% CI)
Female (Prevalence)	347.61 (219.2–516.49)	411.37 (262.67–614.38)	0.18 (0.2–0.19)	32.09 (20.23–47.68)	28.08 (17.93–41.93)	−0.45
Male (Prevalence)	592.65 (381.19–885.42)	658.04 (426.47–964.59)	0.11 (0.12–0.09)	53.47 (34.39–79.88)	43.59 (28.25–63.89)	−0.66
Female (Incidence)	238.3 (171.65–314.65)	323.7 (240.57–420.1)	0.36 (0.4–0.34)	22 (15.84–29.04)	22.09 (16.42–28.67)	−0.03
Male (Incidence)	369.53 (266.79–485.21)	490.8 (363.23–637.71)	0.33 (0.36–0.31)	33.34 (24.07–43.77)	32.51 (24.06–42.24)	−0.14
Female (DALYs)	0.21 (0.16–0.27)	0.23 (0.19–0.29)	0.1 (0.19–0.07)	19 (14.7–24.89)	15.88 (12.63–19.9)	−0.6
Male (DALYs)	0.63 (0.53–0.78)	0.82 (0.7–0.98)	0.3 (0.32–0.26)	56.89 (47.97–70.39)	54.57 (46.45–65.22)	−0.26
Female (Death)	3.01 (2.34–4.08)	3.4 (2.72–4.41)	0.13 (0.16–0.08)	0.28 (0.22–0.38)	0.23 (0.19–0.3)	−0.6
Male (Death)	10.06 (8.48–12.63)	13.4 (11.23–16.05)	0.33 (0.32–0.27)	0.91 (0.77–1.14)	0.89 (0.74–1.06)	−0.21

### 3.2 SDI regional level

**Figure 2:**
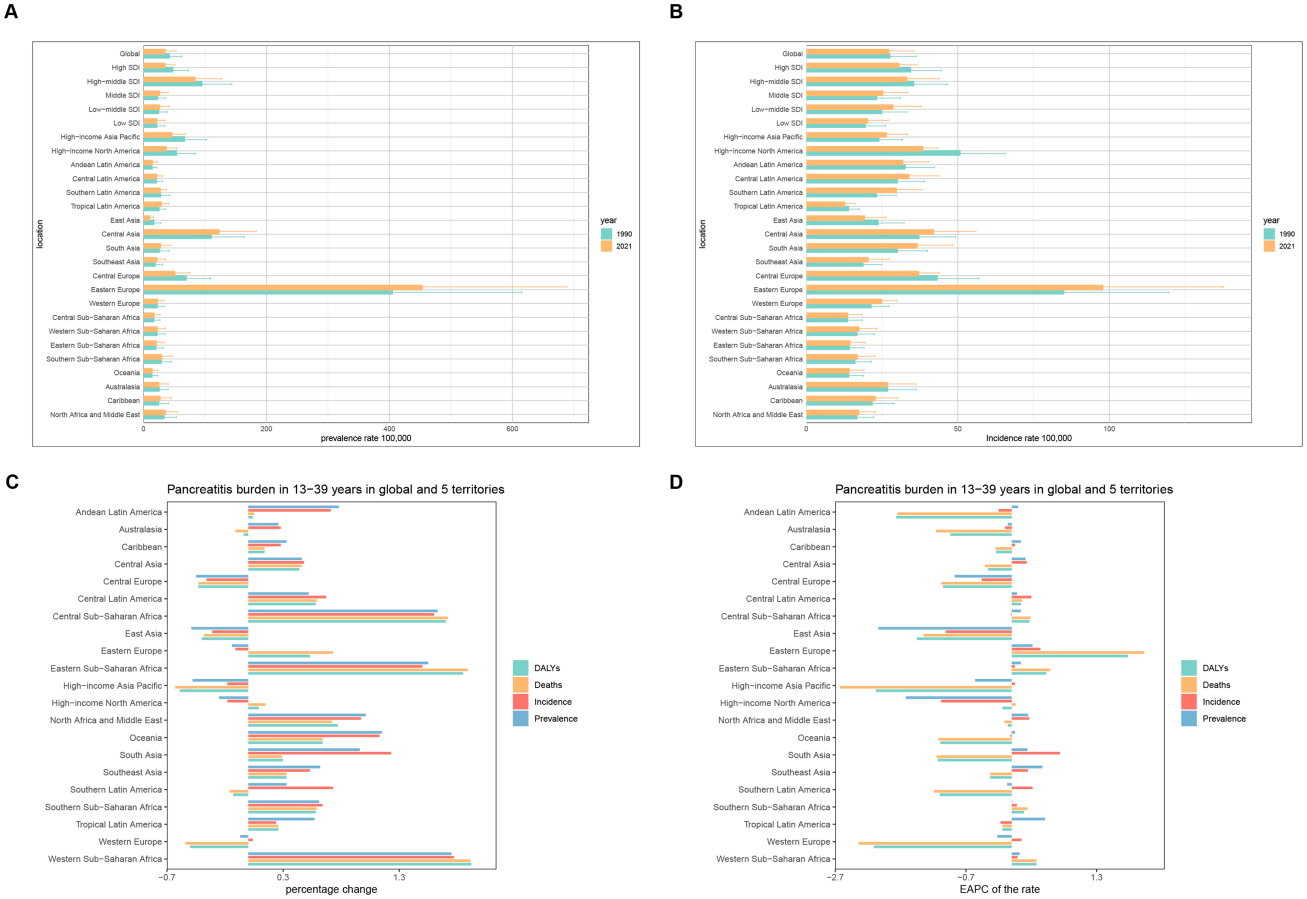
Temporal trend of Pancreatitis burden in 15–39 years in regions. **(A,B)** Prevalence and incidence rate per 100,000 population in 1990 and 2021. **(C)** Percentage change in cases of prevalent, incident, DALYs, and Deaths in 1990 and 2021. **(D)** EAPC of rates of prevalent, incident, DALYs, and Deaths from 1990 to 2021.

Among the five SDI groups globally, the Low SDI, Low-middle SDI, and Middle SDI regions experienced a significant increase in pancreatitis prevalence and incidence among individuals aged 15–39 years. The Low SDI region showed the most pronounced increases, with prevalence and incidence cases rising by 147 and 153%, respectively. In contrast, High-middle SDI and High SDI regions showed declining trends in prevalence and incidence, with the High SDI region exhibiting the largest reductions, as prevalence cases decreased by 14% and incidence cases by 9% (Figures 1C–F; Table 1; Supplementary Tables 1–3). For DALYs and deaths, only the High SDI region demonstrated a decreasing trend, with both measures declining by 22%. Conversely, the Low SDI region experienced the most significant increases in DALYs and deaths, with growth rates of 96 and 95%, respectively, marking the highest increases among all SDI groups. Analysis of EAPC for prevalence and incidence rates revealed upward trends in the Low SDI, Low-middle SDI, and Middle SDI regions. The most notable increase in prevalence rates occurred in the Low SDI region, with an EAPC of 0.37 (95% CI: 0.22 to 0.53), while the Low-middle SDI region had the highest growth in incidence rates, with an EAPC of 0.52 (95% CI: 0.47 to 0.57). Corresponding growth in prevalence and incidence cases was 147, 87, and 39%, respectively. For DALYs and mortality rates, the High SDI region showed the steepest declines, with EAPCs of −1.07 (95% CI: −1.12 to −0.95) for DALYs and −1.11 (95% CI: −1.24 to −0.97) for mortality. In contrast, the High-middle SDI region exhibited slight increases in DALYs and mortality rates, with EAPCs of 0.26 (95% CI: −0.11 to 0.63) and 0.38 (95% CI: −0.02 to 0.78), respectively (Figures 2A,B; Table 1; Supplementary Tables 1–3).

### 3.3 GBD regional level

Over the past 32 years (1990–2021), pancreatitis prevalence and incidence decreased in five regions: East Asia, High-income Asia Pacific, Central Europe, High-income North America, and Western Europe. Among these, East Asia experienced the most significant reduction in incidence, with a 49% decrease, while Central Europe had the largest reduction in prevalence, at 36%. Additionally, High-income Asia Pacific, Western Europe, Central Europe, East Asia, Southern Latin America, and Australasia showed declining trends in DALYs and deaths. The most substantial declines in DALYs and deaths were observed in High-income Asia Pacific, with reductions of 59 and 63%, respectively. In contrast, Western Sub-Saharan Africa experienced marked increases in prevalence, incidence, DALYs, and deaths, with growth rates of 175, 177, 192, and 191%, respectively.

Prevalence and incidence trends showed that, among the 21 regions, prevalence increased in 14 regions and decreased in 7. Tropical Latin America exhibited the highest increase in prevalence, with an EAPC of 0.51 (95% CI: 0.38–0.63), while East Asia showed the most significant decline, with an EAPC of −2.04 (95% CI: −2.71 to −1.37). Regarding incidence, 13 regions showed increasing trends, while 8 regions showed decreasing trends. The most notable increase in incidence was observed in South Asia, with an EAPC of 0.74 (95% CI: 0.65–0.82), whereas High-income North America demonstrated the greatest decline, with an EAPC of −1.08 (95% CI: −1.30 to −0.85).

DALYs and mortality rates exhibited distinct regional patterns. Among the 21 regions, 16 showed declining DALYs rates, while 6 exhibited increases. Western Europe experienced the most pronounced decline in DALYs rates, with an EAPC of −2.11 (95% CI: −2.21 to −2.00), whereas Eastern Europe had the highest increase, with an EAPC of 1.78 (95% CI: 1.16–2.41). Similarly, mortality rates decreased in 14 regions and increased in 7. High-income Asia Pacific recorded the largest decline in mortality rates, with an EAPC of −2.63 (95% CI: −2.82 to −2.43), while Eastern Europe exhibited the highest increase, with an EAPC of 2.03 (95% CI: 1.35–2.71) (Figures 2C,D; Table 1; Supplementary Tables 1–3).

### 3.4 Countries level

**Figure 3:**
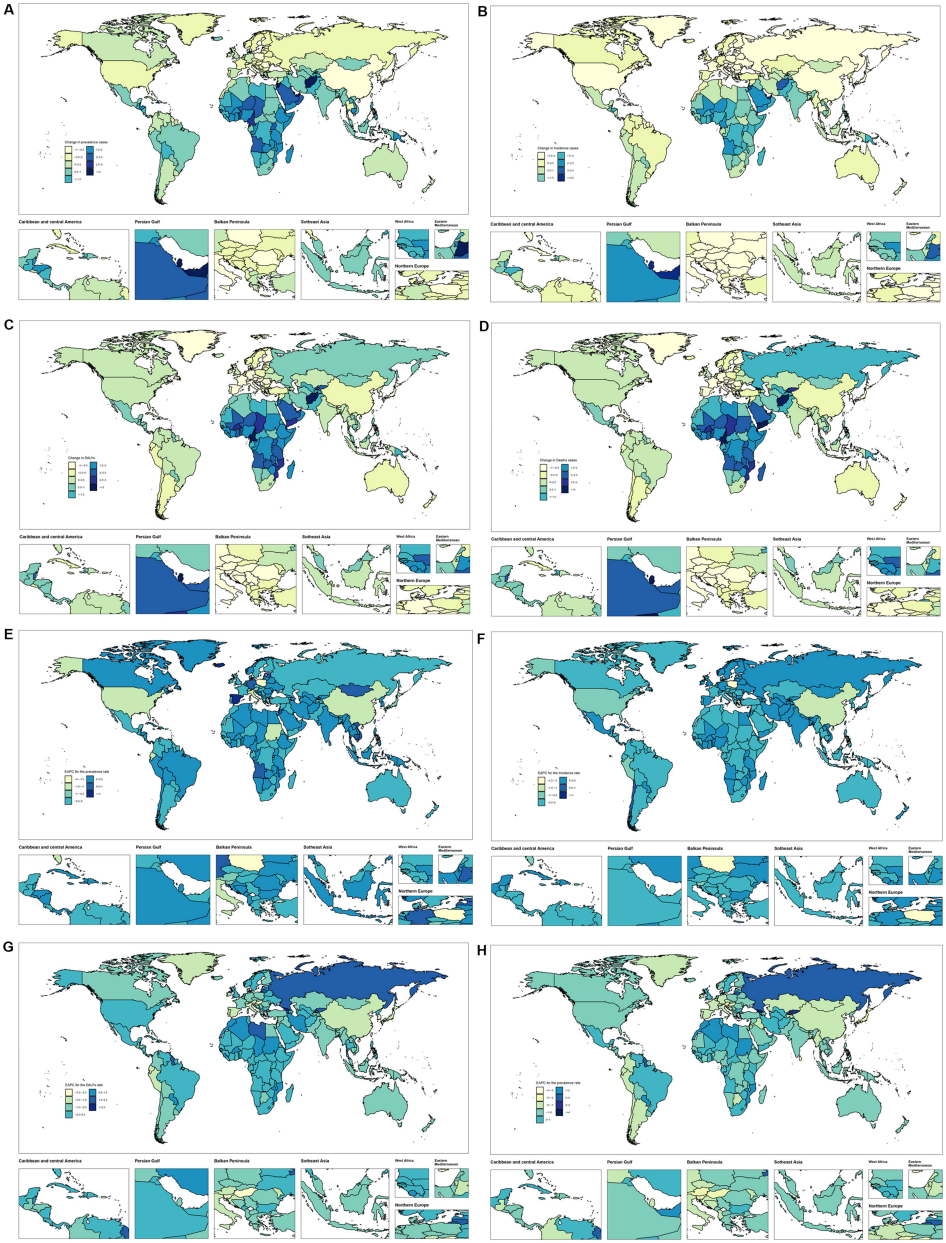
Temporal trend of Pancreatitis burden in 15–39 years globally. **(A,B)** Percentage change in prevalence cases and EAPC in prevalence rates across 204 countries in 1990 and 2021. **(C,D)** Percentage change in incidence cases and EAPC in incidence rates across 204 countries in 1990 and 2021. **(E,F)** Percentage change in DALYs cases and EAPC in DALYs rates across 204 countries in 1990 and 2021. **(G,H)** Percentage change in death cases and EAPC in death rates across 204 countries in 1990 and 2021.

From 1990 to 2021, among the 152 countries or territories analyzed globally, pancreatitis prevalence showed mixed trends, with increases in 152 countries and decreases in another 152 countries (Figures 3A,B and Supplementary Tables 4–7). The Cook Islands demonstrated the most significant increase in prevalence, with an EAPC of 1.48 (95% CI: 1.42–1.55), while Poland exhibited the largest decrease, with an EAPC of −2.60 (95% CI: −3.25 to −1.94). Regarding incidence, 126 countries showed increasing trends, while 74 exhibited declines (Figures 3C,D). The highest increase in incidence was observed in Chile, with an EAPC of 1.21 (95% CI: 1.00–1.42), whereas the steepest decline occurred in Hungary, with an EAPC of −1.55 (95% CI: −2.00 to −1.10). For DALYs rates and mortality rates, mixed trends were also evident. Among 152 countries, DALYs rates increased in 79 and decreased in 122 (Figures 3E,F). The Cook Islands had the most significant increase in DALYs rates, with an EAPC of 4.76 (95% CI: 4.30–5.23), while Hungary showed the largest decline, with an EAPC of −4.15 (95% CI: −4.68 to −3.61). Mortality rates increased in 80 countries and decreased in 122 (Figures 3G,H). The highest increase in mortality was observed in Georgia, with an EAPC of 8.61 (95% CI: 7.56–9.68), while Singapore exhibited the most substantial decline, with an EAPC of −4.64 (95% CI: −5.00 to −4.28).

### 3.5 Age patterns

**Figure 4:**
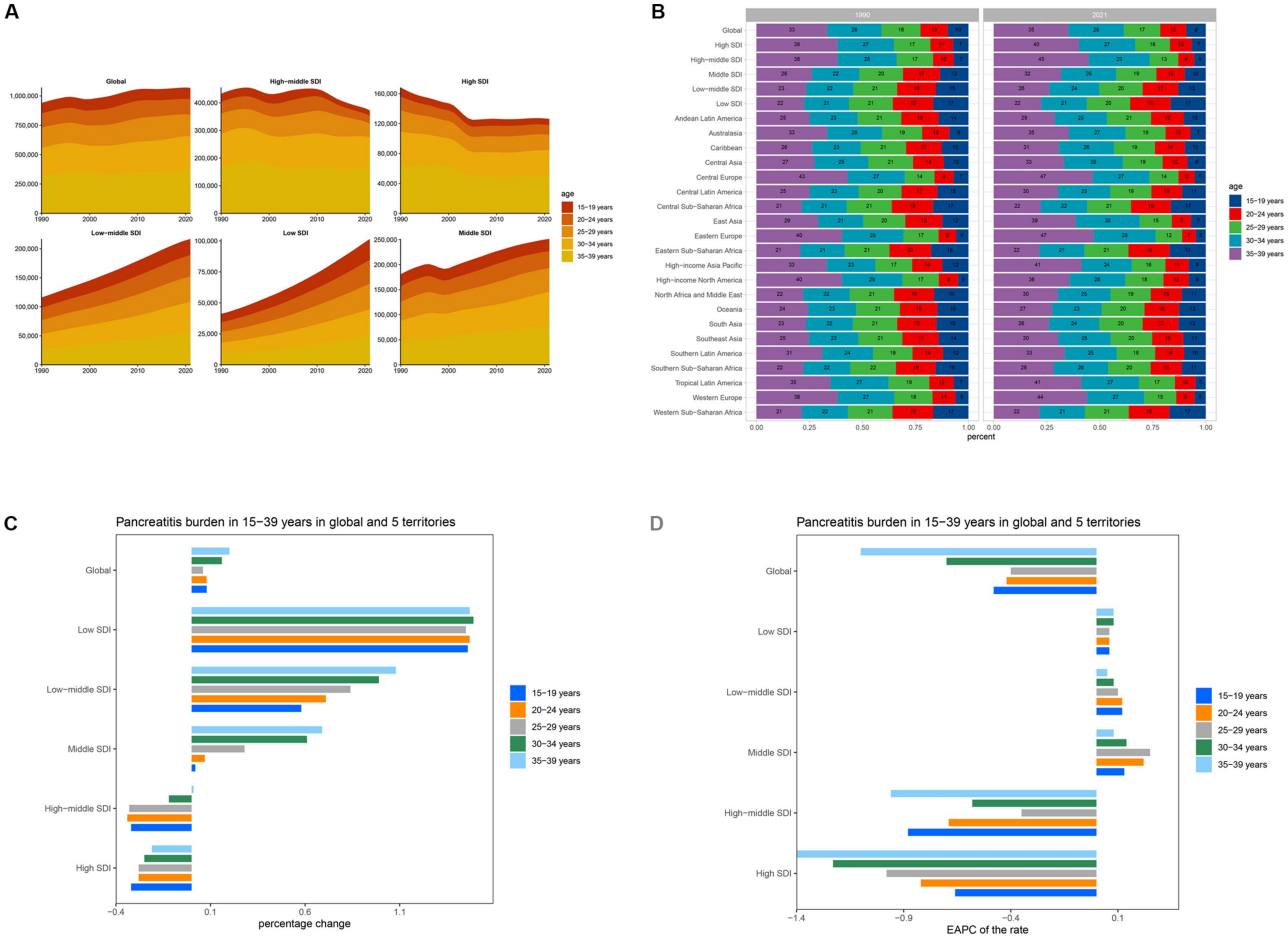
Temporal trend of Pancreatitis burden in 15–39 years by age pattern in different regions. **(A)** Prevalent cases of 5 age groups (15–39 years, 5-year intervals) from 1990 to 2021 globally and in 5 territories. **(B)** The distribution of prevalent cases across 5 age groups as percentages globally, in 5 territories, and 21 GBD regions in 1990 and 2021. **(C)** Percentage change in prevalent cases of 5 age groups globally and in 5 territories in 1990 and 2021. **(D)** EAPC of prevalent rates of 5 age groups globally and in 5 territories from 1990 to 2021.

From 1990 to 2021, the number of pancreatitis cases among individuals aged 35–39 years experienced the most significant global increase, rising by 20% (Table 3 and Figure 4A). Within different SDI groups, the High SDI and High-middle SDI regions showed a declining trend in case numbers, with the 20–24 age group in High-middle SDI regions exhibiting the largest decrease, at −34%. In contrast, the Low SDI, Low-middle SDI, and Middle SDI regions showed increasing trends, with the 30–34 age group in Low SDI regions experiencing the most substantial rise, at 149% (Figure 4B). Globally, the 30–34 age group exhibited the largest growth in case numbers, at 39% (Figure 4C). Regarding DALYs and deaths, individuals aged 35–39 years showed the greatest global increases, with growth rates of 42 and 43%, respectively. Conversely, the 15–19 age group experienced decreases in DALYs and deaths, with reductions of −1% and −3%, respectively (Supplementary Tables 8–10).

**Table 3:** The prevalence of pancreatitis burden in people aged 15–39 years in global and 5 cases and rates, and the trends in age patterns from 1990 to 2021.

location	Age (year)	Prevalence cases			Prevalence rates		
		1990 thousand (95%UI)	2021 thousand (95%UI)	Percentage Change (100%)	1990 per (95%UI)	2021 per (95%UI)	EAPC (95% CI)
Global	15–19 years	91.43 (52.18–149.43)	98.62 (56.66–161.3)	0.08 (0.09–0.08)	17.6 (10.05–28.77)	15.8 (9.08–25.85)	−0.48 (−0.53–−0.44)
Global	15–39 years	940.26 (602.13–1374.39)	1069.42 (688.08–1574.63)	0.14 (0.14–0.15)	42.9 (27.47–62.71)	35.95 (23.13–52.93)	−0.58 (−0.65–−0.51)
Global	20–24 years	121.19 (73.32–198.39)	130.96 (80.62–213.75)	0.08 (0.1–0.08)	24.63 (14.9–40.32)	21.93 (13.5–35.79)	−0.42 (−0.52–−0.31)
Global	25–29 years	172.6 (103.23–274.33)	183.1 (110.19–294.67)	0.06 (0.07–0.07)	39 (23.32–61.98)	31.12 (18.73–50.08)	−0.4 (−0.53–−0.27)
Global	30–34 years	240.95 (142.95–384.48)	279.09 (169.1–443.78)	0.16 (0.18–0.15)	62.52 (37.09–99.76)	46.17 (27.97–73.42)	−0.7 (−0.9–−0.51)
Global	35–39 years	314.08 (180.19–539.25)	377.65 (224.85–623.04)	0.2 (0.25–0.16)	89.17 (51.15–153.09)	67.33 (40.09–111.08)	−1.1 (−1.3–−0.9)
Global	15–19 years	6.85 (3.46–11.89)	16.88 (8.71–28.83)	1.46 (1.52–1.42)	13.53 (6.83–23.47)	13.61 (7.03–23.25)	0.06 (0.05–0.08)
Global	15–39 years	41.13 (24.6–63.4)	101.61 (60.79–157.74)	1.47 (1.47–1.49)	22.31 (13.35–34.4)	22.63 (13.54–35.13)	0.09 (0.07–0.1)
Low SDI	20–24 years	7.84 (4.26–13.22)	19.39 (10.44–32.6)	1.47 (1.45–1.47)	18.39 (9.99–31.01)	18.59 (10.01–31.26)	0.06 (0.05–0.08)
Low SDI	25–29 years	8.48 (4.6–14.65)	20.78 (11.27–35.98)	1.45 (1.45–1.46)	23.67 (12.85–40.9)	24.12 (13.08–41.77)	0.06 (0.05–0.08)
Low SDI	30–34 years	8.75 (4.92–14.6)	21.78 (12.13–36.86)	1.49 (1.47–1.52)	29.46 (16.56–49.15)	30.08 (16.75–50.92)	0.08 (0.07–0.08)
Low SDI	35–39 years	9.21 (5.04–15.92)	22.79 (12.63–39.33)	1.47 (1.51–1.47)	36.09 (19.75–62.42)	36.6 (20.29–63.16)	0.08 (0.07–0.1)
Low SDI	15–19 years	17.79 (9.58–29.96)	28.19 (15.43–48.08)	0.58 (0.61–0.6)	14.96 (8.06–25.2)	15.28 (8.36–26.06)	0.12 (0.11–0.14)
Low SDI	15–39 years	116.09 (71.93–176.25)	216.96 (134.26–333.99)	0.87 (0.87–0.89)	25.6 (15.86–38.87)	27.04 (16.73–41.62)	0.19 (0.17–0.22)
Low SDI	20–24 years	21.29 (12.23–36)	36.5 (21.07–61.51)	0.71 (0.72–0.71)	20.42 (11.73–34.52)	20.88 (12.05–35.19)	0.12 (0.1–0.14)
Low SDI	25–29 years	24.04 (13.54–40.64)	44.18 (25.01–75.65)	0.84 (0.85–0.86)	26.82 (15.11–45.34)	27.29 (15.45–46.74)	0.1 (0.08–0.13)
Low-middle SDI	30–34 years	25.69 (14.69–42.87)	51.23 (29.11–86.08)	0.99 (0.98–1.01)	34.02 (19.45–56.78)	34.66 (19.69–58.23)	0.08 (0.05–0.11)
Low-middle SDI	35–39 years	27.28 (15.64–45.93)	56.86 (32.69–95.51)	1.08 (1.09–1.08)	41.92 (24.03–70.58)	42.61 (24.5–71.58)	0.05 (0.03–0.08)
Low-middle SDI	15–19 years	24.37 (13.39–40.16)	24.77 (14.09–40.19)	0.02 (0.05–0)	13.01 (7.15–21.44)	13.58 (7.73–22.04)	0.13 (−0.02–0.27)
Low-middle SDI	15–39 years	180.91 (115.06–272.06)	251.4 (159.93–375.83)	0.39 (0.39–0.38)	24.04 (15.29–36.15)	27.11 (17.24–40.52)	0.37 (0.22–0.53)
Low-middle SDI	20–24 years	31.57 (18.43–52.51)	33.88 (20.75–55.32)	0.07 (0.13–0.05)	17.7 (10.34–29.45)	19.11 (11.71–31.21)	0.22 (0.12–0.32)
Low-middle SDI	25–29 years	37.08 (21.29–61.77)	47.42 (28.3–77.42)	0.28 (0.33–0.25)	24.56 (14.1–40.92)	25.8 (15.4–42.12)	0.25 (0.09–0.4)
Low-middle SDI	30–34 years	40.58 (23.34–66.89)	65.48 (38.88–106.22)	0.61 (0.67–0.59)	33.11 (19.04–54.58)	32.82 (19.49–53.25)	0.14 (−0.1–0.38)
Low-middle SDI	35–39 years	47.31 (27.61–79.2)	79.86 (47.02–129.81)	0.69 (0.7–0.64)	41.7 (24.33–69.81)	43.26 (25.47–70.31)	0.08 (−0.16–0.32)
Middle SDI	15–19 years	30.03 (17.69–49.16)	20.32 (12.17–33.43)	−0.32 (−0.31--0.32)	31.11 (18.32–50.92)	28.04 (16.79–46.13)	−0.88 (−1.11--0.65)
Middle SDI	15–39 years	433.03 (275.71–650.85)	372.38 (240.15–558.54)	−0.14 (−0.13--0.14)	95.69 (60.93–143.82)	84.58 (54.55–126.86)	−0.4 (−0.48–−0.32)
Middle SDI	20–24 years	42.39 (25.26–69.59)	28.15 (16.98–45.61)	−0.34 (−0.33--0.34)	43.44 (25.89–71.32)	37.54 (22.65–60.84)	−0.69 (−1.08–−0.3)
Middle SDI	25–29 years	74.06 (43.57–124.03)	49.88 (30.12–82.82)	−0.33 (−0.31–−0.33)	79.7 (46.88–133.47)	58.9 (35.57–97.8)	−0.35 (−0.72–0.03)
Middle SDI	30–34 years	120.84 (70.34–189.37)	106.91 (63.83–164.93)	−0.12 (−0.09–−0.13)	141.74 (82.51–222.12)	100.2 (59.82–154.57)	−0.58 (−0.9–−0.25)
Middle SDI	35–39 years	165.7 (96.07–279.01)	167.12 (98.85–276.9)	0.01 (0.03–−0.01)	206.53 (119.75–347.76)	164.75 (97.45–272.99)	−0.96 (−1.24–−0.69)
Middle SDI	15–19 years	12.3 (7.06–20.27)	8.4 (5.06–13.12)	−0.32 (−0.28–−0.35)	18.78 (10.77–30.93)	13.96 (8.41–21.8)	−0.66 (−0.84–−0.48)
Middle SDI	15–39 years	168.29 (104.65–253.52)	126.35 (83.78–181.24)	−0.25 (−0.2–−0.29)	48.5 (30.16–73.07)	35.77 (23.72–51.31)	−1.11 (−1.29–−0.93)
High-middle SDI	20–24 years	18.01 (10.57–29.26)	12.96 (8.38–20.09)	−0.28 (−0.21–−0.31)	26.15 (15.34–42.47)	19.82 (12.81–30.72)	−0.82 (−0.97–−0.66)
High-middle SDI	25–29 years	28.82 (16.56–46.14)	20.73 (13.19–32.52)	−0.28 (−0.2–−0.3)	39.53 (22.72–63.3)	29.04 (18.47–45.56)	−0.98 (−1.13–−0.84)
High-middle SDI	30–34 years	44.88 (25.34–71.54)	33.5 (20.33–51.59)	−0.25 (−0.2–−0.28)	62.31 (35.17–99.31)	43.17 (26.2–66.48)	−1.23 (−1.39–−1.08)
High-middle SDI	35–39 years	64.28 (37.55–110.71)	50.76 (32.09–82.53)	−0.21 (−0.15–−0.25)	95.03 (55.51–163.67)	64.53 (40.8–104.92)	−1.4 (−1.58–−1.22)
High-middle SDI	15–19 years	91.43 (52.18–149.43)	98.62 (56.66–161.3)	0.08 (0.09–0.08)	17.6 (10.05–28.77)	15.8 (9.08–25.85)	−0.48 (−0.53–−0.44)
High-middle SDI	15–39 years	940.26 (602.13–1374.39)	1069.42 (688.08–1574.63)	0.14 (0.14–0.15)	42.9 (27.47–62.71)	35.95 (23.13–52.93)	−0.58 (−0.65–−0.51)
High-middle SDI	20–24 years	121.19 (73.32–198.39)	130.96 (80.62–213.75)	0.08 (0.1–0.08)	24.63 (14.9–40.32)	21.93 (13.5–35.79)	−0.42 (−0.52–−0.31)
High-middle SDI	25–29 years	172.6 (103.23–274.33)	183.1 (110.19–294.67)	0.06 (0.07–0.07)	39 (23.32–61.98)	31.12 (18.73–50.08)	−0.4 (−0.53–−0.27)
High SDI	30–34 years	240.95 (142.95–384.48)	279.09 (169.1–443.78)	0.16 (0.18–0.15)	62.52 (37.09–99.76)	46.17 (27.97–73.42)	−0.7 (−0.9–−0.51)
High SDI	35–39 years	314.08 (180.19–539.25)	377.65 (224.85–623.04)	0.2 (0.25–0.16)	89.17 (51.15–153.09)	67.33 (40.09–111.08)	−1.1 (−1.3–−0.9)
High SDI	15–19 years	6.85 (3.46–11.89)	16.88 (8.71–28.83)	1.46 (1.52–1.42)	13.53 (6.83–23.47)	13.61 (7.03–23.25)	0.06 (0.05–0.08)
High SDI	15–39 years	41.13 (24.6–63.4)	101.61 (60.79–157.74)	1.47 (1.47–1.49)	22.31 (13.35–34.4)	22.63 (13.54–35.13)	0.09 (0.07–0.1)
High SDI	20–24 years	7.84 (4.26–13.22)	19.39 (10.44–32.6)	1.47 (1.45–1.47)	18.39 (9.99–31.01)	18.59 (10.01–31.26)	0.06 (0.05–0.08)
High SDI	25–29 years	8.48 (4.6–14.65)	20.78 (11.27–35.98)	1.45 (1.45–1.46)	23.67 (12.85–40.9)	24.12 (13.08–41.77)	0.06 (0.05–0.08)
High SDI	30–34 years	8.75 (4.92–14.6)	21.78 (12.13–36.86)	1.49 (1.47–1.52)	29.46 (16.56–49.15)	30.08 (16.75–50.92)	0.08 (0.07–0.08)
High SDI	35–39 years	9.21 (5.04–15.92)	22.79 (12.63–39.33)	1.47 (1.51–1.47)	36.09 (19.75–62.42)	36.6 (20.29–63.16)	0.08 (0.07–0.1)

In terms of prevalence rates, the 35–39 age group exhibited the most significant decline globally, with an EAPC of −1.1 (95% CI: −1.3 to −0.9). Analyzing by SDI group, only the High SDI and High-middle SDI regions showed declining trends in incidence rates, with the 35–39 age group in High SDI regions showing the steepest decline, at an EAPC of −1.4 (95% CI: −1.58 to −1.22) (Table 3 and Figure 4D). In contrast, incidence rates increased among the 15–19 and 20–24 age groups, with EAPCs of 0.33 (95% CI: 0.31 to 0.36) and 0.13 (95% CI: 0.10 to 0.16), respectively. Across the Low SDI, Low-middle SDI, and Middle SDI regions, incidence rates generally increased, with the 25–29 age group in Low SDI regions exhibiting the most notable rise, at an EAPC of 0.25 (95% CI: 0.09 to 0.40). For DALYs and mortality rates, overall declines were observed globally, with the 20–24 age group showing the most significant reductions. The EAPCs were −0.67 (95% CI: −0.89 to −0.46) for DALYs and −0.71 (95% CI: −0.93 to −0.48) for mortality (Supplementary Tables 8–10).

### 3.6 The association between pancreatitis burden and SDI

**Figure 5:**
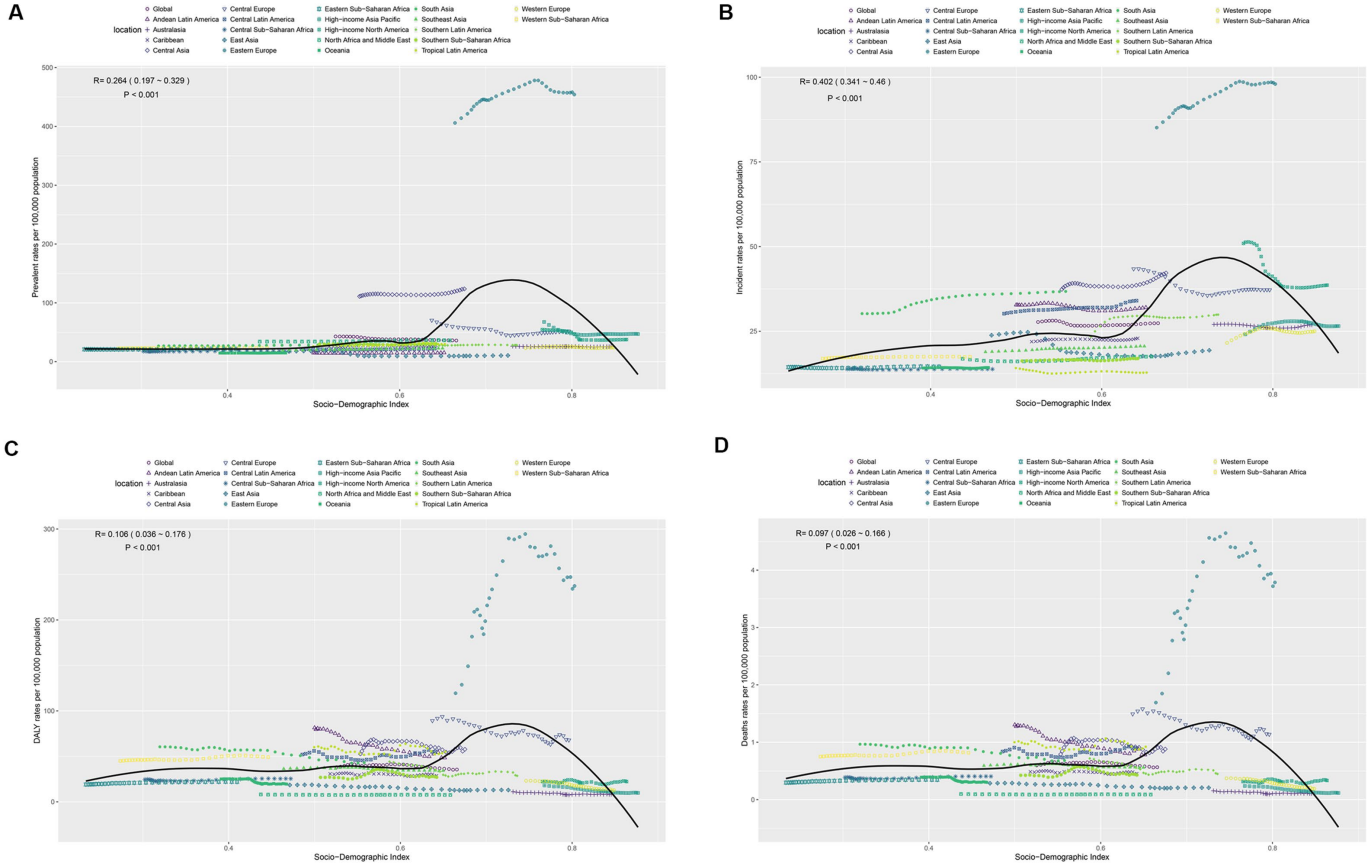
**(A–D)** Respectively represent the associations between the SDI and prevalence, incidence, DALYs and deaths rates per 100,000 population of Pancreatitis burden in 15–39 years across 21 GBD regions.

The relationship between pancreatitis prevalence among individuals aged 35–39 years and the SDI exhibits an inverted U-shaped trend. In low SDI regions, prevalence rates are relatively low, likely due to underdiagnosis and limited healthcare resources. In middle SDI regions, prevalence rates rise sharply, reflecting the combined effects of lifestyle changes and improved diagnostic capabilities. In high SDI regions, prevalence rates decrease, potentially due to better preventive measures and effective health management. Overall, a mild positive correlation was observed between SDI and prevalence (*R* = 0.264, *p* < 0.001), with significant regional differences (Figure 5A).

Incidence rates showed a stronger correlation with SDI (*R* = 0.402, *p* < 0.001) and also followed an inverted U-shaped trend, peaking in middle SDI regions and declining in high SDI regions. This pattern reflects the interplay of lifestyle factors, healthcare accessibility, and effective health management (Figure 5B). The correlation between disease burden (DALY rates) and SDI was weaker (*R* = 0.106, *p* < 0.001), and mortality rates showed an even lower correlation (*R* = 0.097, *p* < 0.001). Both DALY and mortality rates demonstrated a slight increase in middle SDI regions but a decline in high SDI regions (Figures 5C,D). These findings highlight significant disparities in disease burden and mortality across SDI regions, emphasizing the need for tailored prevention and treatment strategies to reduce disease-related burden and mortality based on regional characteristics.

## 4 Discussion

Based on the GBD 2021 database, this study analyzed the disease burden of pancreatitis in the population aged 15–39, discovered significant regional differences and temporal variations of pancreatitis, and provided important insights for formulating targeted prevention and management strategies.

First, it was found that the prevalence and incidence of pancreatitis showed a non-linear “inverted U-shaped” relationship with SDI, peaking in areas with medium SDI and being lower in areas with low and high SDI. The low values of prevalence in low SDI areas may be related to insufficient healthcare resources and low diagnostic rates, and there may be massive underdiagnosis of pancreatitis in these areas (17). In addition, lifestyle changes in low SDI areas, such as the prevalence of alcohol consumption and high-fat diets, may also contribute to an underestimation of the actual disease burden. In intermediate SDI areas, increased incidence and prevalence may reflect improved medical diagnostic capacity and increased lifestyle risk factors in the course of socioeconomic development (18). In contrast, prevalence and morbidity have declined in high SDI areas as a result of lifestyle interventions and improved health management, emphasizing the positive role of health policies in disease prevention (19).

Although the overall burden of pancreatitis has increased, population-adjusted DALYs rates and mortality rates have declined in most regions. This suggests that while the number of cases has risen, improvements in disease management and treatment outcomes, particularly in high SDI regions, have played a critical role in mitigating the impact. High SDI regions have benefited from advanced medical technologies, enhanced acute-phase care, and effective chronic disease management, including early imaging-based diagnosis, antibiotic therapy, and endoscopic or surgical interventions. These measures have significantly reduced case fatality rates and long-term disability (20). In contrast, low SDI regions face critical challenges, including limited access to imaging, lack of specialist care, and low public awareness of pancreatitis symptoms. Targeted actions in these areas should include strengthening primary care systems, training frontline health workers in early recognition and referral protocols, and increasing the availability of essential diagnostics. Community-level health education campaigns can also raise awareness about modifiable risk factors and improve treatment-seeking behavior. These tailored approaches are essential to narrowing the global health gap in pancreatitis care and outcomes.

Age-stratified analysis revealed that individuals aged 35–39 years bear the highest disease burden of pancreatitis. The sharp rise in pancreatitis cases among individuals aged 35–39 years may be attributed to prolonged and cumulative exposure to major lifestyle risk factors, including excessive alcohol consumption, high-fat diets, smoking, and obesity. This age group typically enters a life stage characterized by increased occupational stress and metabolic burden, which may further exacerbate the risk. Moreover, increased access to healthcare and diagnostic improvements may lead to higher detection rates in this population (21). Previous studies have shown that both acute and chronic pancreatitis are strongly associated with modifiable behavioral and metabolic factors, which tend to peak in prevalence during mid-adulthood. These findings underscore the importance of targeted prevention strategies and early lifestyle interventions for individuals in their 30 (22).

This trend is further confirmed by long-term changes from 1990 to 2021. Globally, despite an increase in the overall number of cases, high-income regions are experiencing a significant downward trend in EAPC due to advances in medical technology and improved health management, while low-income regions continue to experience an increase in the number of cases and burden of disease. This result highlights the severity of the unequal distribution of healthcare resources internationally and the importance of global health cooperation and resource sharing.

The gender differences observed in our study—higher prevalence in females but greater mortality and DALY rates in males—are consistent with previous epidemiological findings. Several factors may underlie these disparities. First, males tend to have higher rates of alcohol use and smoking, both of which are strong risk factors for severe or necrotizing pancreatitis. Second, delayed presentation to medical care and lower health-seeking behavior among males may result in worse clinical outcomes. Finally, hormonal and immunological differences between sexes may also influence disease progression and severity. However, the precise pathophysiological mechanisms remain poorly understood and warrant further investigation through future studies focused on sex-specific risk factors and clinical trajectories.

Taken together with the most recent literature, these results are consistent with the overall trend in the global burden of pancreatitis. Alcohol consumption, high-fat diets and obesity remain the main risk factors for pancreatitis, while other potential risk factors such as genetic background and environmental pollution are increasingly being emphasized. In addition, lifestyle interventions adopted in recent years in some high-income areas, such as limiting alcohol consumption, promoting healthy diets, and early intervention techniques for severe cases, may have contributed significantly to the decline in the burden of disease. In contrast, the inadequacy of healthcare systems in many low-income areas remains an important reason for the high burden of disease.

This study still has some limitations. First, although advanced statistical modeling techniques including Bayesian meta-regression were applied in the GBD study, due to the limited monitoring system, underreporting and poor diagnostic capabilities, the data in some low-income areas may be biased, which may undermine the accuracy of burden estimation. Second, this study did not delve into the specific contributions of lifestyle, environmental factors, and genetic background to pancreatitis, issues that could be the focus of future research. In addition, there are certain limitations in using EAPC for analysis. EAPC relies on the assumption of a logarithmic linear relationship between the incidence rate and time, which may not accurately capture the non-linearity or sudden changes in the disease burden.

## 5 Conclusion

This study systematically analyzed the Global Burden of Disease and its dynamic trends in pancreatitis among young people aged 15–39 years using the GBD 2021 database, revealing a non-linear relationship between incidence, DALYs rates and mortality rates and SDIs, showing significant regional and age differences. The burden of disease in low SDI regions continues to increase, while high SDI regions show a decreasing trend due to improved medical resources and health management, reflecting the importance of medical resource allocation and preventive measures. In the future, the equitable distribution of global health resources should be strengthened and individualized disease prevention and control strategies should be developed for different SDI regions to reduce the global health burden of pancreatitis.

## Data Availability

The original contributions presented in the study are included in the article/Supplementary material, further inquiries can be directed to the corresponding author.

## Footnotes

^1^ https://ghdx.healthdata.org/gbd-2021/sources
