# Supplementary material for: Global burden of pancreatitis among individuals aged 15–39 years: a systematic analysis from the 2021 GBD study
Source: Front Med (Lausanne). 2025 May 27;12:1572346. doi: 10.3389/fmed.2025.1572346 (PMC12150401; doi:10.3389/fmed.2025.1572346)
Supplement: Supplementary file 1 [file Supplementary_file_1.docx]

**Supplementary Table 1** The incidence of pancreatitis burden in people aged 15-39 years in global and 5 cases and rates, and the trends from 1990 to 2021

| **location** | **Incidence cases** | | | **Incidence rates** | | |
| --- | --- | --- | --- | --- | --- | --- |
|  | **1990 thousand (95%UI)** | **2021 thousand**  **(95%UI)** | **percentage**  **Change**  **(100%)** | **1990**  **per (95%UI)** | **2021**  **per (95%UI)** | **EAPC**  **(95% CI)** |
| Andean Latin America | 5.07 (3.72-6.55) | 8.67 (6.69-10.92) | 0.71 | 32.78 (24.08-42.34) | 32.02 (24.7-40.33) | -0.2 (-0.26--0.14) |
| Australasia | 2.2 (1.56-2.96) | 2.82 (2.03-3.78) | 0.28 | 27.03 (19.13-36.28) | 26.9 (19.41-36.06) | -0.1 (-0.15--0.04) |
| Caribbean | 3.26 (2.31-4.29) | 4.17 (2.99-5.5) | 0.28 | 21.93 (15.56-28.89) | 22.92 (16.43-30.21) | 0.05 (0.01-0.08) |
| Central Asia | 10.63 (7.59-14) | 15.78 (11.32-20.94) | 0.48 | 37.37 (26.69-49.21) | 42.21 (30.28-56.01) | 0.23 (0.15-0.32) |
| Central Europe | 20.34 (14.76-26.66) | 13.03 (10.9-15.38) | -0.36 | 43.41 (31.5-56.91) | 37.22 (31.12-43.91) | -0.46 (-0.64--0.29) |
| Central Latin America | 20.61 (15.18-26.62) | 34.44 (25.93-44.07) | 0.67 | 30.19 (22.24-39) | 34.04 (25.63-43.56) | 0.3 (0.26-0.34) |
| Central Sub-Saharan Africa | 2.87 (1.99-3.84) | 7.47 (5.14-9.86) | 1.6 | 13.83 (9.57-18.5) | 13.82 (9.5-18.23) | -0.01 (-0.04-0.02) |
| East Asia | 134.79 (92.94-182.53) | 92.82 (66.17-125.72) | -0.31 | 23.83 (16.43-32.27) | 19.38 (13.81-26.24) | -1.01 (-1.32--0.7) |
| Eastern Europe | 73.01 (51.39-102.65) | 64.84 (45.44-91.09) | -0.11 | 85.12 (59.91-119.68) | 97.98 (68.67-137.65) | 0.44 (0.38-0.49) |
| Eastern Sub-Saharan Africa | 10.21 (7.1-13.54) | 25.51 (17.76-33.93) | 1.5 | 14.4 (10.02-19.1) | 14.56 (10.14-19.37) | 0.05 (0.01-0.09) |
| Global | 607.83 (438.15-796.81) | 814.5 (606.27-1056.9) | 0.34 | 27.73 (19.99-36.35) | 27.38 (20.38-35.53) | -0.09 (-0.16--0.03) |
| High-income Asia Pacific | 16.27 (11.36-21.44) | 13.41 (10.26-16.91) | -0.18 | 24.1 (16.83-31.77) | 26.53 (20.3-33.45) | 0.05 (-0.09-0.18) |
| High-income North America | 57.72 (43.7-74.28) | 47.57 (41.45-53.49) | -0.18 | 50.94 (38.56-65.55) | 38.62 (33.65-43.42) | -1.08 (-1.3--0.85) |
| High-middle SDI | 161.24 (117.85-211.31) | 146.87 (109.23-192.98) | -0.09 | 35.63 (26.04-46.69) | 33.36 (24.81-43.83) | -0.28 (-0.38--0.18) |
| High SDI | 119.94 (89.45-154.65) | 108.59 (89.31-129.22) | -0.09 | 34.57 (25.78-44.57) | 30.74 (25.28-36.58) | -0.66 (-0.8--0.52) |
| Low-middle SDI | 113.78 (79.98-150.99) | 230.94 (161.97-304.2) | 1.03 | 25.09 (17.64-33.3) | 28.78 (20.18-37.91) | 0.52 (0.47-0.57) |
| Low SDI | 36.15 (25.25-48.08) | 91.54 (64.55-121.73) | 1.53 | 19.61 (13.7-26.09) | 20.39 (14.38-27.11) | 0.19 (0.16-0.22) |
| Middle SDI | 176.17 (125.46-234.13) | 236.02 (171.53-310.32) | 0.34 | 23.41 (16.67-31.11) | 25.45 (18.49-33.46) | 0.21 (0.11-0.3) |
| North Africa and Middle East | 22.59 (15.78-29.71) | 44.6 (31.7-57.85) | 0.97 | 16.88 (11.79-22.2) | 17.54 (12.47-22.75) | 0.27 (0.21-0.34) |
| Oceania | 0.38 (0.26-0.5) | 0.81 (0.56-1.08) | 1.13 | 14.16 (9.94-18.88) | 14.29 (10-19.1) | -0.02 (-0.04-0) |
| South Asia | 130.39 (91.75-172.76) | 290.77 (204.03-381.99) | 1.23 | 30.21 (21.26-40.03) | 36.76 (25.8-48.3) | 0.74 (0.65-0.82) |
| Southeast Asia | 37.25 (26.35-49.2) | 57.07 (40.77-75.84) | 0.53 | 18.91 (13.38-24.97) | 20.58 (14.7-27.35) | 0.25 (0.23-0.27) |
| Southern Latin America | 4.46 (3.41-5.66) | 7.71 (5.89-9.85) | 0.73 | 23.36 (17.89-29.66) | 29.9 (22.84-38.19) | 0.32 (0.15-0.48) |
| Southern Sub-Saharan Africa | 3.51 (2.45-4.64) | 5.77 (3.99-7.68) | 0.64 | 16.24 (11.32-21.47) | 16.96 (11.74-22.57) | 0.08 (0.04-0.12) |
| Tropical Latin America | 9.1 (7.07-11.22) | 11.31 (8.56-14.25) | 0.24 | 14.14 (10.99-17.44) | 12.81 (9.7-16.13) | -0.17 (-0.27--0.07) |
| Western Europe | 31.09 (23.77-39.29) | 32.46 (26.66-38.82) | 0.04 | 21.57 (16.5-27.26) | 25.02 (20.54-29.92) | 0.15 (0-0.3) |
| Western Sub-Saharan Africa | 12.09 (8.39-16.08) | 33.47 (23.3-44.39) | 1.77 | 16.89 (11.73-22.46) | 17.5 (12.19-23.22) | 0.09 (0.07-0.11) |
