# Supplementary material for: Global burden of pancreatitis among individuals aged 15–39 years: a systematic analysis from the 2021 GBD study
Source: Front Med (Lausanne). 2025 May 27;12:1572346. doi: 10.3389/fmed.2025.1572346 (PMC12150401; doi:10.3389/fmed.2025.1572346)
Supplement: Supplementary file 2 [file Supplementary_file_2.docx]

**Supplementary Table 2** The DALYs of pancreatitis burden in people aged 15-39 years in global and 5 cases and rates, and the trends from 1990 to 2021

| **location** | **DALYs cases** | | | **DALYs rates** | | |
| --- | --- | --- | --- | --- | --- | --- |
|  | **1990 thousand**  **(95%UI)** | **2021 thousand**  **(95%UI)** | **percentage**  **Change**  **(100%)** | **1990**  **per (95%UI)** | **2021**  **per (95%UI)** | **EAPC**  **(95% CI)** |
| Andean Latin America | 12.59 (10.26-15.38) | 13.1 (10.27-16.61) | 0.04 | 81.42 (66.36-99.49) | 48.39 (37.92-61.35) | -1.77 (-1.89--1.65) |
| Australasia | 0.91 (0.8-1.06) | 0.87 (0.72-1.04) | -0.04 | 11.15 (9.76-13.04) | 8.26 (6.87-9.96) | -0.94 (-1.16--0.71) |
| Caribbean | 4.58 (3.99-5.24) | 5.21 (4.14-6.57) | 0.14 | 30.82 (26.84-35.24) | 28.64 (22.74-36.1) | -0.24 (-0.34--0.14) |
| Central Asia | 14.99 (13.15-17.24) | 21.61 (18.3-25.79) | 0.44 | 52.7 (46.21-60.6) | 57.79 (48.95-68.98) | -0.36 (-0.63--0.09) |
| Central Europe | 41.8 (39.21-44.63) | 23.79 (21.9-25.72) | -0.43 | 89.22 (83.7-95.26) | 67.93 (62.55-73.43) | -1.05 (-1.24--0.86) |
| Central Latin America | 35.52 (33.93-37.06) | 56.12 (51.34-61.39) | 0.58 | 52.03 (49.7-54.28) | 55.47 (50.75-60.69) | 0.14 (-0.09-0.38) |
| Central Sub-Saharan Africa | 5.11 (3.24-8.54) | 13.78 (8.92-22.39) | 1.7 | 24.63 (15.58-41.12) | 25.47 (16.48-41.39) | 0.27 (0.14-0.39) |
| East Asia | 104.74 (86.33-128.64) | 62.34 (48.24-81.74) | -0.4 | 18.51 (15.26-22.74) | 13.01 (10.07-17.06) | -1.45 (-1.6--1.3) |
| Eastern Europe | 102.43 (90.79-121.75) | 157.08 (135.95-182.38) | 0.53 | 119.43 (105.85-141.95) | 237.38 (205.44-275.6) | 1.78 (1.16-2.41) |
| Eastern Sub-Saharan Africa | 13.52 (8.31-20.57) | 38.52 (23.06-55.2) | 1.85 | 19.07 (11.72-29.02) | 21.99 (13.16-31.51) | 0.53 (0.49-0.57) |
| Global | 836.49 (728.56-1002.3) | 1056.43 (936.94-1238.99) | 0.26 | 38.16 (33.24-45.73) | 35.51 (31.5-41.65) | -0.33 (-0.47--0.2) |
| High-income Asia Pacific | 12.19 (9.62-15.37) | 4.99 (4.09-6.32) | -0.59 | 18.06 (14.26-22.77) | 9.88 (8.09-12.51) | -2.08 (-2.27--1.9) |
| High-income North America | 25.27 (22.95-28.86) | 27.46 (25.43-30.15) | 0.09 | 22.3 (20.26-25.47) | 22.29 (20.64-24.48) | -0.14 (-0.36-0.07) |
| High-middle SDI | 198.11 (180.38-222.27) | 224.56 (199.13-253.59) | 0.13 | 43.78 (39.86-49.12) | 51.01 (45.23-57.6) | 0.26 (-0.11-0.63) |
| High SDI | 89.51 (82.56-99.43) | 70.01 (64.71-77.06) | -0.22 | 25.8 (23.79-28.66) | 19.82 (18.32-21.81) | -1.07 (-1.2--0.95) |
| Low-middle SDI | 238.44 (188.1-330.62) | 330.45 (269.23-412.68) | 0.39 | 52.59 (41.49-72.92) | 41.18 (33.55-51.42) | -0.81 (-0.92--0.69) |
| Low SDI | 76.49 (54.25-105.76) | 149.57 (112.42-200.76) | 0.96 | 41.5 (29.43-57.38) | 33.31 (25.04-44.71) | -0.79 (-0.87--0.71) |
| Middle SDI | 232.93 (206.14-276.4) | 281.06 (244.72-327.01) | 0.21 | 30.95 (27.39-36.72) | 30.3 (26.39-35.26) | -0.08 (-0.12--0.04) |
| North Africa and Middle East | 10.98 (8.15-14.6) | 19.42 (14.94-25.98) | 0.77 | 8.2 (6.09-10.91) | 7.64 (5.88-10.22) | -0.06 (-0.13-0.01) |
| Oceania | 0.67 (0.3-1.07) | 1.1 (0.61-1.76) | 0.64 | 25.11 (11.47-40.33) | 19.51 (10.91-31.22) | -1.09 (-1.25--0.92) |
| South Asia | 261.27 (202.12-376.04) | 340.15 (267.13-420.35) | 0.3 | 60.53 (46.83-87.12) | 43.01 (33.77-53.15) | -1.13 (-1.31--0.94) |
| Southeast Asia | 71.56 (54.2-101.37) | 95.12 (71.71-144.47) | 0.33 | 36.32 (27.51-51.46) | 34.3 (25.86-52.09) | -0.33 (-0.41--0.26) |
| Southern Latin America | 8.44 (7.76-9.12) | 7.38 (6.76-8.03) | -0.13 | 44.26 (40.67-47.83) | 28.61 (26.22-31.12) | -1.1 (-1.45--0.75) |
| Southern Sub-Saharan Africa | 5.82 (4.22-7.5) | 9.22 (6.93-12.19) | 0.58 | 26.92 (19.54-34.7) | 27.1 (20.35-35.82) | 0.19 (-0.2-0.59) |
| Tropical Latin America | 38.64 (36.68-40.66) | 48.62 (45.46-51.71) | 0.26 | 60.08 (57.04-63.22) | 55.06 (51.48-58.55) | -0.14 (-0.38-0.11) |
| Western Europe | 33.27 (31.31-35.54) | 16.59 (14.99-18.53) | -0.5 | 23.09 (21.73-24.66) | 12.78 (11.55-14.28) | -2.11 (-2.21--2) |
| Western Sub-Saharan Africa | 32.19 (20.74-52.28) | 93.95 (64.92-127.27) | 1.92 | 44.97 (28.98-73.05) | 49.13 (33.95-66.56) | 0.38 (0.31-0.46) |
