# Supplementary material for: Global burden of pancreatitis among individuals aged 15–39 years: a systematic analysis from the 2021 GBD study
Source: Front Med (Lausanne). 2025 May 27;12:1572346. doi: 10.3389/fmed.2025.1572346 (PMC12150401; doi:10.3389/fmed.2025.1572346)
Supplement: Supplementary file 3 [file Supplementary_file_3.docx]

**Supplementary Table 3** The deaths of pancreatitis burden in people aged 15-39 years in global and 5 cases and rates, and the trends from 1990 to 2021

| **location** | **Deaths cases** | | | **Deaths rates** | | |
| --- | --- | --- | --- | --- | --- | --- |
|  | **1990**  **(95%UI)** | **2021**  **(95%UI)** | **percentage**  **Change**  **(100%)** | **1990**  **per (95%UI)** | **2021**  **per (95%UI)** | **EAPC**  **(95% CI)** |
| Andean Latin America | 201.65 (164.96-246.83) | 211.53 (163.97-271.64) | 0.05 | 1.3 (1.07-1.6) | 0.78 (0.61-1) | -1.75 (-1.88--1.63) |
| Australasia | 12.56 (11.48-13.82) | 11.19 (9.67-12.83) | -0.11 | 0.15 (0.14-0.17) | 0.11 (0.09-0.12) | -1.16 (-1.45--0.87) |
| Caribbean | 72.25 (62.45-82.7) | 82.22 (64.22-104.84) | 0.14 | 0.49 (0.42-0.56) | 0.45 (0.35-0.58) | -0.25 (-0.36--0.14) |
| Central Asia | 225.2 (201.51-253.98) | 329.13 (278.01-385.65) | 0.46 | 0.79 (0.71-0.89) | 0.88 (0.74-1.03) | -0.41 (-0.72--0.1) |
| Central Europe | 699.4 (659.46-740.29) | 398.29 (369.2-430.61) | -0.43 | 1.49 (1.41-1.58) | 1.14 (1.05-1.23) | -1.08 (-1.28--0.88) |
| Central Latin America | 572.29 (548.16-593.51) | 911.84 (829.48-993.9) | 0.59 | 0.84 (0.8-0.87) | 0.9 (0.82-0.98) | 0.16 (-0.09-0.41) |
| Central Sub-Saharan Africa | 80.53 (48.29-138.22) | 218.85 (136.6-363.26) | 1.72 | 0.39 (0.23-0.67) | 0.4 (0.25-0.67) | 0.29 (0.16-0.43) |
| East Asia | 1608.75 (1291.14-1958.96) | 991.16 (743.34-1339.44) | -0.38 | 0.28 (0.23-0.35) | 0.21 (0.16-0.28) | -1.35 (-1.52--1.18) |
| Eastern Europe | 1450.72 (1392.12-1516.72) | 2504.89 (2174.76-2782.15) | 0.73 | 1.69 (1.62-1.77) | 3.79 (3.29-4.2) | 2.03 (1.35-2.71) |
| Eastern Sub-Saharan Africa | 209.65 (124.28-327.09) | 605.19 (348.66-888.59) | 1.89 | 0.3 (0.18-0.46) | 0.35 (0.2-0.51) | 0.59 (0.55-0.63) |
| Global | 13069.17 (11366.38-15762.89) | 16799.91 (14826.03-19749.17) | 0.29 | 0.6 (0.52-0.72) | 0.56 (0.5-0.66) | -0.29 (-0.44--0.15) |
| High-income Asia Pacific | 160.69 (125.36-190.91) | 58.85 (52.08-67.75) | -0.63 | 0.24 (0.19-0.28) | 0.12 (0.1-0.13) | -2.63 (-2.82--2.43) |
| High-income North America | 356.19 (346.13-369.11) | 410.41 (389.7-428.35) | 0.15 | 0.31 (0.31-0.33) | 0.33 (0.32-0.35) | 0.06 (-0.19-0.3) |
| High-middle SDI | 2972.72 (2807.32-3206.13) | 3570.79 (3199.19-3968.81) | 0.2 | 0.66 (0.62-0.71) | 0.81 (0.73-0.9) | 0.38 (-0.02-0.78) |
| High SDI | 1352.08 (1283.93-1404.9) | 1057.09 (1018.24-1102.8) | -0.22 | 0.39 (0.37-0.4) | 0.3 (0.29-0.31) | -1.11 (-1.24--0.97) |
| Low-middle SDI | 3816.18 (2975.04-5335.11) | 5283.06 (4281.03-6597.72) | 0.38 | 0.84 (0.66-1.18) | 0.66 (0.53-0.82) | -0.81 (-0.92--0.69) |
| Low SDI | 1229.87 (848.44-1726.6) | 2394.37 (1766.5-3228.14) | 0.95 | 0.67 (0.46-0.94) | 0.53 (0.39-0.72) | -0.8 (-0.88--0.72) |
| Middle SDI | 3681.65 (3222.81-4392.21) | 4482.1 (3875.57-5245.12) | 0.22 | 0.49 (0.43-0.58) | 0.48 (0.42-0.57) | -0.06 (-0.1--0.02) |
| North Africa and Middle East | 131.6 (100.8-178.57) | 226.36 (178.43-286.84) | 0.72 | 0.1 (0.08-0.13) | 0.09 (0.07-0.11) | -0.11 (-0.2--0.02) |
| Oceania | 10.36 (4.47-16.75) | 16.97 (9.07-27.65) | 0.64 | 0.39 (0.17-0.63) | 0.3 (0.16-0.49) | -1.12 (-1.28--0.95) |
| South Asia | 4168.06 (3170.68-6034.63) | 5382.7 (4127.22-6634.32) | 0.29 | 0.97 (0.73-1.4) | 0.68 (0.52-0.84) | -1.15 (-1.34--0.96) |
| Southeast Asia | 1165.37 (880.77-1685.74) | 1555.15 (1154.14-2405.97) | 0.33 | 0.59 (0.45-0.86) | 0.56 (0.42-0.87) | -0.33 (-0.41--0.25) |
| Southern Latin America | 136.89 (126.1-148.35) | 115.35 (105.88-124.8) | -0.16 | 0.72 (0.66-0.78) | 0.45 (0.41-0.48) | -1.19 (-1.57--0.81) |
| Southern Sub-Saharan Africa | 91.69 (65.88-118.85) | 145.87 (106.06-198.4) | 0.59 | 0.42 (0.3-0.55) | 0.43 (0.31-0.58) | 0.24 (-0.19-0.67) |
| Tropical Latin America | 643.67 (612.68-676.02) | 813.44 (766.79-860.52) | 0.26 | 1 (0.95-1.05) | 0.92 (0.87-0.97) | -0.14 (-0.39-0.11) |
| Western Europe | 535.42 (511.57-560.04) | 247.8 (231.55-264.1) | -0.54 | 0.37 (0.35-0.39) | 0.19 (0.18-0.2) | -2.34 (-2.46--2.22) |
| Western Sub-Saharan Africa | 536.25 (335.95-900.33) | 1562.69 (1071.3-2160.38) | 1.91 | 0.75 (0.47-1.26) | 0.82 (0.56-1.13) | 0.38 (0.3-0.46) |
