# Supplementary material for: Global burden of pancreatitis among individuals aged 15–39 years: a systematic analysis from the 2021 GBD study
Source: Front Med (Lausanne). 2025 May 27;12:1572346. doi: 10.3389/fmed.2025.1572346 (PMC12150401; doi:10.3389/fmed.2025.1572346)
Supplement: Supplementary file 4 [file Supplementary_file_4.docx]

**Supplementary Table 4** The prevalence of pancreatitis cases and rates among aged 15-39 years in 1990 and 2021 across 204 countries, and the trends from 1990 to 2021

| **location** | **Prevalence cases** | | | **Prevalence rates** | | |
| --- | --- | --- | --- | --- | --- | --- |
|  | **1990 thousand**  **(95%UI)** | **2021 thousand**  **(95%UI)** | **percentage**  **Change**  **(100%)** | **1990**  **per (95%UI)** | **2021**  **per (95%UI)** | **EAPC**  **(95% CI)** |
| Afghanistan | 841.81 (510.84-1292.17) | 3597.02 (2213.09-5506.38) | 3.27 | 26.73 (16.22-41.03) | 29.43 (18.11-45.06) | 0.34 (0.14-0.54) |
| Albania | 604.31 (348.13-933.44) | 476.03 (279.36-737.61) | -0.21 | 42.54 (24.51-65.71) | 50.21 (29.47-77.8) | 0.29 (0.21-0.36) |
| Algeria | 3082.01 (1830.79-4957.61) | 5927.88 (3399.56-9104.82) | 0.92 | 30.5 (18.12-49.06) | 34.81 (19.97-53.47) | 0.46 (0.41-0.51) |
| American Samoa | 2.89 (1.62-4.67) | 2.56 (1.48-4.03) | -0.11 | 14.29 (8.02-23.09) | 14.64 (8.47-23.09) | -0.01 (-0.06-0.04) |
| Andorra | 5.67 (3.28-8.94) | 5.43 (3.14-8.58) | -0.04 | 22.65 (13.13-35.74) | 21.28 (12.32-33.66) | -0.26 (-0.4--0.12) |
| Angola | 695.98 (383.38-1087.7) | 2314.34 (1301.12-3569.9) | 2.33 | 17.79 (9.8-27.8) | 19.02 (10.69-29.34) | 0.54 (0.4-0.68) |
| Antigua and Barbuda | 6.25 (3.66-9.57) | 9.94 (5.75-16.13) | 0.59 | 24.25 (14.19-37.12) | 28.89 (16.72-46.89) | 0.47 (0.38-0.56) |
| Argentina | 4705.21 (2756.54-7244.63) | 6213.07 (4315.58-8510.05) | 0.32 | 38.52 (22.57-59.31) | 35.46 (24.63-48.57) | -0.3 (-0.38--0.21) |
| Armenia | 1598.56 (966.58-2374.56) | 1303.94 (859.79-1843.81) | -0.18 | 111.23 (67.25-165.22) | 121.29 (79.98-171.51) | 0.11 (-0.04-0.27) |
| Australia | 1880.17 (1118.64-2944.88) | 2352.11 (1319.3-3752.8) | 0.25 | 27.76 (16.52-43.48) | 27.13 (15.22-43.29) | -0.07 (-0.1--0.04) |
| Austria | 1400.76 (1215.77-1615.27) | 825.56 (706.23-958.99) | -0.41 | 46.66 (40.5-53.81) | 29.25 (25.02-33.98) | -1.86 (-2.08--1.64) |
| Azerbaijan | 3636.22 (2098.36-5456.9) | 5773.7 (3344.12-8483) | 0.59 | 114.41 (66.03-171.7) | 136.3 (78.95-200.26) | 0.41 (0.3-0.53) |
| Bahamas | 34.19 (20.02-53.52) | 41.81 (24.63-66.78) | 0.22 | 28.97 (16.97-45.35) | 27.03 (15.92-43.18) | -0.27 (-0.32--0.22) |
| Bahrain | 107.4 (64.83-166.5) | 268.88 (159.48-412.64) | 1.5 | 41.9 (25.29-64.95) | 38.22 (22.67-58.65) | -0.09 (-0.2-0.03) |
| Bangladesh | 7367.51 (4234.98-11310.19) | 12351.65 (7425.94-18922.39) | 0.68 | 17.45 (10.03-26.8) | 17.95 (10.79-27.5) | 0.11 (0.09-0.12) |
| Barbados | 30.6 (17.99-48.22) | 31.59 (18.18-50.95) | 0.03 | 28.04 (16.48-44.18) | 31.98 (18.4-51.58) | 0.37 (0.35-0.4) |
| Belarus | 11712.8 (7099.66-17797.43) | 10283.92 (6205.82-15885) | -0.12 | 296.94 (179.99-451.2) | 350.55 (211.54-541.47) | 0.39 (0.31-0.48) |
| Belgium | 1526.09 (933.16-2245.57) | 1677.88 (1307.53-2128.5) | 0.1 | 41.02 (25.08-60.36) | 47.9 (37.33-60.77) | -0.02 (-0.27-0.23) |
| Belize | 18.18 (10.77-28.22) | 50.31 (29.53-80.26) | 1.77 | 24.85 (14.72-38.57) | 26.65 (15.64-42.51) | 0.26 (0.22-0.3) |
| Benin | 291.78 (160.19-446.23) | 905.15 (516.16-1405.17) | 2.1 | 17.14 (9.41-26.21) | 17.27 (9.85-26.8) | 0.02 (0-0.04) |
| Bermuda | 9.21 (5.41-14.39) | 5.99 (3.49-9.7) | -0.35 | 35.83 (21.02-55.96) | 34.22 (19.95-55.39) | -0.34 (-0.42--0.27) |
| Bhutan | 50.29 (29.3-77.66) | 65.71 (38.02-101.92) | 0.31 | 18.66 (10.87-28.82) | 18.96 (10.97-29.41) | -0.08 (-0.14--0.03) |
| Bolivia (Plurinational State of) | 388.42 (231.44-595.42) | 830.97 (468.58-1310.12) | 1.14 | 15.75 (9.38-24.14) | 16.91 (9.54-26.66) | 0.21 (0.16-0.27) |
| Bosnia and Herzegovina | 932.86 (544.48-1439.66) | 590.74 (335.16-915.9) | -0.37 | 49.12 (28.67-75.81) | 58.71 (33.31-91.02) | 0.46 (0.36-0.57) |
| Botswana | 92.53 (53.37-143.89) | 217.4 (122.16-340.43) | 1.35 | 17.98 (10.37-27.95) | 20.41 (11.47-31.95) | 0.46 (0.43-0.5) |
| Brazil | 16209.59 (11815.12-21968.71) | 25321.93 (18050.85-35019.52) | 0.56 | 25.83 (18.83-35.01) | 29.7 (21.17-41.08) | 0.52 (0.4-0.65) |
| Brunei Darussalam | 49.25 (28.1-73.68) | 82.07 (46.17-125.7) | 0.67 | 39.95 (22.8-59.76) | 40.22 (22.63-61.61) | -0.03 (-0.1-0.04) |
| Bulgaria | 1732.89 (1001.25-2707.17) | 1373.05 (790.19-2113.76) | -0.21 | 58.21 (33.63-90.94) | 72.28 (41.6-111.27) | 0.81 (0.68-0.94) |
| Burkina Faso | 578.5 (320.49-892.55) | 1687.68 (960.77-2600.82) | 1.92 | 18.15 (10.05-28) | 19.5 (11.1-30.05) | 0.26 (0.23-0.29) |
| Burundi | 461.64 (265.75-740.9) | 991.35 (549.98-1548.74) | 1.15 | 22.27 (12.82-35.74) | 18.8 (10.43-29.38) | -0.6 (-0.71--0.49) |
| Cabo Verde | 23 (12.94-35.79) | 52.44 (30.19-81.49) | 1.28 | 17.59 (9.9-27.37) | 20.93 (12.04-32.52) | 0.47 (0.42-0.53) |
| Cambodia | 534.97 (307.36-836.76) | 1384.17 (810.55-2130.56) | 1.59 | 13.89 (7.98-21.73) | 19.11 (11.19-29.41) | 0.96 (0.78-1.13) |
| Cameroon | 710.63 (392.09-1117.8) | 2568.71 (1429.18-4199.51) | 2.61 | 18.68 (10.31-29.39) | 19.92 (11.09-32.57) | 0.32 (0.26-0.38) |
| Canada | 4631.16 (2772.95-7394.02) | 5003.89 (2934.22-7763.55) | 0.08 | 41.66 (24.94-66.51) | 42.19 (24.74-65.45) | -0.04 (-0.1-0.01) |
| Central African Republic | 186.2 (107.82-288.44) | 371.27 (205.52-583.54) | 0.99 | 17.88 (10.35-27.7) | 17.01 (9.41-26.73) | -0.09 (-0.12--0.06) |
| Chad | 352.92 (205.73-549.64) | 1071.25 (611.9-1638.64) | 2.04 | 16.81 (9.8-26.18) | 17.02 (9.72-26.04) | 0.11 (0.08-0.15) |
| Chile | 468.42 (395.23-565.24) | 741.67 (650.26-847.63) | 0.58 | 8.18 (6.9-9.87) | 10.48 (9.19-11.98) | 0.74 (0.64-0.85) |
| China | 97764.02 (57900.52-155012.18) | 48405.89 (30184.42-75050.52) | -0.5 | 17.84 (10.56-28.28) | 10.49 (6.54-16.26) | -2.1 (-2.79--1.41) |
| Colombia | 3513.69 (2172.4-5297.88) | 5002.22 (2994.27-7507.22) | 0.42 | 25 (15.46-37.7) | 24.89 (14.9-37.36) | -0.11 (-0.17--0.05) |
| Comoros | 27.6 (15.59-43.13) | 53.12 (31.44-81.85) | 0.92 | 15.98 (9.03-24.97) | 17.15 (10.15-26.42) | 0.25 (0.23-0.28) |
| Congo | 166.59 (95.31-254.34) | 433.17 (245.35-689.65) | 1.6 | 17.58 (10.06-26.85) | 19.55 (11.07-31.13) | 0.48 (0.42-0.54) |
| Cook Islands | 1.13 (0.66-1.77) | 1.28 (0.74-2.03) | 0.13 | 14.67 (8.61-22.95) | 21.78 (12.63-34.46) | 1.48 (1.42-1.55) |
| Costa Rica | 327.34 (206.26-486.58) | 492.6 (310.84-763.97) | 0.5 | 25.48 (16.06-37.88) | 25.88 (16.33-40.14) | -0.14 (-0.24--0.05) |
| C么te d'Ivoire | 871.47 (492.04-1378.74) | 2225.53 (1273.1-3540.78) | 1.55 | 18.42 (10.4-29.15) | 19.85 (11.36-31.58) | 0.3 (0.26-0.34) |
| Croatia | 383.17 (311.72-476.18) | 273.62 (232.11-325.28) | -0.29 | 21.12 (17.18-26.24) | 21.92 (18.6-26.06) | -0.05 (-0.14-0.04) |
| Cuba | 1253.68 (731.3-1997.82) | 1057.36 (608.9-1712.53) | -0.16 | 25.69 (14.99-40.94) | 29.49 (16.98-47.76) | 0.21 (0.05-0.38) |
| Cyprus | 24.24 (14.35-38.04) | 36.91 (24.03-55.8) | 0.52 | 7.88 (4.67-12.37) | 7.36 (4.79-11.12) | 0.03 (-0.07-0.14) |
| Czechia | 2698.22 (1626.9-4096.89) | 1722.27 (1247.77-2337.91) | -0.36 | 72.72 (43.84-110.41) | 58.32 (42.25-79.17) | 0.17 (-0.19-0.54) |
| Democratic People's Republic of Korea | 816.28 (473.63-1328.75) | 1100.12 (649.13-1711.83) | 0.35 | 9.79 (5.68-15.93) | 10.94 (6.45-17.02) | 0.17 (0.1-0.24) |
| Democratic Republic of the Congo | 2499.67 (1455.72-3884.92) | 6197.79 (3487.18-9511.56) | 1.48 | 17.45 (10.16-27.12) | 17.18 (9.66-26.36) | -0.01 (-0.06-0.04) |
| Denmark | 470.69 (298.94-672.2) | 405.47 (263.47-581.95) | -0.14 | 24.67 (15.67-35.23) | 22.23 (14.45-31.91) | -0.59 (-0.81--0.37) |
| Djibouti | 29.1 (16.57-44.83) | 95.11 (54.46-146.64) | 2.27 | 16.6 (9.45-25.57) | 17.59 (10.07-27.12) | 0.2 (0.18-0.22) |
| Dominica | 7.71 (4.42-12.32) | 7.38 (4.32-11.94) | -0.04 | 26.41 (15.12-42.2) | 28.51 (16.68-46.11) | 0.06 (-0.05-0.16) |
| Dominican Republic | 761.39 (437.79-1181.56) | 1259.22 (744.82-2004.64) | 0.65 | 24.78 (14.25-38.46) | 27.69 (16.38-44.08) | 0.21 (0.14-0.28) |
| Ecuador | 476.54 (367.77-599.49) | 595.92 (529.83-677.99) | 0.25 | 11.55 (8.91-14.53) | 8.16 (7.25-9.28) | -0.88 (-1.05--0.71) |
| Egypt | 6852.88 (4166.82-10861.99) | 13694.99 (8282.32-21415.88) | 1 | 31.26 (19.01-49.55) | 32.44 (19.62-50.73) | 0.17 (0.13-0.22) |
| El Salvador | 442.48 (279.53-664.72) | 594.85 (370.24-914.78) | 0.34 | 21.18 (13.38-31.82) | 22.95 (14.28-35.29) | 0.16 (0.11-0.2) |
| Equatorial Guinea | 26.1 (15.33-40.83) | 137.73 (77.84-211.91) | 4.28 | 17.28 (10.15-27.04) | 19.8 (11.19-30.46) | 0.54 (0.5-0.58) |
| Eritrea | 214.8 (117.76-337.01) | 481.29 (282.47-741.24) | 1.24 | 16.58 (9.09-26.01) | 17.19 (10.09-26.47) | 0.1 (0.08-0.12) |
| Estonia | 1651.86 (1001.22-2467.35) | 1474.69 (896.51-2275.88) | -0.11 | 290.83 (176.27-434.4) | 372.91 (226.7-575.51) | 1.02 (0.86-1.17) |
| Eswatini | 53.8 (30.14-83.38) | 101.29 (57.4-159.93) | 0.88 | 17.85 (10-27.67) | 19.89 (11.27-31.41) | 0.32 (0.26-0.37) |
| Ethiopia | 4672.99 (2768.44-7293.43) | 12445.02 (7298.2-19554.71) | 1.66 | 25.58 (15.15-39.92) | 26.85 (15.74-42.18) | 0.2 (0.15-0.24) |
| Fiji | 48.8 (28.41-76.46) | 58.01 (33.03-93.38) | 0.19 | 15.13 (8.81-23.71) | 16.26 (9.26-26.18) | 0.25 (0.2-0.31) |
| Finland | 631.62 (471.73-822.71) | 494.7 (359.97-669.42) | -0.22 | 34.79 (25.98-45.32) | 29.69 (21.61-40.18) | -0.35 (-0.43--0.28) |
| France | 4888.33 (2883.8-7558.42) | 3864.35 (2226.64-6151.75) | -0.21 | 22.22 (13.11-34.36) | 19.45 (11.21-30.97) | -0.42 (-0.47--0.37) |
| Gabon | 81.09 (46.78-130.93) | 152.8 (86.34-243.64) | 0.88 | 21.08 (12.16-34.04) | 20.39 (11.52-32.5) | -0.06 (-0.08--0.03) |
| Gambia | 63.71 (37-100.48) | 173.68 (103.36-271.41) | 1.73 | 16.9 (9.81-26.65) | 17.37 (10.33-27.14) | 0.14 (0.1-0.18) |
| Georgia | 2416.82 (1466.91-3593.67) | 1512.7 (919.4-2234.37) | -0.37 | 113.52 (68.9-168.8) | 133.31 (81.03-196.91) | 0.5 (0.43-0.58) |
| Germany | 4994.63 (2995.92-8029.61) | 6275.32 (4084.14-8888.73) | 0.26 | 16.81 (10.08-27.03) | 24.81 (16.14-35.14) | -0.06 (-0.49-0.37) |
| Ghana | 1036.94 (581.11-1621.72) | 2676.05 (1533.41-4187.63) | 1.58 | 18.06 (10.12-28.25) | 18.71 (10.72-29.28) | 0.07 (0.04-0.09) |
| Greece | 955.11 (555.58-1522.97) | 961.45 (603.36-1376.72) | 0.01 | 25.41 (14.78-40.51) | 34.54 (21.67-49.45) | 0.14 (-0.18-0.46) |
| Greenland | 9.51 (5.59-15.26) | 7.58 (4.53-12.33) | -0.2 | 35.92 (21.13-57.67) | 37.17 (22.2-60.43) | -0.29 (-0.45--0.12) |
| Grenada | 8.97 (5.19-13.79) | 12.34 (7.15-19.55) | 0.38 | 26.9 (15.58-41.35) | 30.52 (17.69-48.37) | 0.26 (0.21-0.32) |
| Guam | 9.95 (5.73-15.89) | 9.08 (5.29-14.72) | -0.09 | 15.69 (9.03-25.06) | 16.38 (9.55-26.55) | 0.02 (-0.04-0.07) |
| Guatemala | 650.22 (409.84-968.19) | 1511.17 (910.97-2351.14) | 1.32 | 22.01 (13.87-32.78) | 22.2 (13.38-34.54) | 0.09 (0.03-0.16) |
| Guinea | 348.95 (201.55-539.49) | 859.96 (503.63-1316.87) | 1.46 | 16.99 (9.81-26.26) | 16.64 (9.75-25.49) | -0.07 (-0.1--0.03) |
| Guinea-Bissau | 65.15 (36.74-102.99) | 150.12 (84.04-226.32) | 1.3 | 17.57 (9.91-27.77) | 17.79 (9.96-26.82) | 0.08 (0.04-0.11) |
| Guyana | 96.15 (56.68-150.49) | 83.52 (49.76-130.2) | -0.13 | 28.25 (16.65-44.21) | 26.88 (16.02-41.9) | -0.34 (-0.42--0.26) |
| Haiti | 624.13 (364.95-1026.28) | 1481.3 (859.67-2400.79) | 1.37 | 25.64 (14.99-42.16) | 26.98 (15.66-43.74) | 0.17 (0.11-0.23) |
| Honduras | 368.09 (227.22-546.63) | 1006.14 (635.69-1536.73) | 1.73 | 21.32 (13.16-31.66) | 22.89 (14.46-34.96) | 0.17 (0.14-0.21) |
| Hungary | 2427.76 (1403.12-3748.39) | 1738.81 (996.92-2653.37) | -0.28 | 65.69 (37.96-101.42) | 63.19 (36.23-96.43) | 0.16 (0.02-0.3) |
| Iceland | 9.91 (5.95-16.14) | 19.5 (12.47-29.41) | 0.97 | 9.54 (5.72-15.54) | 16.3 (10.42-24.57) | 0.83 (0.54-1.12) |
| India | 96128.73 (57327.57-150543.02) | 184900.51 (111609.09-288476.31) | 0.92 | 28.19 (16.81-44.14) | 30.34 (18.31-47.33) | 0.26 (0.23-0.29) |
| Indonesia | 17952.24 (10451.31-28514.43) | 28077.24 (16494.01-45233.33) | 0.56 | 23 (13.39-36.54) | 24.66 (14.48-39.72) | 0.25 (0.23-0.27) |
| Iran (Islamic Republic of) | 9291.04 (5613.82-14310.49) | 17882.53 (10775.99-27705.24) | 0.92 | 42.78 (25.85-65.89) | 51.53 (31.05-79.83) | 0.71 (0.62-0.8) |
| Iraq | 2246.35 (1325.72-3462.84) | 5595.61 (3298.89-8856.1) | 1.49 | 31.26 (18.45-48.19) | 32.09 (18.92-50.79) | 0.15 (0.11-0.18) |
| Ireland | 260.77 (150.23-407.28) | 308.47 (178.35-481.91) | 0.18 | 19.01 (10.95-29.69) | 19.71 (11.4-30.8) | 0.14 (0.02-0.26) |
| Israel | 272.16 (161.96-436.42) | 503.38 (294.08-830.02) | 0.85 | 14.24 (8.48-22.84) | 15.15 (8.85-24.98) | 0.33 (0.27-0.38) |
| Italy | 3838.6 (2488.09-5621.08) | 1843.61 (1445.94-2356.39) | -0.52 | 17.98 (11.65-26.33) | 11.67 (9.15-14.92) | -1.2 (-1.34--1.05) |
| Jamaica | 234.14 (138.27-357.23) | 317.65 (187.72-517.22) | 0.36 | 23.82 (14.07-36.35) | 26.62 (15.73-43.35) | 0.22 (0.16-0.28) |
| Japan | 33674.87 (19517.38-51765.82) | 12996.64 (8768.37-18002.06) | -0.61 | 75.14 (43.55-115.51) | 40.1 (27.05-55.54) | -1.09 (-1.52--0.66) |
| Jordan | 465.06 (282.85-731.86) | 2207.97 (1443.75-3219.7) | 3.75 | 30.26 (18.4-47.62) | 41.13 (26.89-59.98) | 1.07 (0.82-1.33) |
| Kazakhstan | 8567.33 (5106-12542.22) | 9011.78 (5470.08-13264.22) | 0.05 | 126.19 (75.21-184.74) | 129.31 (78.49-190.32) | -0.08 (-0.15-0) |
| Kenya | 2402.08 (1437.2-3735) | 5975.27 (3532.58-9456.02) | 1.49 | 27.43 (16.41-42.65) | 27.6 (16.32-43.67) | 0.07 (0.02-0.11) |
| Kiribati | 4.4 (2.54-6.97) | 7.38 (4.12-11.83) | 0.68 | 14.41 (8.32-22.83) | 14.84 (8.29-23.8) | -0.04 (-0.11-0.03) |
| Kuwait | 295.7 (177.13-464.23) | 790.71 (470.44-1261.59) | 1.67 | 34.99 (20.96-54.93) | 37.25 (22.16-59.43) | 0.13 (0.1-0.16) |
| Kyrgyzstan | 1934.08 (1155.28-2886.43) | 3201.81 (1963.06-4778.58) | 0.66 | 107.23 (64.05-160.04) | 117.65 (72.13-175.58) | 0.17 (0.1-0.24) |
| Lao People's Democratic Republic | 237.31 (141.62-378.43) | 586.11 (338.45-904.9) | 1.47 | 15.36 (9.17-24.49) | 18.27 (10.55-28.21) | 0.55 (0.44-0.65) |
| Latvia | 2725.87 (2108.99-3552.62) | 2139.78 (1750.84-2634.36) | -0.22 | 285.74 (221.08-372.4) | 397.28 (325.07-489.1) | 1.09 (0.83-1.35) |
| Lebanon | 392.66 (241.8-609.05) | 850.45 (493.29-1343.55) | 1.17 | 34.06 (20.98-52.83) | 36.65 (21.26-57.9) | 0.19 (0.12-0.26) |
| Lesotho | 93.48 (53.41-144.75) | 154.04 (87.7-240.06) | 0.65 | 17.33 (9.9-26.83) | 18.52 (10.54-28.86) | 0.27 (0.23-0.31) |
| Liberia | 177.09 (100.25-273.5) | 421.25 (243.12-658.89) | 1.38 | 19.19 (10.87-29.64) | 18.76 (10.83-29.35) | 0.01 (-0.06-0.09) |
| Libya | 511.76 (304.56-786.59) | 1027.3 (598.2-1600.19) | 1.01 | 30.46 (18.13-46.82) | 34.24 (19.94-53.33) | 0.57 (0.5-0.63) |
| Lithuania | 2936.13 (2324.86-3673.78) | 1891.73 (1506.61-2326.2) | -0.36 | 210.73 (166.86-263.67) | 234.97 (187.13-288.94) | 0.54 (0.33-0.77) |
| Luxembourg | 42.45 (28.66-59.72) | 44.55 (32.77-59.62) | 0.05 | 28.76 (19.42-40.47) | 20.2 (14.85-27.02) | -0.88 (-0.99--0.78) |
| Madagascar | 770.98 (435.23-1220.21) | 1977.78 (1141.43-3001.59) | 1.57 | 17.02 (9.61-26.94) | 16.89 (9.75-25.63) | 0.01 (-0.02-0.03) |
| Malawi | 627.16 (351.57-984.55) | 1401.19 (786.35-2199.53) | 1.23 | 16.78 (9.41-26.35) | 17.12 (9.61-26.88) | 0.14 (0.11-0.17) |
| Malaysia | 1136.88 (644.82-1776.46) | 2196.6 (1283.46-3513.25) | 0.93 | 15.32 (8.69-23.94) | 15.8 (9.23-25.27) | 0.03 (-0.04-0.1) |
| Maldives | 10.8 (6.19-17.32) | 47.41 (26.75-76.96) | 3.39 | 13.29 (7.61-21.31) | 18.22 (10.28-29.58) | 0.88 (0.73-1.02) |
| Mali | 498.66 (287.74-766.33) | 1431.29 (836.06-2207.59) | 1.87 | 16.7 (9.64-25.67) | 16.07 (9.39-24.78) | -0.08 (-0.11--0.05) |
| Malta | 12.71 (7.4-21.02) | 11.99 (7.76-18.13) | -0.06 | 9.21 (5.36-15.23) | 8.96 (5.8-13.55) | 0.14 (-0.05-0.33) |
| Marshall Islands | 2.49 (1.4-3.93) | 3.7 (2.07-6.04) | 0.49 | 14.52 (8.13-22.93) | 15.61 (8.74-25.47) | 0.35 (0.3-0.4) |
| Mauritania | 127.58 (73.2-198.3) | 274.47 (159.31-422.4) | 1.15 | 16.59 (9.52-25.79) | 16.08 (9.33-24.74) | -0.05 (-0.08--0.02) |
| Mauritius | 82.45 (48.61-130.75) | 75.47 (42.39-120.62) | -0.08 | 16.58 (9.78-26.3) | 16.58 (9.31-26.5) | -0.07 (-0.13--0.02) |
| Mexico | 6758.18 (4775.33-9370.57) | 10280.86 (7652.52-13617.08) | 0.52 | 18.95 (13.39-26.28) | 19.96 (14.86-26.43) | 0.22 (0.11-0.34) |
| Micronesia (Federated States of) | 6.27 (3.64-10.07) | 6.31 (3.57-9.81) | 0.01 | 15.66 (9.1-25.12) | 14.86 (8.41-23.11) | -0.18 (-0.23--0.14) |
| Monaco | 1.64 (0.96-2.65) | 1.63 (0.93-2.66) | -0.01 | 17.93 (10.5-28.94) | 17.49 (10.02-28.56) | -0.33 (-0.48--0.18) |
| Mongolia | 883.85 (534.49-1326.49) | 1687.17 (1034.1-2481.17) | 0.91 | 100.02 (60.49-150.11) | 133.68 (81.93-196.59) | 0.98 (0.95-1.01) |
| Montenegro | 136.12 (78.35-207.53) | 119.52 (69.14-185.88) | -0.12 | 54.23 (31.21-82.67) | 58.1 (33.61-90.35) | 0.23 (0.15-0.31) |
| Morocco | 3321.65 (2005.3-5145.46) | 4828.03 (2870.21-7517.21) | 0.45 | 31.95 (19.29-49.49) | 32.89 (19.55-51.2) | 0.15 (0.11-0.18) |
| Mozambique | 780.51 (457.67-1209.95) | 1992.88 (1102.46-3101.29) | 1.55 | 16.46 (9.65-25.51) | 16.57 (9.17-25.79) | 0.04 (0.02-0.07) |
| Myanmar | 2409.49 (1367.68-3842.56) | 3622.3 (2078.29-5791.8) | 0.5 | 14.04 (7.97-22.38) | 16.11 (9.24-25.76) | 0.48 (0.46-0.5) |
| Namibia | 97.54 (52.74-152.61) | 220.34 (124.56-350.25) | 1.26 | 17.44 (9.43-27.29) | 21.08 (11.92-33.51) | 0.71 (0.67-0.75) |
| Nauru | 0.64 (0.35-1.01) | 0.76 (0.45-1.2) | 0.19 | 15.82 (8.72-25.05) | 16.32 (9.64-25.76) | 0.12 (0.07-0.16) |
| Nepal | 1279.1 (721.6-1973.58) | 2422.62 (1388.11-3728.44) | 0.89 | 17.51 (9.88-27.02) | 18.07 (10.35-27.81) | 0.11 (0.09-0.14) |
| Netherlands | 1119.27 (628.91-1827.64) | 948.89 (539.64-1519.11) | -0.15 | 18.56 (10.43-30.31) | 17.97 (10.22-28.77) | -0.37 (-0.52--0.23) |
| New Zealand | 265.11 (182.42-388.43) | 343.6 (257.4-459.34) | 0.3 | 19.19 (13.2-28.12) | 19.07 (14.29-25.5) | -0.07 (-0.16-0.03) |
| Nicaragua | 316.16 (195.38-467.78) | 709.76 (427.9-1091.19) | 1.24 | 21.42 (13.24-31.69) | 24.94 (15.03-38.34) | 0.48 (0.43-0.52) |
| Niger | 461.9 (263.91-721.34) | 1410.98 (822.8-2168.63) | 2.05 | 16.6 (9.48-25.92) | 15.82 (9.23-24.32) | -0.13 (-0.15--0.11) |
| Nigeria | 9628.92 (5747.52-15182.46) | 26428.36 (15989.46-40729.37) | 1.74 | 28.2 (16.83-44.47) | 29.38 (17.78-45.29) | 0.16 (0.14-0.19) |
| Niue | 0.13 (0.07-0.21) | 0.1 (0.05-0.15) | -0.23 | 15.85 (9-25.68) | 16.75 (9.61-26.31) | 0.17 (0.12-0.22) |
| North Macedonia | 455.4 (264.44-697.56) | 463.13 (259.86-724.65) | 0.02 | 57.35 (33.3-87.84) | 60.56 (33.98-94.75) | 0.12 (-0.05-0.29) |
| Northern Mariana Islands | 3.84 (2.18-6.2) | 2.7 (1.57-4.37) | -0.3 | 16.41 (9.29-26.46) | 16.38 (9.53-26.48) | -0.17 (-0.3--0.04) |
| Norway | 949.43 (548.91-1463.79) | 693.78 (426.05-1044.71) | -0.27 | 59.35 (34.31-91.5) | 39.09 (24.01-58.86) | -0.51 (-0.83--0.19) |
| Oman | 290.34 (175.29-455.51) | 888.56 (530.55-1397.14) | 2.06 | 34.99 (21.13-54.9) | 38.39 (22.92-60.37) | 0.34 (0.2-0.48) |
| Pakistan | 10703.33 (6407.8-16482.4) | 26640.73 (15913.9-41447.81) | 1.49 | 26.23 (15.7-40.39) | 26.94 (16.09-41.91) | 0.08 (0.07-0.1) |
| Palau | 1.08 (0.63-1.73) | 1.04 (0.6-1.76) | -0.04 | 15.55 (8.97-24.79) | 17.73 (10.16-29.91) | 0.32 (0.24-0.41) |
| Palestine | 246.15 (145.89-382.05) | 733.71 (440.91-1165.64) | 1.98 | 32.07 (19.01-49.78) | 33.6 (20.19-53.38) | 0.07 (0.01-0.13) |
| Panama | 246.25 (154.69-374.21) | 442.46 (270.8-652.47) | 0.8 | 24.34 (15.29-36.99) | 26.81 (16.41-39.53) | 0.29 (0.28-0.31) |
| Papua New Guinea | 242.18 (138.41-392.56) | 630.86 (359.6-988.36) | 1.6 | 14.63 (8.36-23.72) | 14.74 (8.4-23.1) | 0.04 (0.01-0.07) |
| Paraguay | 513.34 (278.83-803.08) | 1004.86 (552.92-1580.68) | 0.96 | 32.75 (17.79-51.24) | 32.84 (18.07-51.66) | -0.18 (-0.31--0.05) |
| Peru | 1444.57 (872.88-2196.76) | 2694.92 (1581.43-4264.89) | 0.87 | 16.28 (9.84-24.76) | 18.13 (10.64-28.7) | 0.34 (0.3-0.39) |
| Philippines | 6790.72 (4021.78-10784.45) | 13627.18 (8033.13-21265.36) | 1.01 | 26.2 (15.52-41.61) | 28.84 (17-45.01) | 0.31 (0.29-0.33) |
| Poland | 13941.63 (7418.31-22275.19) | 6347.66 (3962.66-9471.47) | -0.54 | 96.52 (51.36-154.21) | 52.47 (32.75-78.29) | -2.6 (-3.25--1.94) |
| Portugal | 759.85 (450.93-1191.41) | 598.02 (336.48-956.58) | -0.21 | 20.07 (11.91-31.47) | 20.26 (11.4-32.41) | 0.08 (0-0.17) |
| Puerto Rico | 387.23 (231.91-620.33) | 290.91 (170.53-461.33) | -0.25 | 27.37 (16.39-43.85) | 28.13 (16.49-44.6) | 0.03 (0-0.07) |
| Qatar | 93.94 (55.82-144.09) | 697.19 (393.3-1096.7) | 6.42 | 39.73 (23.61-60.94) | 42.19 (23.8-66.37) | 0.25 (0.21-0.3) |
| Republic of Korea | 11298.1 (6697.37-17162.95) | 9843.67 (5724.41-14926.68) | -0.13 | 53.68 (31.82-81.54) | 61.52 (35.77-93.28) | 0.31 (0.27-0.36) |
| Republic of Moldova | 5940.61 (3567.13-9065) | 4626.82 (2736.98-7210.35) | -0.22 | 340.86 (204.68-520.14) | 373.05 (220.68-581.36) | -0.07 (-0.33-0.2) |
| Romania | 5197.23 (2964.08-8110.53) | 2306.91 (1618.06-3258.95) | -0.56 | 59.83 (34.12-93.36) | 42.81 (30.02-60.47) | 0.23 (-0.27-0.72) |
| Russian Federation | 249657.96 (151922.84-379683.35) | 219229.93 (132745.7-334000.13) | -0.12 | 429.18 (261.16-652.7) | 471.71 (285.63-718.66) | 0.21 (0.1-0.32) |
| Rwanda | 575.52 (324.14-912.63) | 1144.13 (637.67-1799.61) | 0.99 | 21 (11.83-33.3) | 20.17 (11.24-31.73) | -0.11 (-0.19--0.03) |
| Saint Kitts and Nevis | 4.3 (2.59-6.63) | 6.22 (3.58-9.77) | 0.45 | 24.87 (14.97-38.36) | 27.38 (15.76-43.01) | 0.15 (0.12-0.19) |
| Saint Lucia | 16.33 (9.38-25.35) | 21.27 (12.19-34.66) | 0.3 | 29.02 (16.67-45.05) | 32.18 (18.45-52.45) | 0.18 (0.09-0.26) |
| Saint Vincent and the Grenadines | 11.4 (6.65-17.71) | 12.41 (7.36-19.39) | 0.09 | 24.82 (14.47-38.55) | 30.03 (17.81-46.92) | 0.55 (0.52-0.57) |
| Samoa | 9.64 (5.43-15.14) | 11.95 (6.84-19.27) | 0.24 | 14.39 (8.1-22.62) | 14.88 (8.51-23.99) | 0.02 (-0.08-0.12) |
| San Marino | 1.75 (1.03-2.75) | 1.73 (1-2.76) | -0.01 | 18.69 (10.98-29.26) | 19.27 (11.12-30.8) | 0.11 (-0.08-0.3) |
| Sao Tome and Principe | 7.53 (4.28-11.6) | 17.88 (9.81-28.22) | 1.37 | 17.54 (9.98-27.02) | 19.68 (10.79-31.05) | 0.43 (0.39-0.47) |
| Saudi Arabia | 2174.83 (1331.91-3373.26) | 6756.88 (4068.32-10587.42) | 2.11 | 32.73 (20.04-50.76) | 36.48 (21.96-57.16) | 0.42 (0.37-0.46) |
| Senegal | 455.9 (262.5-692.4) | 1073.24 (620.2-1651) | 1.35 | 16.52 (9.51-25.09) | 16.64 (9.62-25.61) | 0.03 (0-0.05) |
| Serbia | 1726.84 (1000.52-2761.79) | 908.17 (637.72-1292.71) | -0.47 | 48.09 (27.86-76.91) | 30.64 (21.51-43.61) | -0.35 (-0.86-0.16) |
| Seychelles | 4.9 (2.83-7.78) | 7.08 (4.14-11.39) | 0.44 | 15.69 (9.05-24.93) | 18.45 (10.79-29.67) | 0.47 (0.36-0.58) |
| Sierra Leone | 303.99 (174.21-473.15) | 675.5 (379.19-1057.45) | 1.22 | 19.03 (10.91-29.62) | 18.11 (10.17-28.35) | -0.2 (-0.24--0.17) |
| Singapore | 596.55 (329.99-884.81) | 881.55 (497.27-1355.95) | 0.48 | 39.53 (21.86-58.63) | 45.83 (25.85-70.49) | 0.43 (0.33-0.54) |
| Slovakia | 1881.66 (1435.17-2363.8) | 1423.82 (1159.32-1702.17) | -0.24 | 91.92 (70.11-115.47) | 83.2 (67.74-99.47) | 0.02 (-0.32-0.36) |
| Slovenia | 336.79 (243.75-446.49) | 168.55 (133.11-210.11) | -0.5 | 43.95 (31.81-58.26) | 29.6 (23.38-36.9) | -0.56 (-0.95--0.16) |
| Solomon Islands | 17.51 (10.13-27.56) | 39.46 (22.8-62.44) | 1.25 | 13.65 (7.9-21.49) | 14.43 (8.33-22.83) | 0.31 (0.25-0.36) |
| Somalia | 479.34 (279.18-737.6) | 1315.66 (768.74-2034.77) | 1.74 | 16.53 (9.63-25.44) | 15.87 (9.27-24.54) | 0.09 (0-0.19) |
| South Africa | 5394.36 (3226.34-8448.39) | 8535.9 (5102.27-13267.9) | 0.58 | 34.28 (20.5-53.69) | 35.2 (21.04-54.72) | -0.04 (-0.13-0.06) |
| South Sudan | 371.93 (212.62-577.47) | 567.13 (329.3-864.84) | 0.52 | 16.11 (9.21-25.02) | 15.76 (9.15-24.03) | -0.02 (-0.06-0.02) |
| Spain | 1748.65 (1097.32-2773.95) | 1928.28 (1428.58-2513.91) | 0.1 | 11.79 (7.4-18.71) | 15.53 (11.51-20.25) | 1.07 (0.72-1.42) |
| Sri Lanka | 1135.31 (634.55-1831.34) | 1389.59 (825.85-2199.53) | 0.22 | 15.35 (8.58-24.77) | 17.23 (10.24-27.27) | 0.47 (0.44-0.51) |
| Sudan | 3204.44 (1952.35-4966.98) | 5670.64 (3493.98-8782.29) | 0.77 | 42.05 (25.62-65.18) | 30.68 (18.9-47.51) | -1.17 (-1.29--1.06) |
| Suriname | 41.49 (24.9-66.45) | 58.58 (34.15-96.38) | 0.41 | 25.48 (15.29-40.81) | 27.29 (15.91-44.9) | 0.12 (0.06-0.17) |
| Sweden | 1186.97 (686.8-1826.92) | 1165.09 (679.28-1797.68) | -0.02 | 40.44 (23.4-62.24) | 35.94 (20.96-55.46) | -0.32 (-0.37--0.28) |
| Switzerland | 367.69 (296.07-452.68) | 287.28 (236.31-351.38) | -0.22 | 13.95 (11.24-17.18) | 10.35 (8.51-12.66) | -0.85 (-0.92--0.79) |
| Syrian Arab Republic | 1493.09 (874.62-2348.42) | 1493.52 (901.43-2287.43) | 0 | 31.12 (18.23-48.95) | 29.36 (17.72-44.97) | 0.05 (-0.05-0.14) |
| Taiwan (Province of China) | 1132.7 (697.31-1796.36) | 931.18 (574.99-1426.27) | -0.18 | 12.28 (7.56-19.47) | 12.34 (7.62-18.9) | -0.07 (-0.17-0.02) |
| Tajikistan | 2223.16 (1365.04-3327.69) | 4611.29 (2675.69-6967.82) | 1.07 | 105.13 (64.55-157.36) | 110.55 (64.15-167.05) | 0.01 (-0.07-0.1) |
| Thailand | 4365.26 (2479.54-6880.18) | 4107.62 (2369.65-6464.04) | -0.06 | 16.83 (9.56-26.53) | 19.38 (11.18-30.49) | 0.45 (0.38-0.53) |
| Timor-Leste | 46.93 (26.14-75.29) | 82.28 (46.29-130.4) | 0.75 | 14.74 (8.21-23.66) | 14.4 (8.1-22.82) | -0.18 (-0.22--0.14) |
| Togo | 237.41 (138.23-366.59) | 595.73 (332.99-905.28) | 1.51 | 17.32 (10.08-26.74) | 17.71 (9.9-26.91) | 0.14 (0.12-0.17) |
| Tokelau | 0.09 (0.05-0.14) | 0.08 (0.04-0.13) | -0.11 | 14.91 (8.41-23.6) | 16.11 (9-25.59) | 0.27 (0.21-0.32) |
| Tonga | 5.02 (2.9-7.94) | 5.65 (3.31-8.76) | 0.13 | 13.61 (7.85-21.51) | 14.51 (8.5-22.53) | 0.19 (0.11-0.27) |
| Trinidad and Tobago | 133.73 (76.55-212.78) | 150.48 (87.71-248.72) | 0.13 | 26.67 (15.27-42.44) | 30.23 (17.62-49.97) | 0.48 (0.39-0.56) |
| Tunisia | 1120.51 (689.97-1744.55) | 1641.27 (1010.59-2589.73) | 0.46 | 32.57 (20.06-50.72) | 37.72 (23.22-59.51) | 0.49 (0.45-0.53) |
| T眉rkiye | 7852.24 (4672.08-12232.74) | 11196.3 (6551.27-17510.04) | 0.43 | 32.83 (19.53-51.14) | 35.14 (20.56-54.96) | 0.24 (0.23-0.26) |
| Turkmenistan | 1532.42 (907.03-2292.13) | 2512.49 (1532.02-3646.7) | 0.64 | 99.83 (59.09-149.32) | 120.8 (73.66-175.33) | 0.52 (0.47-0.57) |
| Tuvalu | 0.55 (0.31-0.89) | 0.75 (0.44-1.22) | 0.36 | 15.14 (8.49-24.59) | 15.19 (8.85-24.6) | -0.02 (-0.07-0.02) |
| Uganda | 1151.13 (658.11-1797.22) | 3522.21 (2036.48-5564.75) | 2.06 | 17.95 (10.26-28.02) | 20.48 (11.84-32.36) | 0.47 (0.45-0.49) |
| Ukraine | 73491.8 (45919.89-112449.35) | 60891.2 (37065.7-93733.92) | -0.17 | 386.91 (241.75-592.01) | 441.72 (268.88-679.97) | 0.58 (0.46-0.7) |
| United Arab Emirates | 402.19 (229.35-629.17) | 1896.63 (1097.52-3112.91) | 3.72 | 42.07 (23.99-65.82) | 47.21 (27.32-77.49) | 0.49 (0.34-0.64) |
| United Kingdom | 7826.72 (5079.02-11170.91) | 8073.87 (5515.57-11343.53) | 0.03 | 37.45 (24.3-53.45) | 37.12 (25.36-52.15) | -0.32 (-0.45--0.2) |
| United Republic of Tanzania | 1761.56 (958.71-2728.31) | 4415.1 (2475.48-7007.24) | 1.51 | 18.18 (9.89-28.16) | 18.92 (10.61-30.03) | 0.2 (0.17-0.22) |
| United States of America | 57219.69 (33484.81-88610.59) | 41349.64 (27792.93-59378.33) | -0.28 | 56 (32.77-86.73) | 37.15 (24.97-53.35) | -1.78 (-2.1--1.46) |
| United States Virgin Islands | 10.42 (6.04-16.62) | 7 (4.11-10.9) | -0.33 | 26.31 (15.24-41.95) | 30.28 (17.76-47.12) | 0.4 (0.37-0.43) |
| Uruguay | 273.39 (161.44-429.37) | 287.62 (166.81-449.5) | 0.05 | 24.07 (14.21-37.8) | 24.04 (13.95-37.58) | 0 (-0.03-0.02) |
| Uzbekistan | 8782.83 (5365.12-13150.45) | 16645.15 (9954.12-24694.11) | 0.9 | 102.32 (62.51-153.21) | 121.17 (72.46-179.76) | 0.34 (0.27-0.42) |
| Vanuatu | 8.6 (4.91-13.44) | 18.66 (10.64-29.96) | 1.17 | 14.68 (8.37-22.93) | 14.97 (8.54-24.04) | 0.1 (0.06-0.15) |
| Venezuela (Bolivarian Republic of) | 2191.18 (1343.98-3255.08) | 2406.4 (1502.46-3677.68) | 0.1 | 27.36 (16.78-40.64) | 25.7 (16.05-39.28) | -0.19 (-0.22--0.16) |
| Viet Nam | 4132.43 (2356.84-6520.98) | 7799.64 (4497.13-12389.75) | 0.89 | 14.49 (8.26-22.87) | 20.32 (11.71-32.27) | 1.13 (1.02-1.23) |
| Yemen | 1498.62 (901.69-2315.72) | 4312.51 (2560.24-6815.16) | 1.88 | 32.6 (19.62-50.38) | 31.34 (18.61-49.53) | 0.02 (-0.06-0.1) |
| Zambia | 525.39 (295.32-819.33) | 1494.17 (832.9-2366.28) | 1.84 | 17.32 (9.73-27) | 18.46 (10.29-29.24) | 0.27 (0.23-0.31) |
| Zimbabwe | 689.8 (391.42-1055.82) | 1130.44 (637.36-1763.83) | 0.64 | 17.4 (9.87-26.63) | 17.84 (10.06-27.83) | 0.19 (0.13-0.25) |
