# Supplementary material for: Global burden of pancreatitis among individuals aged 15–39 years: a systematic analysis from the 2021 GBD study
Source: Front Med (Lausanne). 2025 May 27;12:1572346. doi: 10.3389/fmed.2025.1572346 (PMC12150401; doi:10.3389/fmed.2025.1572346)
Supplement: Supplementary file 5 [file Supplementary_file_5.docx]

**Supplementary Table 5** The incidence of pancreatitis cases and rates among aged 15-39 years in 1990 and 2021 across 204 countries, and the trends from 1990 to 2021.

| **location** | **Incidence cases** | | | **Incidence rates** | | |
| --- | --- | --- | --- | --- | --- | --- |
|  | **1990 thousand**  **(95%UI)** | **2021 thousand**  **(95%UI)** | **percentage**  **Change**  **(100%)** | **1990 per**  **(95%)** | **2021 per**  **(95%UI)** | **EAPC**  **(95% CI)** |
| Afghanistan | 245.73 (130.81-558.79) | 1126.33 (614.1-1971.58) | 3.58 | 7.8 (4.15-17.74) | 9.22 (5.02-16.13) | 0.83 (0.56-1.09) |
| Albania | 324.81 (234.4-440.43) | 169.97 (115.31-241.68) | -0.48 | 22.87 (16.5-31.01) | 17.93 (12.16-25.49) | -1.04 (-1.27--0.81) |
| Algeria | 613.36 (371.05-1037.06) | 1213.03 (808.94-1801.27) | 0.98 | 6.07 (3.67-10.26) | 7.12 (4.75-10.58) | 0.68 (0.62-0.73) |
| American Samoa | 6.77 (4.18-12.14) | 7.81 (5.26-10.78) | 0.15 | 33.48 (20.65-59.99) | 44.67 (30.08-61.7) | 1.31 (0.72-1.9) |
| Andorra | 3.53 (2.38-4.91) | 2.63 (1.75-3.71) | -0.25 | 14.11 (9.51-19.62) | 10.31 (6.85-14.53) | -0.59 (-0.89--0.29) |
| Angola | 822.76 (448.65-1415.07) | 2681.54 (1592.28-4363) | 2.26 | 21.03 (11.47-36.17) | 22.04 (13.09-35.86) | 0.48 (0.26-0.7) |
| Antigua and Barbuda | 5.57 (4.77-6.57) | 5.23 (3.99-7.32) | -0.06 | 21.6 (18.48-25.48) | 15.19 (11.59-21.29) | -0.26 (-0.67-0.14) |
| Argentina | 5887.69 (5295.07-6468.66) | 5317.86 (4793.19-5877.03) | -0.1 | 48.2 (43.35-52.96) | 30.35 (27.35-33.54) | -1.12 (-1.52--0.71) |
| Armenia | 258.7 (185.64-352.02) | 214.86 (163.41-288.62) | -0.17 | 18 (12.92-24.49) | 19.99 (15.2-26.85) | 0.13 (-0.3-0.56) |
| Australia | 780.82 (675.72-921.72) | 744.37 (608.63-905.16) | -0.05 | 11.53 (9.98-13.61) | 8.59 (7.02-10.44) | -0.94 (-1.17--0.72) |
| Austria | 758.46 (667.91-855.2) | 313.81 (271.04-366.6) | -0.59 | 25.27 (22.25-28.49) | 11.12 (9.6-12.99) | -2.85 (-3.02--2.67) |
| Azerbaijan | 976.22 (632.15-1347.39) | 1327.43 (772.07-2082.47) | 0.36 | 30.72 (19.89-42.4) | 31.34 (18.23-49.16) | -0.26 (-0.55-0.04) |
| Bahamas | 69.32 (59.71-80) | 86.5 (69.04-108.48) | 0.25 | 58.74 (50.59-67.79) | 55.93 (44.64-70.14) | -0.23 (-0.41--0.06) |
| Bahrain | 31.68 (23.41-42.47) | 78.82 (56.45-104) | 1.49 | 12.36 (9.13-16.57) | 11.2 (8.02-14.78) | -0.25 (-0.38--0.13) |
| Bangladesh | 26179.74 (18828.16-35473.76) | 33524.75 (20299.62-50275.82) | 0.28 | 62.02 (44.61-84.04) | 48.72 (29.5-73.06) | -0.78 (-0.89--0.67) |
| Barbados | 36.93 (32.29-42.36) | 24.15 (19.06-30.79) | -0.35 | 33.83 (29.59-38.81) | 24.45 (19.3-31.17) | -1.35 (-1.6--1.1) |
| Belarus | 5196.36 (4280.93-6146.15) | 5362.4 (4304.96-6652.75) | 0.03 | 131.74 (108.53-155.82) | 182.79 (146.74-226.77) | 0.42 (-0.1-0.93) |
| Belgium | 761.64 (668.63-876.01) | 419.93 (347.39-517.19) | -0.45 | 20.47 (17.97-23.54) | 11.99 (9.92-14.77) | -2.1 (-2.32--1.88) |
| Belize | 12.97 (9.56-18.05) | 43.23 (37.6-49.06) | 2.33 | 17.73 (13.07-24.67) | 22.9 (19.92-25.99) | 1.02 (0.53-1.52) |
| Benin | 897.53 (527.96-1544.52) | 2920.51 (1807.29-4657.81) | 2.25 | 52.71 (31.01-90.7) | 55.71 (34.47-88.85) | 0.12 (0.03-0.21) |
| Bermuda | 8.31 (7.16-9.45) | 3.31 (2.68-4.14) | -0.6 | 32.32 (27.85-36.75) | 18.91 (15.3-23.65) | -1.69 (-1.95--1.42) |
| Bhutan | 211.76 (100.28-368.28) | 240.3 (127.6-441.49) | 0.13 | 78.59 (37.22-136.67) | 69.33 (36.81-127.38) | -0.51 (-0.56--0.46) |
| Bolivia (Plurinational State of) | 1974.13 (1234.44-2874.28) | 2914.28 (1880.47-4202.39) | 0.48 | 80.05 (50.06-116.55) | 59.31 (38.27-85.52) | -1.26 (-1.41--1.11) |
| Bosnia and Herzegovina | 993.65 (751.1-1344.64) | 351.19 (232.78-487.56) | -0.65 | 52.32 (39.55-70.8) | 34.9 (23.13-48.45) | -1.53 (-1.73--1.33) |
| Botswana | 182.23 (68.73-450.27) | 289.66 (136.8-568.05) | 0.59 | 35.4 (13.35-87.47) | 27.19 (12.84-53.32) | -0.91 (-1.1--0.72) |
| Brazil | 37941.11 (36034.3-39933.7) | 47348.41 (44274.03-50364.99) | 0.25 | 60.47 (57.43-63.64) | 55.54 (51.93-59.08) | -0.14 (-0.38-0.11) |
| Brunei Darussalam | 24.21 (17.23-32.19) | 35.48 (26.68-47.46) | 0.47 | 19.63 (13.97-26.11) | 17.39 (13.08-23.26) | -0.47 (-0.71--0.23) |
| Bulgaria | 1468.52 (1312.45-1646.34) | 1049.66 (845.28-1289.69) | -0.29 | 49.33 (44.09-55.3) | 55.26 (44.5-67.89) | 0.41 (0.19-0.62) |
| Burkina Faso | 1068.13 (629.4-1704.87) | 3779.1 (2272.97-6019.02) | 2.54 | 33.5 (19.74-53.48) | 43.67 (26.26-69.55) | 0.95 (0.75-1.16) |
| Burundi | 457.64 (234.05-942.75) | 1118.39 (521.92-2061.3) | 1.44 | 22.08 (11.29-45.48) | 21.21 (9.9-39.1) | -0.35 (-0.57--0.13) |
| Cabo Verde | 54.89 (31.28-91.01) | 127.28 (80.65-197.14) | 1.32 | 41.98 (23.92-69.6) | 50.79 (32.18-78.66) | 0.26 (0.05-0.47) |
| Cambodia | 2045.13 (1256.28-3701.93) | 4010.51 (2313.17-6916.87) | 0.96 | 53.12 (32.63-96.15) | 55.37 (31.94-95.5) | -0.05 (-0.14-0.03) |
| Cameroon | 1951.25 (1106.68-3193.95) | 7818.59 (4357.36-13876.54) | 3.01 | 51.3 (29.1-83.98) | 60.64 (33.8-107.63) | 0.64 (0.5-0.78) |
| Canada | 1846.79 (1619.81-2152.71) | 2236.82 (1926.75-2618.99) | 0.21 | 16.61 (14.57-19.36) | 18.86 (16.24-22.08) | 0.04 (-0.21-0.29) |
| Central African Republic | 277.67 (159.56-455.13) | 565.97 (294.94-929.39) | 1.04 | 26.66 (15.32-43.71) | 25.92 (13.51-42.57) | -0.09 (-0.19-0) |
| Chad | 802.39 (358.3-1603.47) | 3062.99 (1635.58-5478.68) | 2.82 | 38.23 (17.07-76.39) | 48.67 (25.99-87.06) | 1 (0.87-1.13) |
| Chile | 2200.44 (2028.32-2383.13) | 1743.25 (1543.32-1932.46) | -0.21 | 38.42 (35.41-41.61) | 24.64 (21.81-27.31) | -1.36 (-1.62--1.1) |
| China | 100332.99 (81993.65-123708.13) | 59204.17 (45558.86-77415.93) | -0.41 | 18.3 (14.96-22.57) | 12.83 (9.87-16.78) | -1.42 (-1.58--1.27) |
| Colombia | 4530.37 (4192.34-4940.31) | 4692.84 (3992.52-5477.26) | 0.04 | 32.23 (29.83-35.15) | 23.35 (19.87-27.26) | -0.95 (-1.22--0.68) |
| Comoros | 38.19 (19.47-75.53) | 76.43 (41.37-120.92) | 1 | 22.11 (11.27-43.72) | 24.67 (13.35-39.03) | -0.17 (-0.68-0.33) |
| Congo | 231.93 (111.97-444.09) | 623.55 (366.77-1026.38) | 1.69 | 24.48 (11.82-46.88) | 28.14 (16.55-46.32) | 0.66 (0.48-0.85) |
| Cook Islands | 6 (3.84-9.18) | 3.37 (2.22-4.95) | -0.44 | 77.76 (49.71-119) | 57.16 (37.67-84.05) | -0.83 (-0.98--0.68) |
| Costa Rica | 594.89 (531.41-661.74) | 1007.29 (902.46-1125) | 0.69 | 46.31 (41.37-51.51) | 52.92 (47.42-59.11) | 0.01 (-0.28-0.3) |
| Côte d'Ivoire | 2529.5 (1681.37-3830.91) | 7017.02 (4645.51-10158.06) | 1.77 | 53.48 (35.55-80.99) | 62.59 (41.43-90.6) | 0.59 (0.42-0.76) |
| Croatia | 1055.68 (948.25-1188.57) | 321.53 (260.75-386.45) | -0.7 | 58.18 (52.26-65.5) | 25.76 (20.89-30.96) | -2.82 (-3.01--2.64) |
| Cuba | 1159.8 (994.71-1319.94) | 792.79 (678.46-924.43) | -0.32 | 23.77 (20.38-27.05) | 22.11 (18.92-25.78) | -0.67 (-0.97--0.36) |
| Cyprus | 60.19 (44.87-81.21) | 61.43 (44.69-88.27) | 0.02 | 19.57 (14.59-26.41) | 12.24 (8.91-17.59) | -1.87 (-2.1--1.65) |
| Czechia | 2739.04 (2392.77-3109.06) | 1338.19 (1097.32-1618.51) | -0.51 | 73.82 (64.48-83.79) | 45.32 (37.16-54.81) | -1.36 (-1.68--1.03) |
| Democratic People's Republic of Korea | 1531.75 (836.96-2543.47) | 1721.34 (763.88-3479.15) | 0.12 | 18.36 (10.03-30.49) | 17.11 (7.59-34.59) | -0.47 (-0.55--0.38) |
| Democratic Republic of the Congo | 3652.28 (2039.44-6687.4) | 9548.47 (5305.71-16888.54) | 1.61 | 25.5 (14.24-46.68) | 26.46 (14.7-46.8) | 0.26 (0.11-0.41) |
| Denmark | 475.68 (418.93-536.67) | 224.92 (197.38-257.63) | -0.53 | 24.93 (21.96-28.13) | 12.33 (10.82-14.13) | -2.74 (-3.12--2.36) |
| Djibouti | 38.78 (16.06-84.27) | 140.78 (66.64-249.12) | 2.63 | 22.12 (9.16-48.06) | 26.03 (12.32-46.07) | 0.37 (0.2-0.55) |
| Dominica | 7.5 (5.43-9.89) | 7.99 (5.74-10.38) | 0.07 | 25.68 (18.6-33.88) | 30.84 (22.15-40.09) | 0.74 (0.53-0.94) |
| Dominican Republic | 690.47 (513.73-882.57) | 930.9 (567.7-1342.01) | 0.35 | 22.48 (16.72-28.73) | 20.47 (12.48-29.51) | 0.16 (-0.12-0.44) |
| Ecuador | 3446.16 (3095.14-3818.69) | 3054.78 (2495.58-3755.25) | -0.11 | 83.52 (75.01-92.54) | 41.82 (34.17-51.41) | -2 (-2.29--1.7) |
| Egypt | 1245.98 (862.83-1757.93) | 2225.29 (1586.53-3079.93) | 0.79 | 5.68 (3.94-8.02) | 5.27 (3.76-7.3) | -0.86 (-1.19--0.54) |
| El Salvador | 1310.22 (951.56-1620.48) | 1206.54 (830.54-1739.05) | -0.08 | 62.71 (45.54-77.56) | 46.55 (32.04-67.09) | -0.81 (-1--0.63) |
| Equatorial Guinea | 49.21 (23.51-120.85) | 184.11 (94.87-357.89) | 2.74 | 32.59 (15.57-80.03) | 26.47 (13.64-51.45) | -0.69 (-0.94--0.45) |
| Eritrea | 305.93 (169.66-491.94) | 734.24 (411-1303.11) | 1.4 | 23.61 (13.09-37.96) | 26.22 (14.68-46.54) | 0.28 (0.19-0.36) |
| Estonia | 571.13 (452.89-737.1) | 340.49 (261.5-433.57) | -0.4 | 100.55 (79.74-129.77) | 86.1 (66.13-109.64) | -0.48 (-0.81--0.15) |
| Eswatini | 86.89 (47.31-178.49) | 185.37 (102.55-291.28) | 1.13 | 28.83 (15.7-59.23) | 36.41 (20.14-57.21) | 0.77 (0.56-0.99) |
| Ethiopia | 3014.52 (2028.02-4749.44) | 8291.76 (4125.13-13688.23) | 1.75 | 16.5 (11.1-26) | 17.89 (8.9-29.53) | 0.16 (0.1-0.22) |
| Fiji | 92.28 (57.92-129.38) | 67.65 (46.37-100.09) | -0.27 | 28.62 (17.96-40.12) | 18.96 (13-28.06) | -1.63 (-1.8--1.46) |
| Finland | 868.45 (711.58-1036.3) | 393.73 (341.56-446.89) | -0.55 | 47.84 (39.2-57.08) | 23.63 (20.5-26.82) | -1.93 (-2.09--1.76) |
| France | 4650.3 (4180.55-5172.78) | 2314.28 (1994.91-2667.56) | -0.5 | 21.14 (19-23.51) | 11.65 (10.04-13.43) | -1.71 (-1.91--1.5) |
| Gabon | 79.12 (49.86-125.83) | 175.06 (103-288.8) | 1.21 | 20.57 (12.96-32.71) | 23.36 (13.74-38.53) | 0.35 (0.24-0.46) |
| Gambia | 244.42 (142.91-436.39) | 831.17 (499.67-1371.69) | 2.4 | 64.83 (37.9-115.74) | 83.11 (49.96-137.15) | 0.5 (0.22-0.78) |
| Georgia | 205.99 (117.11-339.83) | 389.39 (266.19-532.43) | 0.89 | 9.68 (5.5-15.96) | 34.32 (23.46-46.92) | 4.76 (4.3-5.23) |
| Germany | 9385.49 (8241.76-10561.08) | 3619.76 (3119.71-4141.09) | -0.61 | 31.59 (27.74-35.55) | 14.31 (12.33-16.37) | -3.22 (-3.53--2.91) |
| Ghana | 2161.98 (1404.14-3211.56) | 6671.08 (4216.21-10592.98) | 2.09 | 37.66 (24.46-55.95) | 46.65 (29.48-74.07) | 0.6 (0.5-0.7) |
| Greece | 656.3 (585.58-739.24) | 451.59 (388.95-523.17) | -0.31 | 17.46 (15.58-19.66) | 16.22 (13.97-18.79) | 0 (-0.27-0.27) |
| Greenland | 11.69 (8-16.55) | 5.78 (3.83-8.84) | -0.51 | 44.15 (30.22-62.52) | 28.35 (18.78-43.36) | -2.39 (-2.8--1.98) |
| Grenada | 12.52 (10.22-15.06) | 10.45 (8.66-12.56) | -0.17 | 37.55 (30.65-45.17) | 25.86 (21.43-31.07) | -1.25 (-1.45--1.05) |
| Guam | 6.81 (4.59-9.81) | 5.01 (3.51-6.8) | -0.26 | 10.73 (7.24-15.46) | 9.04 (6.33-12.26) | 0.27 (-0.23-0.77) |
| Guatemala | 4351.47 (3861.71-4839.3) | 7329.05 (5930.49-8618.88) | 0.68 | 147.31 (130.73-163.82) | 107.67 (87.13-126.62) | -0.87 (-1.18--0.55) |
| Guinea | 954.9 (457.13-1828.74) | 2541.44 (1518.84-4068.4) | 1.66 | 46.48 (22.25-89.01) | 49.19 (29.39-78.74) | 0.02 (-0.04-0.09) |
| Guinea-Bissau | 249.59 (149.99-387.87) | 670.81 (397.19-1083.75) | 1.69 | 67.31 (40.45-104.59) | 79.5 (47.07-128.44) | 0.75 (0.66-0.83) |
| Guyana | 107.72 (90.28-126.7) | 190.69 (142.79-242.69) | 0.77 | 31.65 (26.52-37.22) | 61.37 (45.95-78.1) | 2.19 (1.42-2.97) |
| Haiti | 1108.86 (677.47-1681.38) | 2215.76 (1269.04-3327.69) | 1 | 45.55 (27.83-69.07) | 40.36 (23.12-60.62) | -0.12 (-0.29-0.05) |
| Honduras | 1523.47 (1160.88-2007.68) | 2575.3 (1399.46-4332.47) | 0.69 | 88.24 (67.24-116.29) | 58.59 (31.84-98.56) | -1.59 (-1.71--1.48) |
| Hungary | 5014.06 (4504.92-5569.14) | 1167.29 (986.52-1380.42) | -0.77 | 135.66 (121.89-150.68) | 42.42 (35.85-50.17) | -4.15 (-4.68--3.61) |
| Iceland | 17.56 (15.89-19.38) | 17.74 (15.53-19.93) | 0.01 | 16.9 (15.3-18.65) | 14.82 (12.98-16.65) | -0.13 (-0.44-0.18) |
| India | 210943.7 (162596.95-324313.19) | 258254.47 (198767.74-326457.07) | 0.22 | 61.86 (47.68-95.1) | 42.37 (32.61-53.57) | -1.19 (-1.43--0.95) |
| Indonesia | 30097.87 (21483.74-46846.19) | 39680.05 (26780.44-63457.89) | 0.32 | 38.57 (27.53-60.03) | 34.85 (23.52-55.73) | -0.27 (-0.35--0.2) |
| Iran (Islamic Republic of) | 1196.57 (817.2-1774.57) | 2252.28 (1566.18-3285.83) | 0.88 | 5.51 (3.76-8.17) | 6.49 (4.51-9.47) | 1.05 (0.8-1.3) |
| Iraq | 594.7 (417.24-793) | 1083.82 (740.82-1575) | 0.82 | 8.28 (5.81-11.04) | 6.22 (4.25-9.03) | -0.79 (-0.96--0.62) |
| Ireland | 153.4 (135.33-173.97) | 128.26 (109.55-153.54) | -0.16 | 11.18 (9.87-12.68) | 8.2 (7-9.81) | -0.94 (-1.43--0.45) |
| Israel | 253.25 (222.7-291.45) | 285.19 (247.55-328.95) | 0.13 | 13.25 (11.65-15.25) | 8.58 (7.45-9.9) | -1.27 (-1.52--1.01) |
| Italy | 3170.56 (2982.87-3414.89) | 1117.91 (1033.23-1219.6) | -0.65 | 14.85 (13.97-16) | 7.08 (6.54-7.72) | -2.35 (-2.54--2.16) |
| Jamaica | 105.49 (79.28-149.24) | 136.19 (92.47-181.12) | 0.29 | 10.73 (8.07-15.19) | 11.41 (7.75-15.18) | -0.68 (-1.16--0.2) |
| Japan | 6944.32 (5769.45-8871.93) | 2950.14 (2476.13-3631.44) | -0.58 | 15.5 (12.87-19.8) | 9.1 (7.64-11.2) | -1.64 (-1.79--1.48) |
| Jordan | 164.56 (118.88-217.24) | 464.83 (344.15-632.03) | 1.82 | 10.71 (7.73-14.13) | 8.66 (6.41-11.77) | -0.73 (-0.95--0.51) |
| Kazakhstan | 8672.56 (7162.72-10115.39) | 9252.6 (7056.81-12493.35) | 0.07 | 127.74 (105.51-149) | 132.76 (101.25-179.26) | -0.72 (-1.32--0.12) |
| Kenya | 1724.71 (784.31-3471.58) | 4772.21 (2777.35-7689.75) | 1.77 | 19.69 (8.96-39.64) | 22.04 (12.83-35.52) | 0.52 (0.43-0.61) |
| Kiribati | 26.79 (15.7-40.24) | 39.17 (22.23-59.45) | 0.46 | 87.74 (51.42-131.8) | 78.78 (44.72-119.56) | -0.39 (-0.44--0.34) |
| Kuwait | 95.91 (80.26-116.8) | 202.65 (155.92-262.02) | 1.11 | 11.35 (9.5-13.82) | 9.55 (7.35-12.34) | -0.26 (-0.86-0.35) |
| Kyrgyzstan | 511.28 (419.41-637.59) | 1613.43 (1266.87-2082.24) | 2.16 | 28.35 (23.25-35.35) | 59.28 (46.55-76.51) | 2.45 (2.01-2.88) |
| Lao People's Democratic Republic | 509.03 (311.9-811.8) | 999.97 (595.66-1608.33) | 0.96 | 32.95 (20.19-52.54) | 31.17 (18.57-50.14) | -0.38 (-0.47--0.28) |
| Latvia | 1091.17 (939.1-1287.89) | 831.15 (683.53-989.11) | -0.24 | 114.38 (98.44-135) | 154.31 (126.91-183.64) | 0.47 (0.17-0.78) |
| Lebanon | 118.6 (67.36-176.75) | 197.52 (145.74-260.36) | 0.67 | 10.29 (5.84-15.33) | 8.51 (6.28-11.22) | -0.61 (-0.79--0.42) |
| Lesotho | 130.35 (46.68-325.36) | 259.42 (166.22-374.13) | 0.99 | 24.16 (8.65-60.3) | 31.19 (19.98-44.98) | 1.21 (1.01-1.4) |
| Liberia | 476.49 (298.72-755.36) | 1306.9 (773.76-2004.03) | 1.74 | 51.65 (32.38-81.87) | 58.21 (34.46-89.26) | 1.03 (0.69-1.38) |
| Libya | 121.84 (71.18-199.92) | 303.28 (210.97-438.7) | 1.49 | 7.25 (4.24-11.9) | 10.11 (7.03-14.62) | 1.69 (1.38-2) |
| Lithuania | 1259.85 (1033.39-1599.67) | 1172.41 (967.12-1378.79) | -0.07 | 90.42 (74.17-114.81) | 145.62 (120.13-171.26) | 2 (1.47-2.53) |
| Luxembourg | 29.03 (25.47-33.12) | 15.97 (13.24-19.97) | -0.45 | 19.67 (17.26-22.44) | 7.24 (6-9.05) | -3.67 (-3.91--3.44) |
| Madagascar | 872.67 (478.77-1363.83) | 2597.19 (1211.41-4563.19) | 1.98 | 19.27 (10.57-30.11) | 22.18 (10.34-38.97) | 0.35 (0.17-0.53) |
| Malawi | 845.39 (458.76-1401.8) | 2048.14 (1295.83-3231.98) | 1.42 | 22.63 (12.28-37.52) | 25.03 (15.84-39.5) | 0.37 (0.23-0.51) |
| Malaysia | 2503.06 (1835.62-3476.27) | 4572.87 (3159.97-6927.95) | 0.83 | 33.73 (24.73-46.84) | 32.89 (22.73-49.83) | -0.84 (-1.1--0.58) |
| Maldives | 9.42 (5.28-15.68) | 36.37 (23.13-64.7) | 2.86 | 11.59 (6.5-19.3) | 13.98 (8.89-24.87) | 0.78 (0.49-1.08) |
| Mali | 1733.05 (863.22-4033.69) | 5334.61 (2856.04-11063.29) | 2.08 | 58.05 (28.91-135.1) | 59.89 (32.06-124.21) | 0.26 (0.2-0.31) |
| Malta | 18.15 (16.22-20.2) | 13.66 (11.91-15.4) | -0.25 | 13.16 (11.75-14.64) | 10.2 (8.89-11.5) | -0.27 (-0.57-0.03) |
| Marshall Islands | 8.43 (5.06-12.52) | 9 (5.11-14.38) | 0.07 | 49.17 (29.49-72.98) | 37.93 (21.54-60.64) | -0.73 (-0.82--0.63) |
| Mauritania | 409.26 (206.73-688.06) | 838.01 (457-1408.46) | 1.05 | 53.23 (26.89-89.5) | 49.09 (26.77-82.51) | -0.28 (-0.35--0.22) |
| Mauritius | 583.95 (519.29-657.59) | 413.53 (360.13-468.62) | -0.29 | 117.47 (104.46-132.28) | 90.84 (79.11-102.95) | -2.16 (-2.72--1.59) |
| Mexico | 20094.76 (19315.95-21090.74) | 34762.05 (31659.55-38038.03) | 0.73 | 56.35 (54.16-59.14) | 67.48 (61.46-73.84) | 0.44 (0.16-0.72) |
| Micronesia (Federated States of) | 20.09 (12.79-30.05) | 17.36 (10.04-28.98) | -0.14 | 50.14 (31.91-75.01) | 40.87 (23.64-68.25) | -0.67 (-0.71--0.63) |
| Monaco | 1.42 (0.95-1.99) | 1.38 (0.9-2) | -0.03 | 15.47 (10.43-21.75) | 14.75 (9.69-21.48) | -0.48 (-0.75--0.21) |
| Mongolia | 749.51 (521.35-1048.5) | 1166.02 (860.29-1583.5) | 0.56 | 84.82 (59-118.65) | 92.38 (68.16-125.46) | -0.21 (-0.36--0.06) |
| Montenegro | 120.94 (91.37-157.53) | 83.3 (57.29-117.49) | -0.31 | 48.18 (36.4-62.75) | 40.49 (27.85-57.11) | -0.37 (-0.56--0.18) |
| Morocco | 708.2 (422.37-1181.71) | 1121.75 (733.33-1740.48) | 0.58 | 6.81 (4.06-11.37) | 7.64 (4.99-11.86) | 0.48 (0.32-0.65) |
| Mozambique | 1155.58 (554.99-2038.87) | 4444.44 (2613.42-7051.44) | 2.85 | 24.36 (11.7-42.99) | 36.96 (21.74-58.65) | 1.91 (1.75-2.07) |
| Myanmar | 11340.11 (6325.06-21652.4) | 13642.3 (7527.67-26019.95) | 0.2 | 66.06 (36.84-126.13) | 60.68 (33.48-115.74) | -0.44 (-0.53--0.34) |
| Namibia | 164.35 (61.63-421.03) | 319.91 (135.7-673.82) | 0.95 | 29.39 (11.02-75.29) | 30.61 (12.99-64.48) | -0.1 (-0.26-0.05) |
| Nauru | 1.85 (0.95-3.06) | 1.72 (0.74-3.14) | -0.07 | 45.74 (23.52-75.73) | 36.98 (15.94-67.46) | -0.79 (-0.97--0.61) |
| Nepal | 5575.65 (3514.28-8602.62) | 7216.25 (4795.38-10590.82) | 0.29 | 76.33 (48.11-117.76) | 53.82 (35.77-78.99) | -1.27 (-1.33--1.21) |
| Netherlands | 823.19 (734.94-923.91) | 464.33 (405.03-532.16) | -0.44 | 13.65 (12.19-15.32) | 8.79 (7.67-10.08) | -2.03 (-2.31--1.74) |
| New Zealand | 128.05 (114.35-144.78) | 120.97 (103.43-142.11) | -0.06 | 9.27 (8.28-10.48) | 6.72 (5.74-7.89) | -0.9 (-1.28--0.52) |
| Nicaragua | 594.68 (435.72-779.87) | 966.74 (696.37-1257.66) | 0.63 | 40.29 (29.52-52.84) | 33.97 (24.47-44.19) | -0.22 (-0.39--0.05) |
| Niger | 1407.05 (607.63-3247.31) | 4821.63 (1893.33-11264.21) | 2.43 | 50.55 (21.83-116.67) | 54.07 (21.23-126.32) | 0.05 (-0.07-0.16) |
| Nigeria | 14034.91 (8068.53-24131.94) | 37834.76 (22621.79-57382.52) | 1.7 | 41.1 (23.63-70.68) | 42.07 (25.15-63.8) | 0.23 (0.12-0.33) |
| Niue | 0.31 (0.19-0.48) | 0.21 (0.15-0.29) | -0.32 | 37.98 (23.12-60.24) | 36.57 (26.34-51.17) | -0.6 (-0.76--0.43) |
| North Macedonia | 299.35 (231.57-393.44) | 208.77 (138.16-283.65) | -0.3 | 37.7 (29.16-49.54) | 27.3 (18.07-37.09) | -0.99 (-1.19--0.8) |
| Northern Mariana Islands | 4.11 (2.23-7.71) | 2.93 (2.05-4.21) | -0.29 | 17.53 (9.51-32.91) | 17.77 (12.43-25.52) | 0.53 (-0.22-1.29) |
| Norway | 167.43 (133.12-223.94) | 140.7 (114.31-179.43) | -0.16 | 10.47 (8.32-14) | 7.93 (6.44-10.11) | -1.01 (-1.32--0.71) |
| Oman | 56.34 (33.16-86.87) | 144.68 (88.81-238.95) | 1.57 | 6.79 (4-10.47) | 6.25 (3.84-10.32) | 0.4 (0-0.8) |
| Pakistan | 18354.41 (10989.28-31373.23) | 40915.52 (27539.27-56724.22) | 1.23 | 44.98 (26.93-76.89) | 41.37 (27.84-57.35) | -0.73 (-0.86--0.59) |
| Palau | 2.73 (1.45-4.26) | 3.13 (1.94-4.98) | 0.15 | 39.11 (20.76-61.03) | 53.18 (32.92-84.54) | 0.99 (0.91-1.07) |
| Palestine | 90.96 (57.52-142.91) | 169.08 (122.44-233.29) | 0.86 | 11.85 (7.49-18.62) | 7.74 (5.61-10.68) | -1.56 (-1.68--1.44) |
| Panama | 231.87 (209.06-256.56) | 386.32 (311.28-463.09) | 0.67 | 22.92 (20.67-25.36) | 23.41 (18.86-28.06) | 0.35 (0.06-0.65) |
| Papua New Guinea | 350.07 (90.56-668.06) | 730.23 (320.9-1299.74) | 1.09 | 21.15 (5.47-40.36) | 17.06 (7.5-30.37) | -1.08 (-1.35--0.81) |
| Paraguay | 695.5 (491.85-989.53) | 1274.4 (938.41-1711.29) | 0.83 | 44.38 (31.38-63.14) | 41.65 (30.67-55.93) | 0.25 (0.09-0.42) |
| Peru | 7170.42 (5356.23-9341.95) | 7135.05 (5258.35-9524.55) | 0 | 80.83 (60.38-105.31) | 48.01 (35.38-64.09) | -1.86 (-2.11--1.62) |
| Philippines | 6216.31 (4285.21-7669.07) | 10633.22 (8067.36-14188.32) | 0.71 | 23.98 (16.53-29.59) | 22.5 (17.07-30.03) | -0.36 (-0.44--0.28) |
| Poland | 13546.51 (12872.21-14526.09) | 12179.82 (11177.19-13150.31) | -0.1 | 93.78 (89.11-100.56) | 100.67 (92.38-108.69) | -0.12 (-0.41-0.17) |
| Portugal | 1297.56 (1167.89-1443.01) | 398.34 (328.4-464.31) | -0.69 | 34.27 (30.85-38.11) | 13.5 (11.13-15.73) | -3.2 (-3.54--2.85) |
| Puerto Rico | 845.73 (766.35-929.97) | 298.62 (239.69-355.38) | -0.65 | 59.79 (54.18-65.74) | 28.87 (23.17-34.36) | -2.38 (-2.62--2.14) |
| Qatar | 30.42 (19.97-46.34) | 189.18 (122.24-290.26) | 5.22 | 12.87 (8.44-19.6) | 11.45 (7.4-17.57) | -0.05 (-0.28-0.19) |
| Republic of Korea | 4996.64 (3033.22-6801.99) | 1884.69 (1366.37-2632.33) | -0.62 | 23.74 (14.41-32.32) | 11.78 (8.54-16.45) | -2.68 (-2.93--2.43) |
| Republic of Moldova | 4180.44 (3622.72-4864.48) | 2285.64 (1882.81-2708.32) | -0.45 | 239.87 (207.87-279.12) | 184.29 (151.81-218.37) | -1.54 (-1.88--1.2) |
| Romania | 10705.28 (9327-12203.71) | 4148.11 (3454.17-4859.32) | -0.61 | 123.23 (107.37-140.48) | 76.97 (64.09-90.17) | -1.72 (-1.9--1.55) |
| Russian Federation | 60392.8 (51935.52-73776.8) | 114382.45 (98037.14-132129.53) | 0.89 | 103.82 (89.28-126.83) | 246.11 (210.94-284.3) | 2.37 (1.57-3.18) |
| Rwanda | 710.11 (456.9-1099.45) | 1475.14 (660.43-2768.73) | 1.08 | 25.91 (16.67-40.12) | 26.01 (11.64-48.82) | -0.2 (-0.57-0.17) |
| Saint Kitts and Nevis | 9.47 (7.9-11.1) | 8.26 (5.11-14.61) | -0.13 | 54.81 (45.72-64.21) | 36.34 (22.5-64.27) | -2.35 (-2.93--1.77) |
| Saint Lucia | 7.7 (6.55-8.95) | 9.71 (7.63-11.89) | 0.26 | 13.68 (11.64-15.9) | 14.7 (11.55-17.99) | 0.26 (0.02-0.51) |
| Saint Vincent and the Grenadines | 14.72 (13.06-16.6) | 14.37 (12.12-16.96) | -0.02 | 32.05 (28.43-36.14) | 34.78 (29.33-41.04) | 0 (-0.27-0.28) |
| Samoa | 23.44 (14.33-40.1) | 23.86 (14.52-38.07) | 0.02 | 35.01 (21.4-59.89) | 29.7 (18.07-47.38) | -0.59 (-0.65--0.54) |
| San Marino | 0.69 (0.51-0.89) | 0.46 (0.3-0.67) | -0.33 | 7.36 (5.47-9.51) | 5.15 (3.34-7.52) | -0.38 (-0.75--0.01) |
| Sao Tome and Principe | 17.47 (9.49-28.26) | 49.3 (26.68-87.21) | 1.82 | 40.69 (22.1-65.83) | 54.24 (29.35-95.95) | 0.93 (0.76-1.1) |
| Saudi Arabia | 813.99 (474.91-1323.54) | 2672.7 (1545.36-4061.61) | 2.28 | 12.25 (7.15-19.92) | 14.43 (8.34-21.93) | 1.07 (0.79-1.34) |
| Senegal | 1784.75 (1034.5-3210.39) | 4266.01 (2543.68-6930.27) | 1.39 | 64.68 (37.49-116.34) | 66.16 (39.45-107.48) | 0.35 (0.17-0.53) |
| Serbia | 2529.7 (1879.47-3317.37) | 1140.11 (871.51-1490.32) | -0.55 | 70.44 (52.34-92.38) | 38.46 (29.4-50.28) | -1.76 (-1.85--1.68) |
| Seychelles | 10.23 (6.71-17.78) | 10.62 (6.73-18.83) | 0.04 | 32.76 (21.49-56.96) | 27.66 (17.54-49.06) | -0.42 (-0.66--0.18) |
| Sierra Leone | 697.97 (348.19-1279.94) | 1995.61 (1156.03-3235.74) | 1.86 | 43.69 (21.8-80.13) | 53.51 (31-86.76) | 0.78 (0.52-1.05) |
| Singapore | 226.75 (197.76-264.14) | 124.63 (91.54-179.53) | -0.45 | 15.02 (13.1-17.5) | 6.48 (4.76-9.33) | -3.2 (-3.52--2.89) |
| Slovakia | 1889.64 (1409.92-2492.74) | 1156.13 (831.7-1484.74) | -0.39 | 92.31 (68.88-121.77) | 67.56 (48.6-86.76) | -0.57 (-0.77--0.38) |
| Slovenia | 444.16 (390.84-511.12) | 128.98 (108.81-151.42) | -0.71 | 57.96 (51-66.7) | 22.65 (19.11-26.59) | -3.14 (-3.36--2.92) |
| Solomon Islands | 29.61 (11.45-54.15) | 61.13 (37.85-93.6) | 1.06 | 23.08 (8.93-42.21) | 22.35 (13.84-34.22) | -0.13 (-0.22--0.04) |
| Somalia | 696.85 (351.14-1340.01) | 1818.15 (857.86-3903.71) | 1.61 | 24.04 (12.11-46.22) | 21.93 (10.35-47.09) | 0.03 (-0.13-0.19) |
| South Africa | 4412.5 (3363.57-5329.09) | 6057.1 (4352.23-8071.45) | 0.37 | 28.04 (21.38-33.87) | 24.98 (17.95-33.29) | -0.16 (-0.61-0.29) |
| South Sudan | 489.46 (223.07-1021.49) | 786.8 (471.81-1285.78) | 0.61 | 21.2 (9.66-44.25) | 21.86 (13.11-35.72) | 0.1 (-0.09-0.28) |
| Spain | 5030.21 (4632.68-5514.11) | 1698.15 (1501.72-1901.58) | -0.66 | 33.92 (31.24-37.18) | 13.68 (12.09-15.32) | -3.2 (-3.32--3.07) |
| Sri Lanka | 1516.07 (990.51-2195.54) | 941.31 (602.75-1417.62) | -0.38 | 20.5 (13.4-29.69) | 11.67 (7.47-17.58) | -2.08 (-2.24--1.93) |
| Sudan | 534.7 (320.87-842.85) | 1499.91 (958.26-2383.89) | 1.81 | 7.02 (4.21-11.06) | 8.11 (5.18-12.9) | 0.76 (0.64-0.87) |
| Suriname | 71.46 (50.46-93.42) | 92.97 (66.22-128.43) | 0.3 | 43.88 (30.98-57.37) | 43.31 (30.85-59.83) | -0.19 (-0.34--0.05) |
| Sweden | 491.34 (427.25-576.96) | 348.29 (290.27-428.93) | -0.29 | 16.74 (14.55-19.65) | 10.74 (8.95-13.23) | -0.99 (-1.31--0.67) |
| Switzerland | 372.09 (315.02-428.83) | 159.77 (133.85-182.96) | -0.57 | 14.12 (11.96-16.27) | 5.75 (4.82-6.59) | -3.32 (-3.49--3.16) |
| Syrian Arab Republic | 363.43 (259.31-487.52) | 279.45 (196.07-386.17) | -0.23 | 7.58 (5.41-10.16) | 5.49 (3.85-7.59) | -0.71 (-0.83--0.59) |
| Taiwan (Province of China) | 2875.06 (2607.55-3169.7) | 1416.66 (1225.53-1659.26) | -0.51 | 31.16 (28.26-34.35) | 18.78 (16.24-21.99) | -2.73 (-3.33--2.12) |
| Tajikistan | 517.98 (312.69-740.25) | 1164.62 (773.51-1661.86) | 1.25 | 24.49 (14.79-35.01) | 27.92 (18.54-39.84) | 0 (-0.32-0.31) |
| Thailand | 9429.41 (5582.54-14972.01) | 10684.82 (6062.68-15088.14) | 0.13 | 36.36 (21.53-57.74) | 50.4 (28.6-71.17) | 0.84 (0.54-1.15) |
| Timor-Leste | 116.21 (51.64-257.68) | 150.71 (67.79-270.26) | 0.3 | 36.51 (16.22-80.95) | 26.38 (11.86-47.3) | -1.4 (-1.86--0.95) |
| Togo | 712.95 (421.13-1153.89) | 2057.14 (1285.66-3200.01) | 1.89 | 52.01 (30.72-84.17) | 61.14 (38.21-95.11) | 0.52 (0.4-0.63) |
| Tokelau | 0.25 (0.12-0.45) | 0.21 (0.14-0.3) | -0.16 | 42.65 (20.36-77.8) | 41.73 (28.05-61.23) | -0.77 (-1.03--0.5) |
| Tonga | 14.63 (6.73-25) | 17.21 (10.25-27.47) | 0.18 | 39.66 (18.23-67.75) | 44.24 (26.35-70.63) | 0.45 (0.23-0.66) |
| Trinidad and Tobago | 137.94 (122.19-154.42) | 152.56 (117.11-195.66) | 0.11 | 27.51 (24.37-30.8) | 30.65 (23.53-39.31) | 0.2 (-0.09-0.49) |
| Tunisia | 202.75 (121.46-329.41) | 306.82 (201.46-441.86) | 0.51 | 5.89 (3.53-9.58) | 7.05 (4.63-10.15) | 0.71 (0.65-0.77) |
| Türkiye | 3400.71 (2285.92-4745.85) | 2684.58 (1926.63-3556.02) | -0.21 | 14.22 (9.56-19.84) | 8.43 (6.05-11.16) | -1.65 (-1.69--1.6) |
| Turkmenistan | 543.49 (435.92-672.58) | 1034.75 (737.8-1427.28) | 0.9 | 35.41 (28.4-43.82) | 49.75 (35.47-68.62) | 0.71 (0.53-0.9) |
| Tuvalu | 1.76 (1.04-2.8) | 2.03 (1.3-3.1) | 0.15 | 48.84 (28.94-77.64) | 40.88 (26.23-62.39) | -0.72 (-0.84--0.59) |
| Uganda | 817.67 (449.08-1461.92) | 2721.12 (1650.49-4444.79) | 2.33 | 12.75 (7-22.79) | 15.82 (9.6-25.84) | 0.33 (0.17-0.49) |
| Ukraine | 29737.46 (26005.07-34432.5) | 32710.03 (24145.8-42498.01) | 0.1 | 156.56 (136.91-181.28) | 237.29 (175.16-308.29) | 0.53 (0.21-0.86) |
| United Arab Emirates | 133.55 (85.69-202.01) | 451.76 (306.55-645.13) | 2.38 | 13.97 (8.96-21.13) | 11.24 (7.63-16.06) | -0.46 (-0.68--0.25) |
| United Kingdom | 3797.47 (3534.66-4220.96) | 3984.53 (3533.74-4508.99) | 0.05 | 18.17 (16.91-20.2) | 18.32 (16.25-20.73) | 0.02 (-0.32-0.38) |
| United Republic of Tanzania | 1744.98 (932.49-2762.19) | 5439.8 (2744.99-8921.45) | 2.12 | 18.01 (9.62-28.51) | 23.31 (11.76-38.23) | 1.13 (0.9-1.35) |
| United States of America | 23407.11 (21216.34-26770.77) | 25212.35 (23366.34-27620.98) | 0.08 | 22.91 (20.77-26.2) | 22.65 (20.99-24.82) | -0.16 (-0.38-0.05) |
| United States Virgin Islands | 14.07 (10.04-18.82) | 12.81 (7.52-20.87) | -0.09 | 35.52 (25.35-47.52) | 55.4 (32.53-90.22) | 2.3 (1.91-2.69) |
| Uruguay | 355.25 (319.76-391.06) | 319.38 (287.95-354.91) | -0.1 | 31.28 (28.15-34.43) | 26.7 (24.07-29.67) | -0.52 (-0.82--0.22) |
| Uzbekistan | 2559.17 (2023.22-3173.2) | 5442.15 (4269.46-6810.07) | 1.13 | 29.82 (23.57-36.97) | 39.62 (31.08-49.57) | 0.37 (-0.1-0.85) |
| Vanuatu | 28.51 (13.05-55.33) | 58.13 (32.21-97.04) | 1.04 | 48.67 (22.27-94.45) | 46.64 (25.84-77.85) | -0.35 (-0.5--0.2) |
| Venezuela (Bolivarian Republic of) | 2289.16 (2055.59-2571.64) | 3189.26 (2404.59-4044.31) | 0.39 | 28.58 (25.67-32.11) | 34.06 (25.68-43.2) | 0.76 (0.52-1) |
| Viet Nam | 7076.39 (4003.19-11633.58) | 9211.38 (4643.86-15311.6) | 0.3 | 24.81 (14.04-40.79) | 23.99 (12.1-39.88) | -0.46 (-0.67--0.25) |
| Yemen | 208.1 (115.6-316.37) | 738.43 (456.43-1090.95) | 2.55 | 4.53 (2.51-6.88) | 5.37 (3.32-7.93) | 0.78 (0.56-1.01) |
| Zambia | 599.84 (389.92-904.48) | 2025.4 (1170.37-3366.02) | 2.38 | 19.77 (12.85-29.81) | 25.03 (14.46-41.59) | 0.75 (0.59-0.92) |
| Zimbabwe | 843.49 (411.41-1326.47) | 2111.7 (1114.02-3524.07) | 1.5 | 21.28 (10.38-33.46) | 33.32 (17.58-55.61) | 1.59 (1.17-2.02) |
