# Supplementary material for: Global burden of pancreatitis among individuals aged 15–39 years: a systematic analysis from the 2021 GBD study
Source: Front Med (Lausanne). 2025 May 27;12:1572346. doi: 10.3389/fmed.2025.1572346 (PMC12150401; doi:10.3389/fmed.2025.1572346)
Supplement: Supplementary file 6 [file Supplementary_file_6.docx]

**Supplementary Table 6** The DALYs of pancreatitis cases and rates among WCBA in 1990 and 2021 across 204 countries, and the trends from 1990 to 2021

| **location** | **DALYs cases** | | | **DALYs rates** | | |
| --- | --- | --- | --- | --- | --- | --- |
|  | **1990**  **(95%UI)** | **2021**  **(95%UI)** | **percentage**  **Change**  **(100%)** | **1990**  **per (95%UI)** | **2021**  **per (95%UI)** | **EAPC**  **(95% CI)** |
| Afghanistan | 199.36 (155.51-262.28) | 796.03 (584.09-1101.79) | 2.99 | 9060.82 (7067.64-11920.28) | 11063.25 (8117.62-15312.68) | 0.19 (0.02-0.36) |
| Albania | 27.53 (21.12-36.36) | 30.77 (21.04-43.33) | 0.12 | 3296.52 (2528.65-4353.75) | 5013.94 (3427.93-7059.45) | 0.52 (0.25-0.8) |
| Algeria | 435.63 (329.71-587.22) | 976.74 (699.04-1394.86) | 1.24 | 7496.25 (5673.64-10104.8) | 8702.75 (6228.48-12428.22) | 0.2 (0.01-0.39) |
| American Samoa | 0.32 (0.25-0.42) | 0.36 (0.26-0.49) | 0.12 | 2673.83 (2078.23-3523.25) | 3069.68 (2177-4217.07) | 0.08 (-0.06-0.22) |
| Andorra | 1.09 (0.85-1.42) | 1.81 (1.27-2.58) | 0.66 | 7280.27 (5696.14-9541.67) | 9068.52 (6358.21-12922.55) | 0.16 (-0.1-0.43) |
| Angola | 243.49 (187.38-316.61) | 903.25 (647.14-1272.21) | 2.71 | 10577.3 (8139.56-13753.53) | 11768.66 (8431.74-16576.01) | -0.02 (-0.16-0.12) |
| Antigua and Barbuda | 0.79 (0.6-1.08) | 1.6 (1.1-2.31) | 1.03 | 4889.9 (3672.58-6632.58) | 6603.26 (4559.3-9579.93) | 0.4 (0.13-0.67) |
| Argentina | 423.81 (332.42-553.92) | 769.33 (578.69-983.61) | 0.82 | 5279.93 (4141.42-6900.92) | 6472.41 (4868.58-8275.19) | -0.02 (-0.27-0.24) |
| Armenia | 30 (23.44-39.54) | 38.47 (26.93-53.77) | 0.28 | 3463.77 (2706.54-4565.7) | 5221.58 (3654.8-7297.9) | 0.57 (0.26-0.88) |
| Australia | 399.63 (336.58-475.28) | 610.18 (451.36-817.31) | 0.53 | 8953.19 (7540.73-10648.18) | 10128.64 (7492.24-13566.8) | 0.21 (0.01-0.41) |
| Austria | 136.93 (112.53-171.16) | 137.7 (97.81-187.27) | 0.01 | 6919.46 (5686.48-8649.51) | 6973.97 (4953.85-9484.28) | -0.7 (-1.01--0.39) |
| Azerbaijan | 63.93 (48.71-86.35) | 135.32 (93.33-188.01) | 1.12 | 3409.36 (2597.58-4604.82) | 4936.51 (3404.78-6858.77) | 0.51 (0.21-0.81) |
| Bahamas | 3.56 (2.71-4.66) | 7.35 (5.17-10.35) | 1.06 | 4905.59 (3738.68-6420.78) | 6818.55 (4796.16-9602.72) | 0.44 (0.15-0.73) |
| Bahrain | 11.19 (8.73-14.84) | 34.81 (24.23-48.21) | 2.11 | 9708.43 (7567.63-12868.55) | 10677.66 (7431.42-14786.09) | -0.38 (-0.59--0.18) |
| Bangladesh | 2082.19 (1656.49-2774.96) | 4537.58 (3269.34-6146.09) | 1.18 | 8457.45 (6728.35-11271.35) | 9865.31 (7107.99-13362.44) | 0.1 (-0.09-0.28) |
| Barbados | 3.37 (2.56-4.48) | 5 (3.51-7) | 0.48 | 4921.67 (3740.73-6539.06) | 7026.34 (4924.8-9837.95) | 0.45 (0.21-0.7) |
| Belarus | 149.35 (118.67-190.03) | 204.57 (145.23-276.41) | 0.37 | 5894.45 (4683.68-7500.03) | 9646.22 (6848.19-13034.13) | 0.43 (0.13-0.73) |
| Belgium | 151.8 (135.51-170.98) | 217.18 (163.77-289.45) | 0.43 | 6235.28 (5566.37-7023.15) | 8765.77 (6609.89-11682.78) | 0.94 (0.72-1.16) |
| Belize | 2.05 (1.56-2.82) | 8 (5.59-11.29) | 2.9 | 4873.23 (3706.58-6704.32) | 6622 (4631.8-9345.46) | 0.52 (0.3-0.75) |
| Benin | 63.77 (49.39-85.38) | 209.98 (148.13-300.18) | 2.29 | 5821.65 (4508.8-7794.23) | 6472.74 (4566.05-9252.93) | 0.09 (0-0.18) |
| Bermuda | 1.06 (0.82-1.37) | 0.94 (0.62-1.34) | -0.11 | 6181.15 (4786.23-8004.15) | 6942.4 (4630.16-9909) | -0.37 (-0.64--0.1) |
| Bhutan | 8.24 (6.4-10.81) | 12.07 (8.37-17.16) | 0.46 | 5755.16 (4473.62-7548.36) | 5829.18 (4044.05-8290.21) | -0.12 (-0.3-0.06) |
| Bolivia (Plurinational State of) | 95.15 (75.45-124.16) | 269.33 (193.99-387.03) | 1.83 | 6219.7 (4931.96-8115.83) | 8638.04 (6221.69-12412.78) | 0.11 (-0.32-0.53) |
| Bosnia and Herzegovina | 60.36 (47.86-77.77) | 41.86 (29.26-57.85) | -0.31 | 5184.34 (4110.84-6679.5) | 5721.73 (3998.76-7907) | -0.84 (-1.17--0.5) |
| Botswana | 18.13 (14.12-24.26) | 56.63 (40.25-80.46) | 2.12 | 5631.87 (4386.52-7534.61) | 8322.8 (5915.95-11826.33) | 0.39 (0.19-0.6) |
| Brazil | 3094.22 (2599.4-3717.05) | 5689.79 (4668.16-6942.34) | 0.84 | 7943.9 (6673.54-9542.93) | 9690.38 (7950.42-11823.63) | -0.37 (-0.77-0.03) |
| Brunei Darussalam | 1.82 (1.4-2.47) | 3.97 (2.77-5.63) | 1.18 | 2694.32 (2068.38-3662.61) | 3181.36 (2220.41-4506.85) | 0.14 (-0.11-0.39) |
| Bulgaria | 89.39 (72.3-113.96) | 84.9 (58.5-119.6) | -0.05 | 4316.78 (3491.14-5503.26) | 5960.44 (4106.64-8396.1) | -0.33 (-0.72-0.06) |
| Burkina Faso | 117.47 (92.26-152.8) | 314.43 (224.32-447.62) | 1.68 | 5611.96 (4407.63-7300.08) | 5724.33 (4083.86-8149.16) | 0.41 (0.25-0.57) |
| Burundi | 95.52 (73.46-124.4) | 228.42 (154.33-326.46) | 1.39 | 7584.73 (5832.87-9877.42) | 7303.63 (4934.61-10438.38) | -0.86 (-1.1--0.62) |
| Cabo Verde | 4.34 (3.35-5.98) | 12.14 (8.51-16.96) | 1.8 | 5472.77 (4228.54-7542.16) | 8080.09 (5662.67-11284.57) | 0.64 (0.43-0.86) |
| Cambodia | 102.89 (79.78-137.48) | 187.03 (132.18-252.5) | 0.82 | 4108.34 (3185.78-5489.68) | 4139.23 (2925.26-5588) | -0.96 (-1.19--0.72) |
| Cameroon | 145.76 (114.12-194.47) | 534.25 (380.08-770.4) | 2.67 | 6125.8 (4796.38-8173.16) | 6820.76 (4852.42-9835.75) | 0.09 (0.01-0.18) |
| Canada | 450.8 (383.77-550.93) | 706.45 (512.65-966.79) | 0.57 | 6128.23 (5217.06-7489.45) | 8517.72 (6180.97-11656.6) | 0.03 (-0.31-0.37) |
| Central African Republic | 67.52 (52.29-88.16) | 154.55 (109.32-215.32) | 1.29 | 10533.68 (8158.1-13753.71) | 11171.25 (7902.12-15564.01) | 0.02 (-0.1-0.15) |
| Chad | 97.62 (75.68-129.99) | 295.65 (206.52-408.51) | 2.03 | 7341.79 (5691.46-9776.25) | 7643.42 (5339.23-10561.4) | 0.04 (-0.05-0.14) |
| Chile | 377.63 (328.09-447.34) | 524.95 (380.42-729.58) | 0.39 | 10447.63 (9077.08-12376.27) | 11145.07 (8076.64-15489.37) | -0.46 (-0.73--0.19) |
| China | 13190.76 (11022.29-15889.85) | 8821.24 (7223.47-10719.28) | -0.33 | 4093.83 (3420.83-4931.51) | 2767.9 (2266.56-3363.47) | -1.32 (-1.62--1.02) |
| Colombia | 294.23 (224.72-388.05) | 421.8 (295.59-587.86) | 0.43 | 3387.82 (2587.47-4468.11) | 3219.76 (2256.34-4487.35) | -1.07 (-1.51--0.62) |
| Comoros | 5.61 (4.38-7.5) | 12.5 (8.69-17.69) | 1.23 | 5320.12 (4158.93-7117.91) | 6401.25 (4448.9-9063.91) | 0.08 (-0.1-0.27) |
| Congo | 58.36 (44.21-76.61) | 165.58 (117.39-233.86) | 1.84 | 10353.58 (7844.23-13591.59) | 11556.7 (8193.48-16322.28) | -0.03 (-0.23-0.17) |
| Cook Islands | 0.17 (0.13-0.24) | 0.19 (0.13-0.27) | 0.12 | 3690.2 (2739.17-5162.63) | 4303.28 (2932.89-6315.34) | 0.13 (0.03-0.23) |
| Costa Rica | 38.6 (29.32-51.26) | 92.03 (64.68-130.11) | 1.38 | 4964.1 (3770.34-6591.18) | 7108.9 (4996.5-10051.11) | 0.62 (0.41-0.82) |
| C么te d'Ivoire | 135.94 (104.66-184.17) | 354.34 (250.99-499.3) | 1.61 | 4967.78 (3824.74-6730.12) | 5321.31 (3769.34-7498.29) | 0.07 (-0.03-0.17) |
| Croatia | 62.13 (49.9-78.73) | 47.51 (34.37-66.17) | -0.24 | 5174.18 (4156.33-6556.78) | 5295.81 (3831.62-7376.71) | -0.79 (-1.09--0.49) |
| Cuba | 308.59 (252.54-382.67) | 200 (147.48-281.31) | -0.35 | 10071.18 (8241.7-12488.59) | 8035.87 (5925.43-11302.76) | -1.73 (-2.08--1.37) |
| Cyprus | 11.7 (9.02-15.69) | 25.59 (17.83-36.91) | 1.19 | 5921.38 (4565.12-7941.04) | 7166.76 (4991.55-10334.08) | 0.18 (-0.05-0.41) |
| Czechia | 131.16 (104.79-163.03) | 129.75 (92.19-176.67) | -0.01 | 5094.46 (4070.2-6332.47) | 5656.34 (4018.86-7701.58) | -0.62 (-0.94--0.31) |
| Democratic People's Republic of Korea | 179.39 (143.97-230.75) | 197.05 (139.76-268.39) | 0.1 | 3163.3 (2538.81-4069) | 2990.27 (2120.89-4072.8) | -0.44 (-0.53--0.35) |
| Democratic Republic of the Congo | 832.56 (643.48-1091.71) | 2151.73 (1527.65-2937.96) | 1.58 | 9755.05 (7539.66-12791.54) | 10098.45 (7169.56-13788.37) | -0.05 (-0.17-0.07) |
| Denmark | 124.35 (101.34-151.33) | 105.36 (77.65-143.7) | -0.15 | 9523.72 (7761.44-11590.51) | 8341.9 (6148.14-11376.85) | -1.1 (-1.4--0.78) |
| Djibouti | 5.53 (4.23-7.44) | 22.33 (15.91-30.79) | 3.04 | 5630.88 (4304.4-7567.22) | 6948.88 (4952.69-9583.14) | 0.33 (0.18-0.49) |
| Dominica | 0.82 (0.62-1.12) | 1.09 (0.76-1.53) | 0.33 | 4825.9 (3658.07-6594.64) | 6655.72 (4652.61-9321.71) | 0.4 (0.14-0.66) |
| Dominican Republic | 121.68 (92.7-166.13) | 231.66 (160.7-328.09) | 0.9 | 6445.17 (4910.19-8799.71) | 7997.13 (5547.57-11325.76) | 0.28 (0.06-0.49) |
| Ecuador | 138.74 (108.63-179.26) | 355.06 (256.15-481.32) | 1.56 | 5501.03 (4307.1-7107.66) | 7529.44 (5432.01-10206.97) | 0.3 (-0.03-0.64) |
| Egypt | 886.97 (678.7-1194.97) | 2236.1 (1566.27-3098.18) | 1.52 | 6763.86 (5175.61-9112.63) | 8617.73 (6036.26-11940.06) | 0.32 (0.08-0.55) |
| El Salvador | 97.73 (76.38-125.27) | 139.96 (100.77-194.54) | 0.43 | 7456.01 (5827.35-9556.67) | 7879.53 (5673.32-10952.2) | -0.42 (-0.68--0.16) |
| Equatorial Guinea | 10.56 (8.17-13.83) | 41.74 (30.87-58.37) | 2.95 | 10692.35 (8272.25-13998.2) | 11452.94 (8468.66-16015.74) | 0.05 (-0.09-0.19) |
| Eritrea | 51.21 (39.26-65.84) | 117.56 (84.48-164.86) | 1.3 | 6494.37 (4978.17-8349.08) | 7102.4 (5103.76-9960.01) | -0.03 (-0.16-0.09) |
| Estonia | 29.97 (23.85-37.45) | 21.58 (15.29-29.08) | -0.28 | 7864.59 (6257.95-9827.5) | 7751.35 (5493.07-10445.54) | -1.29 (-1.61--0.97) |
| Eswatini | 10.87 (8.38-14.15) | 30.82 (21.52-42.92) | 1.84 | 5579.6 (4302.19-7261.76) | 9779.95 (6826.45-13618.6) | 0.78 (0.45-1.1) |
| Ethiopia | 690.16 (562.51-867.39) | 1752.55 (1373.1-2246.34) | 1.54 | 6116.48 (4985.25-7687.18) | 6322.54 (4953.63-8103.96) | -0.42 (-0.67--0.17) |
| Fiji | 6.33 (5.04-8.32) | 9.14 (6.58-12.69) | 0.44 | 3235.92 (2576.84-4256.7) | 4003.97 (2882.87-5556.57) | 0.01 (-0.16-0.19) |
| Finland | 128.61 (104.66-159.54) | 122.08 (89.18-164.2) | -0.05 | 10180.04 (8284.28-12628.7) | 10741.64 (7846.81-14447.52) | -0.63 (-0.91--0.35) |
| France | 1342.17 (1194.06-1513.16) | 1416.8 (1046.59-1955.72) | 0.06 | 9274.31 (8250.87-10455.83) | 9979.32 (7371.72-13775.26) | -0.28 (-0.51--0.05) |
| Gabon | 20.78 (15.95-27.46) | 52.97 (37.78-73.19) | 1.55 | 9388.38 (7203.23-12405.5) | 10820.79 (7717.63-14949.21) | -0.04 (-0.2-0.12) |
| Gambia | 23.64 (18.44-31.64) | 68.9 (47.79-96.41) | 1.91 | 10418.16 (8125.48-13943.44) | 11203.49 (7770.4-15676.97) | -0.4 (-0.59--0.21) |
| Georgia | 55.06 (42.44-72.73) | 44.95 (31.54-62.36) | -0.18 | 4011 (3092.16-5298.64) | 5712.3 (4008.27-7923.47) | 0.39 (0.13-0.65) |
| Germany | 1056.84 (904.01-1261.08) | 1382.16 (1001.29-1879.69) | 0.31 | 5437.77 (4651.4-6488.63) | 8115.67 (5879.33-11037.06) | 1.24 (1.04-1.44) |
| Ghana | 206.17 (155.84-269.91) | 604.2 (426.89-859.21) | 1.93 | 5845.63 (4418.8-7652.93) | 6617.5 (4675.47-9410.54) | 0.03 (-0.1-0.16) |
| Greece | 262 (203.61-345.82) | 292.55 (201.49-415.94) | 0.12 | 10397.9 (8080.62-13724.5) | 13496.97 (9295.93-19189.32) | -0.01 (-0.42-0.41) |
| Greenland | 2.62 (2.09-3.28) | 2.52 (1.88-3.34) | -0.04 | 17755.16 (14200.12-22235.46) | 19802.85 (14804.12-26237.74) | -0.39 (-0.64--0.14) |
| Grenada | 0.96 (0.73-1.31) | 1.7 (1.17-2.35) | 0.77 | 4936.53 (3753.17-6723.91) | 6654.87 (4569.85-9162.48) | 0.39 (0.18-0.6) |
| Guam | 1.17 (0.94-1.49) | 1.52 (1.1-2.13) | 0.3 | 3348.6 (2685.65-4284.13) | 4211 (3055.59-5901.16) | 0.24 (0.02-0.47) |
| Guatemala | 114.37 (87.62-153.22) | 360.02 (259.18-525.37) | 2.15 | 6225.15 (4769.37-8340.25) | 8190.82 (5896.68-11952.72) | 0.18 (-0.07-0.43) |
| Guinea | 83.38 (64.29-111.12) | 229.43 (164.69-324.42) | 1.75 | 6112.83 (4713.34-8146.34) | 6913.54 (4962.74-9775.77) | 0.07 (-0.07-0.21) |
| Guinea-Bissau | 14.41 (11.2-19.06) | 37.44 (26.41-52.25) | 1.6 | 6177.45 (4802.05-8172.61) | 7127.3 (5027.87-9947.27) | 0.21 (0.08-0.34) |
| Guyana | 17.1 (13.3-21.42) | 26.25 (19.31-36.99) | 0.54 | 8369.75 (6510.57-10488.54) | 12917.04 (9505.57-18206) | 0.69 (0.47-0.9) |
| Haiti | 101.46 (78.12-136.65) | 277.8 (192.82-381.19) | 1.74 | 6594.06 (5077.05-8881.68) | 7847.38 (5446.94-10768.06) | 0.03 (-0.17-0.22) |
| Honduras | 49.46 (37.4-65.51) | 195.75 (135.43-270.39) | 2.96 | 4687.99 (3545.12-6209.67) | 6896.37 (4771.28-9525.91) | 0.52 (0.23-0.8) |
| Hungary | 137.27 (111.45-172.41) | 109.15 (77.81-145.89) | -0.2 | 5415.48 (4396.78-6801.9) | 5103.12 (3637.73-6820.94) | -1.13 (-1.43--0.82) |
| Iceland | 4.08 (3.27-5.22) | 4.85 (3.42-6.74) | 0.19 | 6295.54 (5046.85-8042.1) | 6089.98 (4290.6-8465.75) | -0.52 (-0.73--0.32) |
| India | 12749.38 (10529.81-15630.7) | 24394.49 (20040.28-29811.35) | 0.91 | 6314.47 (5215.17-7741.52) | 6448.63 (5297.61-7880.57) | -1.27 (-1.67--0.86) |
| Indonesia | 1148.76 (921.44-1441.44) | 2491.31 (1992.11-3094.79) | 1.17 | 2397.01 (1922.69-3007.72) | 3308.8 (2645.79-4110.3) | 0.3 (0.01-0.59) |
| Iran (Islamic Republic of) | 1253 (995.81-1591.83) | 2791.7 (2217.57-3501.12) | 1.23 | 9907.15 (7873.6-12586.17) | 12009.69 (9539.81-15061.52) | 0.77 (0.53-1) |
| Iraq | 293.7 (227.94-376.51) | 858.77 (623.39-1224.12) | 1.92 | 7238.06 (5617.48-9278.85) | 8145.77 (5913.12-11611.25) | 0.29 (-0.25-0.82) |
| Ireland | 68.7 (56.91-83.51) | 129.81 (97.14-172.98) | 0.89 | 7780.38 (6444.31-9457.33) | 11126.42 (8326.1-14826.19) | 0.45 (0.19-0.71) |
| Israel | 102.66 (80.72-133.31) | 214.89 (155.66-301.14) | 1.09 | 8415.53 (6617.2-10927.95) | 9660.37 (6997.72-13537.62) | -0.37 (-0.61--0.13) |
| Italy | 1060.15 (864.55-1323.68) | 1161.12 (934.69-1448.95) | 0.1 | 7417.37 (6048.84-9261.12) | 9569.66 (7703.47-11941.89) | -0.29 (-0.65-0.07) |
| Jamaica | 29 (21.82-39.64) | 52.85 (36.71-76.16) | 0.82 | 4866.43 (3662.04-6652.2) | 6845.22 (4754.45-9863.59) | 0.39 (0.1-0.68) |
| Japan | 1150.22 (986.3-1357.41) | 1221.25 (1040.9-1443.04) | 0.06 | 3582.91 (3072.3-4228.3) | 4911.25 (4185.98-5803.19) | 0.59 (0.29-0.9) |
| Jordan | 71.22 (55.54-95.14) | 285.26 (203.74-409.97) | 3.01 | 8539.99 (6659.49-11408.72) | 9204.82 (6574.42-13229.11) | -0.23 (-0.39--0.06) |
| Kazakhstan | 186.51 (149.32-231.24) | 279.87 (202.47-371.72) | 0.5 | 4532.58 (3628.63-5619.61) | 5909.62 (4275.31-7849.12) | 0.6 (0.42-0.79) |
| Kenya | 325.75 (268.47-402.17) | 894.83 (729.23-1102.8) | 1.75 | 6289.23 (5183.4-7764.76) | 6741.94 (5494.22-8308.81) | -0.22 (-0.41--0.04) |
| Kiribati | 0.64 (0.5-0.85) | 1.09 (0.78-1.51) | 0.7 | 3436.26 (2685.08-4522.42) | 3411.91 (2450.05-4724.15) | -0.37 (-0.52--0.23) |
| Kuwait | 31.74 (24.27-42.36) | 115.87 (79.82-169.41) | 2.65 | 7658.21 (5855.8-10220.6) | 7927.05 (5460.71-11589.7) | -0.05 (-0.17-0.07) |
| Kyrgyzstan | 49 (38.61-61.97) | 104.14 (75.94-139.08) | 1.13 | 4674.9 (3683.8-5911.88) | 6040.46 (4404.84-8066.98) | 0.17 (-0.07-0.41) |
| Lao People's Democratic Republic | 30.23 (23.34-39.41) | 59.19 (41.4-83.04) | 0.96 | 3123.77 (2412.34-4072.93) | 2982.82 (2086.16-4184.77) | -0.71 (-1.05--0.37) |
| Latvia | 43.29 (34.36-54.48) | 31.83 (22.23-43.55) | -0.26 | 6678.92 (5301.8-8406.06) | 8167.67 (5705.54-11174.67) | -0.36 (-0.65--0.06) |
| Lebanon | 56.95 (45.24-75.1) | 189.8 (132.44-259.57) | 2.33 | 7599.18 (6037.58-10021.36) | 12793.32 (8927.13-17495.79) | 0.57 (0.3-0.85) |
| Lesotho | 36.96 (29.26-47.85) | 66.57 (49.05-90.79) | 0.8 | 9769.17 (7733.9-12647.54) | 13132.88 (9675.77-17910.29) | 0.38 (0.09-0.67) |
| Liberia | 43.25 (33.73-56.82) | 116.45 (85.1-165.62) | 1.69 | 7795.22 (6080.12-10241.27) | 8345.49 (6099.08-11869.59) | 0.55 (0.27-0.84) |
| Libya | 70.12 (54-94.74) | 185.66 (130.76-258.35) | 1.65 | 7699.47 (5929.43-10402.33) | 9333.77 (6573.42-12987.88) | 0.33 (0.18-0.49) |
| Lithuania | 62.8 (50.86-79.94) | 55.16 (40.44-75.88) | -0.12 | 6812.33 (5516.99-8671.23) | 9627.2 (7058.03-13244.14) | -0.11 (-0.42-0.21) |
| Luxembourg | 7.59 (6-9.67) | 11.19 (8.21-14.57) | 0.47 | 7810.64 (6176.37-9954.75) | 7185.42 (5275.26-9361.71) | -0.92 (-1.19--0.65) |
| Madagascar | 166.87 (131.4-220.29) | 517.33 (358.76-737.47) | 2.1 | 6196 (4878.77-8179.42) | 7142.05 (4952.88-10181.3) | 0 (-0.17-0.18) |
| Malawi | 122.91 (93.43-161.78) | 304.42 (216.93-425.82) | 1.48 | 5443.74 (4138.06-7165.06) | 6077.15 (4330.59-8500.56) | -0.34 (-0.56--0.13) |
| Malaysia | 156.04 (123.49-206.03) | 370.38 (255.82-519.69) | 1.37 | 3482.64 (2756.17-4598.46) | 4395.32 (3035.77-6167.18) | 1.11 (0.82-1.4) |
| Maldives | 2.65 (2.1-3.44) | 5.2 (3.72-7.24) | 0.96 | 5562.96 (4403.32-7220.96) | 4514.43 (3226.06-6286.57) | -1.48 (-1.73--1.23) |
| Mali | 91.75 (71.69-119.43) | 263.98 (184.56-372.85) | 1.88 | 4781.33 (3736.12-6223.82) | 4848.76 (3389.9-6848.39) | -0.21 (-0.39--0.02) |
| Malta | 5.61 (4.28-7.32) | 6.63 (4.65-9.28) | 0.18 | 5916.92 (4513.06-7723.8) | 7035.67 (4935.92-9845.38) | 0.21 (-0.04-0.46) |
| Marshall Islands | 0.31 (0.25-0.42) | 0.52 (0.37-0.72) | 0.68 | 3198.51 (2516.16-4288.76) | 3486.61 (2499.53-4876.92) | -0.19 (-0.31--0.07) |
| Mauritania | 20.35 (15.81-27.45) | 49.99 (35.29-71.97) | 1.46 | 4329.26 (3363.62-5840.05) | 4656.95 (3287.57-6704.49) | -0.1 (-0.27-0.08) |
| Mauritius | 21.33 (16.95-26.88) | 24.29 (17.26-33.24) | 0.14 | 7140.78 (5674.23-8996.57) | 7673.27 (5454.8-10503.02) | -0.57 (-0.82--0.32) |
| Mexico | 1053.52 (863.06-1300.89) | 3429.57 (2783.79-4168.46) | 2.26 | 4812.11 (3942.13-5942.01) | 9786.48 (7943.7-11894.95) | 2.14 (1.83-2.44) |
| Micronesia (Federated States of) | 0.77 (0.6-1.02) | 0.9 (0.66-1.23) | 0.17 | 3322.39 (2583.42-4404.61) | 3456.76 (2507.56-4706.15) | -0.25 (-0.37--0.13) |
| Monaco | 0.57 (0.42-0.77) | 0.76 (0.52-1.09) | 0.33 | 8194.23 (6063.27-10997.73) | 10574.5 (7311.63-15281.22) | 0.23 (0.04-0.42) |
| Mongolia | 31.28 (24.75-40.36) | 53.63 (38.79-74.19) | 0.71 | 6111.73 (4836.96-7885.9) | 6355.72 (4597.11-8792.48) | -0.12 (-0.18--0.07) |
| Montenegro | 6.48 (5.18-8.22) | 8.62 (6.2-11.98) | 0.33 | 4151.11 (3319-5269.42) | 5988.37 (4306.52-8315.6) | 0.22 (-0.07-0.51) |
| Morocco | 592.09 (464.97-776.52) | 1108.52 (803.62-1520.96) | 0.87 | 9401.78 (7383.28-12330.33) | 11444.82 (8296.88-15702.97) | 0.13 (-0.1-0.37) |
| Mozambique | 193.64 (150.56-259.7) | 576.45 (399.08-840.04) | 1.98 | 6136.5 (4771.38-8229.92) | 7592.57 (5256.45-11064.35) | 0.15 (-0.01-0.31) |
| Myanmar | 167.9 (124.82-227.72) | 362.2 (251.96-516.22) | 1.16 | 1610.96 (1197.6-2185.01) | 2395.86 (1666.62-3414.62) | 0.35 (-0.15-0.85) |
| Namibia | 15.55 (12.1-20.76) | 45.43 (31.98-64.64) | 1.92 | 4584.56 (3566.76-6121.82) | 6869.96 (4835.36-9774.09) | 0.13 (-0.27-0.53) |
| Nauru | 0.09 (0.06-0.12) | 0.12 (0.08-0.18) | 0.33 | 3646.42 (2662.44-5059.72) | 4294.34 (2925.2-6249.97) | 0.16 (0.06-0.26) |
| Nepal | 294.93 (230.36-379) | 887.78 (617.16-1222.08) | 2.01 | 6457.18 (5043.36-8297.75) | 9786.67 (6803.4-13471.97) | 0.78 (0.51-1.05) |
| Netherlands | 293.79 (259.72-333.22) | 327.95 (234.84-449.24) | 0.12 | 7415.48 (6555.6-8410.71) | 8937 (6399.7-12242.3) | -0.14 (-0.43-0.16) |
| New Zealand | 65.13 (51.92-83.26) | 92.76 (70.01-123.58) | 0.42 | 7204.65 (5743.42-9210.96) | 7763.97 (5859.74-10343.63) | 0.06 (-0.01-0.13) |
| Nicaragua | 50.3 (39.16-66.87) | 130.58 (91.15-178.47) | 1.6 | 5574.63 (4340.06-7410.9) | 7202.66 (5028.14-9844.28) | 0.17 (-0.06-0.4) |
| Niger | 101.74 (78.76-131.2) | 300.49 (209.65-414.14) | 1.95 | 5848.7 (4527.89-7542.25) | 5636.62 (3932.56-7768.3) | -0.11 (-0.17--0.05) |
| Nigeria | 1088.39 (877.55-1369.81) | 2630.44 (2105.37-3316.75) | 1.42 | 5374.25 (4333.16-6763.86) | 4581 (3666.57-5776.23) | -0.79 (-1.01--0.57) |
| Niue | 0.02 (0.01-0.02) | 0.02 (0.01-0.02) | 0 | 3656.48 (2691.69-4957.59) | 4271.16 (2971.73-6024.75) | 0.14 (0.04-0.24) |
| North Macedonia | 19.26 (15.38-24.62) | 28.9 (20.77-39.16) | 0.5 | 3784.97 (3021.84-4838.2) | 5444.84 (3914.14-7377.96) | 0.03 (-0.36-0.43) |
| Northern Mariana Islands | 0.35 (0.26-0.47) | 0.39 (0.29-0.56) | 0.11 | 2504.41 (1910.09-3401.93) | 3444.02 (2496.06-4918) | 0.41 (0.17-0.64) |
| Norway | 72.08 (58.87-88.97) | 105.48 (83.18-132.95) | 0.46 | 6829.27 (5577.31-8429.8) | 8709.3 (6867.74-10977.58) | 0.3 (0.11-0.49) |
| Oman | 24.39 (18.81-31.92) | 92.49 (62.12-132.93) | 2.79 | 7194.34 (5548.42-9416.9) | 9108.2 (6116.8-13090.5) | 0.34 (0.1-0.58) |
| Pakistan | 1430.8 (1148.27-1797.41) | 4192.54 (3262.18-5422.38) | 1.93 | 6048.72 (4854.32-7598.54) | 6921.32 (5385.42-8951.62) | 0.14 (-0.08-0.35) |
| Palau | 0.15 (0.11-0.2) | 0.16 (0.11-0.23) | 0.07 | 3666.51 (2700.78-4999.16) | 4271.16 (2926.35-6076.86) | 0.13 (0.04-0.23) |
| Palestine | 46.18 (35.15-61.76) | 162.27 (115.3-228.47) | 2.51 | 10446.83 (7952.33-13971.17) | 12461.86 (8855.22-17546.47) | 0.09 (-0.12-0.3) |
| Panama | 28.59 (21.61-37.8) | 68 (46.66-95.14) | 1.38 | 4671.82 (3530.59-6176.89) | 6361.32 (4365.14-8901.13) | 0.43 (0.16-0.71) |
| Papua New Guinea | 37.47 (29.09-49.88) | 99.92 (70.81-139.41) | 1.67 | 3888.25 (3018.81-5176.01) | 3819.45 (2706.7-5328.73) | -0.28 (-0.34--0.23) |
| Paraguay | 56.8 (44.93-75.29) | 167.6 (120.59-233.7) | 1.95 | 6041.2 (4779.34-8008.36) | 8834.88 (6356.91-12319.29) | 0.53 (0.31-0.75) |
| Peru | 183.24 (140.57-239.74) | 440.24 (312.48-617.12) | 1.4 | 3373.06 (2587.6-4413.29) | 4576.82 (3248.61-6415.75) | -0.12 (-0.61-0.36) |
| Philippines | 533.47 (429.05-665.48) | 1212.82 (986.18-1528.05) | 1.27 | 3437.5 (2764.65-4288.06) | 4137.48 (3364.32-5212.89) | -0.11 (-0.42-0.21) |
| Poland | 229.78 (183.14-288.99) | 284.15 (223.19-359.84) | 0.24 | 2446.18 (1949.61-3076.5) | 3218.69 (2528.21-4076.06) | -0.36 (-0.82-0.11) |
| Portugal | 282.01 (225.62-355.12) | 298.25 (215.58-423.43) | 0.06 | 11146.92 (8918.23-14036.59) | 12812.29 (9260.6-18189.72) | -0.46 (-0.81--0.11) |
| Puerto Rico | 37.82 (29.33-48.74) | 37.7 (26.32-53.4) | 0 | 3947.93 (3061.69-5087.42) | 5027.44 (3510.48-7121.84) | 0.24 (-0.02-0.5) |
| Qatar | 6.94 (5.4-8.94) | 50.61 (35.14-72.19) | 6.29 | 8777.14 (6827.17-11311.07) | 9121.79 (6333.78-13013.04) | -0.3 (-0.48--0.13) |
| Republic of Korea | 448.32 (376.46-532.75) | 486.01 (359.08-632.48) | 0.08 | 3548.22 (2979.47-4216.47) | 4192.34 (3097.5-5455.8) | 0.28 (0.12-0.45) |
| Republic of Moldova | 61.32 (48.16-77.77) | 55.68 (38.89-78.81) | -0.09 | 5448.62 (4278.82-6909.8) | 6312.46 (4409.3-8934.38) | -0.46 (-0.78--0.14) |
| Romania | 205.89 (161.94-262.12) | 209.04 (150.09-283.44) | 0.02 | 3661.5 (2879.89-4661.4) | 5148.03 (3696.28-6980.35) | 0.21 (-0.12-0.53) |
| Russian Federation | 1661.24 (1328.74-2086.61) | 2106.46 (1675.88-2594.56) | 0.27 | 4486.74 (3588.72-5635.58) | 6214.89 (4944.51-7654.99) | 0.19 (-0.18-0.55) |
| Rwanda | 137.65 (107.64-184.77) | 307.94 (216.75-420.09) | 1.24 | 8483.77 (6634.39-11388.06) | 8744.5 (6154.84-11929.17) | -0.81 (-1.06--0.57) |
| Saint Kitts and Nevis | 0.64 (0.47-0.9) | 1.34 (0.89-1.97) | 1.09 | 6439.1 (4726.69-9022.25) | 8593.74 (5685.78-12640.03) | 0.43 (0.27-0.6) |
| Saint Lucia | 1.69 (1.29-2.25) | 3.36 (2.38-4.84) | 0.99 | 4949.34 (3770.12-6596.18) | 7365.89 (5204.11-10593.27) | 0.53 (0.25-0.81) |
| Saint Vincent and the Grenadines | 1.29 (0.98-1.76) | 1.92 (1.34-2.75) | 0.49 | 4882.67 (3724.12-6672.48) | 6898.72 (4816.29-9901.12) | 0.51 (0.27-0.75) |
| Samoa | 1.16 (0.9-1.54) | 1.62 (1.15-2.32) | 0.4 | 3152.18 (2452.64-4172.47) | 3334.01 (2363.09-4767.88) | -0.27 (-0.41--0.12) |
| San Marino | 0.51 (0.38-0.69) | 0.77 (0.52-1.1) | 0.51 | 8145.48 (6099.85-11055.98) | 10745.51 (7270.79-15483.25) | 0.35 (0.12-0.59) |
| Sao Tome and Principe | 1.29 (0.99-1.71) | 3.22 (2.29-4.49) | 1.5 | 5045.21 (3854.28-6676.2) | 5744.01 (4084.3-8000.51) | 0.25 (0.12-0.39) |
| Saudi Arabia | 235.55 (178.22-314.82) | 900.83 (612.63-1268.46) | 2.82 | 7223.15 (5465.24-9653.81) | 8878.5 (6038.06-12501.83) | 0.51 (0.32-0.69) |
| Senegal | 81.34 (62.79-111.2) | 231.58 (168.5-329.68) | 1.85 | 4726.95 (3649.36-6462.7) | 5936.48 (4319.5-8451.18) | 0.25 (0.03-0.48) |
| Serbia | 106.85 (86.79-135.39) | 105.73 (73.68-149.95) | -0.01 | 4576.11 (3716.84-5798.65) | 5201.56 (3624.47-7376.9) | -0.44 (-0.71--0.16) |
| Seychelles | 0.46 (0.35-0.61) | 0.84 (0.58-1.2) | 0.83 | 2534.22 (1937.35-3388.27) | 3461.11 (2379.71-4957.1) | 0.17 (-0.12-0.46) |
| Sierra Leone | 57.81 (44.49-76.17) | 144.46 (101.68-201.41) | 1.5 | 5758.23 (4430.95-7586.68) | 6351.92 (4471-8856.37) | 0.34 (0.26-0.43) |
| Singapore | 59.75 (50.51-70.92) | 54.18 (40.65-72.14) | -0.09 | 6393 (5403.88-7588.22) | 3724.54 (2794.52-4959.05) | -2.5 (-2.85--2.14) |
| Slovakia | 55.28 (43.7-70.33) | 71.21 (50.66-101.33) | 0.29 | 4167.42 (3294.66-5302.02) | 5641.62 (4013.58-8027.72) | -0.09 (-0.47-0.3) |
| Slovenia | 31.49 (25.75-38.59) | 26.16 (18.84-35.98) | -0.17 | 6329.11 (5175.35-7754.9) | 6219.71 (4479-8554.59) | -0.88 (-1.15--0.61) |
| Solomon Islands | 2.75 (2.12-3.7) | 6.66 (4.74-9.38) | 1.42 | 3701 (2846.72-4977.05) | 3884.9 (2764.54-5470.79) | -0.29 (-0.41--0.17) |
| Somalia | 113.8 (88.15-149.76) | 412.18 (290.04-570.08) | 2.62 | 6719.04 (5204.66-8841.96) | 8522.33 (5996.9-11787.25) | 0.05 (-0.15-0.26) |
| South Africa | 637.6 (536.86-773.08) | 1422.67 (1163.63-1761.87) | 1.23 | 6604.99 (5561.43-8008.37) | 9185.82 (7513.26-11375.93) | 0.6 (0.28-0.93) |
| South Sudan | 80.29 (62.37-107.48) | 162.24 (113.17-231.83) | 1.02 | 6199.79 (4815.86-8299.25) | 6988.11 (4874.38-9985.53) | 0.21 (0.09-0.33) |
| Spain | 758.09 (674.08-858.76) | 1216.22 (893.06-1631.61) | 0.6 | 7871.62 (6999.31-8916.86) | 12281.1 (9017.85-16475.53) | 1.6 (1.18-2.03) |
| Sri Lanka | 214.39 (174.12-264.56) | 241.85 (178.84-330.97) | 0.13 | 4669.95 (3792.78-5762.85) | 4282.67 (3166.94-5860.82) | -1.34 (-1.76--0.92) |
| Sudan | 367.68 (291.72-496.51) | 982.41 (687.56-1397.85) | 1.67 | 7853.29 (6230.89-10604.85) | 8702.36 (6090.53-12382.46) | -0.19 (-0.38-0.01) |
| Suriname | 7.98 (6.26-10.15) | 17.98 (13.07-24.54) | 1.25 | 8207.87 (6432.51-10442.43) | 12386.48 (9007.58-16907.2) | 0.46 (0.22-0.71) |
| Sweden | 200.61 (168.14-237.9) | 257.76 (200.52-325.86) | 0.28 | 9772.45 (8190.91-11589.13) | 11732.46 (9127.13-14832.11) | 0.06 (-0.12-0.25) |
| Switzerland | 159.43 (129.22-193.76) | 178.19 (130.4-244.13) | 0.12 | 9057.19 (7340.97-11007.6) | 9107.33 (6664.77-12477.49) | -0.81 (-1.33--0.28) |
| Syrian Arab Republic | 194.8 (148.37-261.8) | 342.24 (240.34-499.87) | 0.76 | 7064.37 (5380.76-9494.27) | 8796.83 (6177.66-12848.68) | 0.3 (0.08-0.51) |
| Taiwan (Province of China) | 118.4 (94.14-154.01) | 160.57 (116.96-217.91) | 0.36 | 2151.87 (1710.94-2799.1) | 2858.38 (2082.01-3879.08) | 0.86 (0.79-0.93) |
| Tajikistan | 46.96 (36.31-60.96) | 124.44 (88.33-174.09) | 1.65 | 3895.38 (3011.85-5056.39) | 4896.51 (3475.64-6850.3) | -0.01 (-0.28-0.26) |
| Thailand | 519.04 (413.58-676.07) | 590.24 (414.02-834.03) | 0.14 | 3245.99 (2586.45-4228.06) | 3620.76 (2539.76-5116.31) | -0.18 (-0.31--0.04) |
| Timor-Leste | 6.82 (5.3-9.01) | 12.65 (8.7-17.73) | 0.85 | 3659.07 (2844.53-4834.26) | 3652.76 (2514.21-5121.45) | -0.8 (-1.06--0.53) |
| Togo | 50.4 (39.53-65.39) | 140.35 (94.69-192.84) | 1.78 | 5906.37 (4632.88-7663.47) | 6508.59 (4391.26-8942.9) | 0.14 (0.04-0.23) |
| Tokelau | 0.01 (0.01-0.02) | 0.01 (0.01-0.02) | 0 | 3660.59 (2730.85-4941.07) | 4274.74 (2851.72-6102.95) | 0.13 (0.03-0.23) |
| Tonga | 0.67 (0.53-0.88) | 0.82 (0.57-1.17) | 0.22 | 2998.92 (2354.34-3924.2) | 3254.32 (2245.38-4623.93) | -0.16 (-0.3--0.03) |
| Trinidad and Tobago | 22.09 (17.34-27.47) | 31.9 (22.24-44.01) | 0.44 | 7180.58 (5636.45-8929.41) | 9386.48 (6544.47-12948.13) | -0.1 (-0.4-0.2) |
| Tunisia | 183 (144.04-242.27) | 385.16 (270.31-541.82) | 1.1 | 8950.16 (7044.69-11849.07) | 12553.48 (8810.15-17659.44) | 0.31 (0.02-0.6) |
| T眉rkiye | 1105.43 (959.43-1276.23) | 2084.66 (1469.98-2971.68) | 0.89 | 7760.55 (6735.55-8959.67) | 9628.22 (6789.25-13725.02) | 0.28 (0.1-0.47) |
| Turkmenistan | 37.32 (29.24-47.38) | 63.8 (45.1-87.27) | 0.71 | 4222.1 (3308.61-5360.4) | 5076.95 (3589.43-6944.79) | 0 (-0.21-0.21) |
| Tuvalu | 0.09 (0.07-0.12) | 0.12 (0.09-0.18) | 0.33 | 3644.58 (2681.83-5007.86) | 4244.62 (2964.8-6199) | 0.13 (0.04-0.23) |
| Uganda | 367.91 (285.12-486.92) | 1121.07 (797.47-1573.18) | 2.05 | 9561.18 (7409.56-12653.92) | 10765.37 (7657.91-15106.87) | -0.58 (-0.94--0.22) |
| Ukraine | 846.23 (677.19-1060.96) | 870.28 (629.16-1181.45) | 0.03 | 6685.08 (5349.66-8381.42) | 8612.14 (6226.05-11691.49) | -0.38 (-0.66--0.1) |
| United Arab Emirates | 25.35 (19.62-34.18) | 150.92 (105.5-215.08) | 4.95 | 7405.49 (5731.47-9986.7) | 8754.36 (6119.65-12475.78) | -0.05 (-0.32-0.22) |
| United Kingdom | 1288.47 (1055.65-1594.09) | 1550.53 (1254.52-1927.6) | 0.2 | 9079.12 (7438.56-11232.7) | 10108.08 (8178.34-12566.25) | -0.12 (-0.5-0.26) |
| United Republic of Tanzania | 371.46 (286.39-490.91) | 1035.76 (720.77-1471.59) | 1.79 | 6190.83 (4773.07-8181.5) | 6970.34 (4850.6-9903.35) | -0.06 (-0.2-0.09) |
| United States of America | 4521.77 (3835.62-5433.95) | 9475.73 (8050.83-11232.47) | 1.1 | 6749.45 (5725.26-8111.01) | 12515.14 (10633.19-14835.36) | 0.92 (0.55-1.3) |
| United States Virgin Islands | 1.5 (1.13-1.96) | 1.17 (0.83-1.61) | -0.22 | 5211.56 (3948.31-6827.01) | 6779.84 (4853.83-9353.75) | 0.37 (0.16-0.58) |
| Uruguay | 42.99 (33.98-56.54) | 72.64 (52.67-98.18) | 0.69 | 5724.85 (4524.96-7528.23) | 8726.71 (6327.31-11794.97) | 0.98 (0.75-1.21) |
| Uzbekistan | 214.35 (167.53-280.42) | 451.54 (320.59-623.51) | 1.11 | 4375.7 (3419.91-5724.29) | 5074.29 (3602.65-7006.83) | -0.02 (-0.2-0.15) |
| Vanuatu | 1.27 (0.98-1.67) | 3 (2.14-4.19) | 1.36 | 3624.07 (2788.16-4764.17) | 3823.55 (2733.21-5339.98) | -0.27 (-0.4--0.14) |
| Venezuela (Bolivarian Republic of) | 241.55 (185.13-323.93) | 410.22 (289.61-569.36) | 0.7 | 4990.24 (3824.54-6692.04) | 5976.57 (4219.35-8295.19) | 0.22 (0.08-0.36) |
| Viet Nam | 510.19 (402.96-643.14) | 829.17 (584.81-1142.66) | 0.63 | 2989.82 (2361.4-3768.91) | 3246.35 (2289.64-4473.72) | -0.46 (-0.66--0.27) |
| Yemen | 266.51 (207.45-348.26) | 809.76 (578.06-1163.54) | 2.04 | 9729.81 (7573.52-12714.26) | 9691.13 (6918.19-13925.13) | -0.12 (-0.2--0.05) |
| Zambia | 90.67 (69.54-122.99) | 295.48 (209.98-434.49) | 2.26 | 4929.98 (3781.21-6687.73) | 6006.97 (4268.95-8833.18) | -0.06 (-0.24-0.13) |
| Zimbabwe | 84.91 (64.92-114.17) | 188.48 (130.95-259.2) | 1.22 | 3531.33 (2699.84-4748.44) | 4641.65 (3224.93-6383.35) | 0.32 (0.07-0.57) |
