# Supplementary material for: Global burden of pancreatitis among individuals aged 15–39 years: a systematic analysis from the 2021 GBD study
Source: Front Med (Lausanne). 2025 May 27;12:1572346. doi: 10.3389/fmed.2025.1572346 (PMC12150401; doi:10.3389/fmed.2025.1572346)
Supplement: Supplementary file 7 [file Supplementary_file_7.docx]

**Supplementary Table 7** The death of pancreatitis cases and rates among aged 15-39 years in 1990 and 2021 across 204 countries, and the trends from 1990 to 2021.

| **location** | **Death cases** | | | **Death rates** | | |
| --- | --- | --- | --- | --- | --- | --- |
|  | **1990 thousand**  **(95%UI)** | **2021 thousand**  **(95%UI)** | **percentage**  **Change**  **(100%)** | **1990 per**  **(95%UI)** | **2021 per**  **(95%UI)** | **EAPC**  **(95% CI)** |
| Afghanistan | 3.04 (1.28-8.05) | 14.28 (6.33-28.1) | 3.7 | 0.1 (0.04-0.26) | 0.12 (0.05-0.23) | 1.07 (0.71-1.42) |
| Albania | 4.79 (3.41-6.67) | 2.4 (1.46-3.59) | -0.5 | 0.34 (0.24-0.47) | 0.25 (0.15-0.38) | -1.21 (-1.48--0.94) |
| Algeria | 6.67 (3.75-13.27) | 13.95 (8.96-22.32) | 1.09 | 0.07 (0.04-0.13) | 0.08 (0.05-0.13) | 0.92 (0.84-1) |
| American Samoa | 0.11 (0.07-0.2) | 0.13 (0.08-0.18) | 0.18 | 0.53 (0.33-0.97) | 0.72 (0.47-1.02) | 1.33 (0.74-1.93) |
| Andorra | 0.05 (0.03-0.08) | 0.04 (0.02-0.06) | -0.2 | 0.21 (0.13-0.31) | 0.15 (0.09-0.22) | -0.6 (-0.95--0.25) |
| Angola | 12.85 (6.52-22.79) | 41.94 (23.9-70.7) | 2.26 | 0.33 (0.17-0.58) | 0.34 (0.2-0.58) | 0.5 (0.26-0.73) |
| Antigua and Barbuda | 0.09 (0.07-0.1) | 0.08 (0.06-0.11) | -0.11 | 0.34 (0.29-0.4) | 0.22 (0.17-0.32) | -0.34 (-0.82-0.15) |
| Argentina | 94.59 (84.8-105.31) | 81.91 (73.71-89.93) | -0.13 | 0.77 (0.69-0.86) | 0.47 (0.42-0.51) | -1.2 (-1.65--0.76) |
| Armenia | 2.77 (2.08-3.26) | 2.34 (1.93-2.77) | -0.16 | 0.19 (0.14-0.23) | 0.22 (0.18-0.26) | 0.1 (-0.53-0.73) |
| Australia | 10.72 (9.7-11.96) | 9.63 (8.17-11.24) | -0.1 | 0.16 (0.14-0.18) | 0.11 (0.09-0.13) | -1.15 (-1.44--0.86) |
| Austria | 11.62 (10.33-13.09) | 4.36 (3.85-4.9) | -0.62 | 0.39 (0.34-0.44) | 0.15 (0.14-0.17) | -3.14 (-3.35--2.93) |
| Azerbaijan | 12.9 (8.34-19.45) | 17.09 (9.67-29.22) | 0.32 | 0.41 (0.26-0.61) | 0.4 (0.23-0.69) | -0.43 (-0.78--0.08) |
| Bahamas | 1.13 (0.97-1.31) | 1.43 (1.13-1.82) | 0.27 | 0.96 (0.82-1.11) | 0.92 (0.73-1.18) | -0.21 (-0.41--0.01) |
| Bahrain | 0.41 (0.3-0.59) | 1.04 (0.73-1.37) | 1.54 | 0.16 (0.12-0.23) | 0.15 (0.1-0.19) | -0.26 (-0.4--0.11) |
| Bangladesh | 413.64 (293.92-566.92) | 532.26 (312.4-803.83) | 0.29 | 0.98 (0.7-1.34) | 0.77 (0.45-1.17) | -0.78 (-0.89--0.67) |
| Barbados | 0.59 (0.52-0.68) | 0.38 (0.29-0.49) | -0.36 | 0.54 (0.47-0.63) | 0.38 (0.3-0.5) | -1.44 (-1.71--1.16) |
| Belarus | 79.31 (65.54-90.94) | 85.71 (68.35-105.08) | 0.08 | 2.01 (1.66-2.31) | 2.92 (2.33-3.58) | 0.47 (-0.09-1.03) |
| Belgium | 11.48 (10.23-12.81) | 5.5 (4.77-6.22) | -0.52 | 0.31 (0.27-0.34) | 0.16 (0.14-0.18) | -2.58 (-2.84--2.32) |
| Belize | 0.19 (0.14-0.28) | 0.67 (0.58-0.76) | 2.53 | 0.26 (0.19-0.38) | 0.35 (0.31-0.4) | 1.17 (0.63-1.71) |
| Benin | 15.09 (8.52-26.67) | 49.06 (29.61-78.82) | 2.25 | 0.89 (0.5-1.57) | 0.94 (0.56-1.5) | 0.12 (0.03-0.21) |
| Bermuda | 0.13 (0.11-0.15) | 0.05 (0.04-0.06) | -0.62 | 0.5 (0.43-0.58) | 0.28 (0.23-0.36) | -1.82 (-2.11--1.54) |
| Bhutan | 3.37 (1.57-5.93) | 3.92 (2.07-7.26) | 0.16 | 1.25 (0.58-2.2) | 1.13 (0.6-2.09) | -0.45 (-0.5--0.39) |
| Bolivia (Plurinational State of) | 31.62 (19.56-46.27) | 47.11 (29.87-68.63) | 0.49 | 1.28 (0.79-1.88) | 0.96 (0.61-1.4) | -1.26 (-1.44--1.08) |
| Bosnia and Herzegovina | 16.08 (11.93-22.19) | 5.37 (3.42-7.71) | -0.67 | 0.85 (0.63-1.17) | 0.53 (0.34-0.77) | -1.72 (-1.95--1.49) |
| Botswana | 2.97 (1.04-7.52) | 4.75 (2.14-9.51) | 0.6 | 0.58 (0.2-1.46) | 0.45 (0.2-0.89) | -0.89 (-1.09--0.69) |
| Brazil | 632.49 (603-664.28) | 792.76 (748.93-839.08) | 0.25 | 1.01 (0.96-1.06) | 0.93 (0.88-0.98) | -0.14 (-0.39-0.11) |
| Brunei Darussalam | 0.36 (0.25-0.5) | 0.54 (0.38-0.73) | 0.5 | 0.29 (0.2-0.4) | 0.26 (0.19-0.36) | -0.48 (-0.76--0.2) |
| Bulgaria | 23.67 (21.27-26.34) | 16.94 (13.3-20.61) | -0.28 | 0.8 (0.71-0.88) | 0.89 (0.7-1.09) | 0.42 (0.18-0.66) |
| Burkina Faso | 17.5 (9.72-28.73) | 63.15 (36.85-103.13) | 2.61 | 0.55 (0.31-0.9) | 0.73 (0.43-1.19) | 1.02 (0.81-1.24) |
| Burundi | 7.16 (3.39-15.54) | 17.8 (7.72-34.29) | 1.49 | 0.35 (0.16-0.75) | 0.34 (0.15-0.65) | -0.34 (-0.58--0.09) |
| Cabo Verde | 0.9 (0.5-1.51) | 2.16 (1.34-3.45) | 1.4 | 0.69 (0.39-1.16) | 0.86 (0.54-1.38) | 0.34 (0.11-0.57) |
| Cambodia | 33.56 (20.26-61) | 66.84 (37.8-116.58) | 0.99 | 0.87 (0.53-1.58) | 0.92 (0.52-1.61) | -0.03 (-0.12-0.07) |
| Cameroon | 32.75 (18.15-54.9) | 132.08 (71.68-233.12) | 3.03 | 0.86 (0.48-1.44) | 1.02 (0.56-1.81) | 0.65 (0.51-0.8) |
| Canada | 24.73 (22.59-27.18) | 31.08 (27.43-35.52) | 0.26 | 0.22 (0.2-0.24) | 0.26 (0.23-0.3) | 0.06 (-0.26-0.37) |
| Central African Republic | 4.39 (2.43-7.38) | 8.99 (4.49-15.09) | 1.05 | 0.42 (0.23-0.71) | 0.41 (0.21-0.69) | -0.08 (-0.18-0.02) |
| Chad | 13.32 (5.57-27.79) | 50.83 (25.82-91.49) | 2.82 | 0.63 (0.27-1.32) | 0.81 (0.41-1.45) | 1.01 (0.87-1.15) |
| Chile | 36.59 (33.58-39.6) | 28.37 (24.92-31.65) | -0.22 | 0.64 (0.59-0.69) | 0.4 (0.35-0.45) | -1.45 (-1.73--1.18) |
| China | 1537.85 (1223.44-1872.26) | 941.05 (705.61-1269.86) | -0.39 | 0.28 (0.22-0.34) | 0.2 (0.15-0.28) | -1.32 (-1.49--1.15) |
| Colombia | 70.11 (65.27-75.92) | 71.47 (60.14-83.59) | 0.02 | 0.5 (0.46-0.54) | 0.36 (0.3-0.42) | -1 (-1.29--0.71) |
| Comoros | 0.61 (0.29-1.26) | 1.24 (0.63-2) | 1.03 | 0.35 (0.17-0.73) | 0.4 (0.2-0.65) | -0.12 (-0.63-0.39) |
| Congo | 3.66 (1.63-7.22) | 10.02 (5.62-17.06) | 1.74 | 0.39 (0.17-0.76) | 0.45 (0.25-0.77) | 0.75 (0.55-0.95) |
| Cook Islands | 0.1 (0.06-0.16) | 0.06 (0.04-0.08) | -0.4 | 1.29 (0.81-2.02) | 0.95 (0.61-1.41) | -0.83 (-0.99--0.67) |
| Costa Rica | 9.53 (8.49-10.66) | 16.43 (14.64-18.51) | 0.72 | 0.74 (0.66-0.83) | 0.86 (0.77-0.97) | 0.01 (-0.3-0.33) |
| C么te d'Ivoire | 42.56 (27.08-65.96) | 119.36 (77.23-176.68) | 1.8 | 0.9 (0.57-1.39) | 1.06 (0.69-1.58) | 0.64 (0.46-0.81) |
| Croatia | 17.99 (16.04-20.37) | 5.23 (4.18-6.37) | -0.71 | 0.99 (0.88-1.12) | 0.42 (0.33-0.51) | -2.97 (-3.16--2.77) |
| Cuba | 17.83 (15.2-20.15) | 12.21 (10.27-14.25) | -0.32 | 0.37 (0.31-0.41) | 0.34 (0.29-0.4) | -0.71 (-1.07--0.35) |
| Cyprus | 1.01 (0.74-1.38) | 1.02 (0.72-1.49) | 0.01 | 0.33 (0.24-0.45) | 0.2 (0.14-0.3) | -1.91 (-2.15--1.67) |
| Czechia | 45.41 (39.9-51.85) | 21.55 (17.4-26.75) | -0.53 | 1.22 (1.08-1.4) | 0.73 (0.59-0.91) | -1.46 (-1.81--1.11) |
| Democratic People's Republic of Korea | 24.1 (12.46-40.86) | 27.35 (11.22-57.96) | 0.13 | 0.29 (0.15-0.49) | 0.27 (0.11-0.58) | -0.46 (-0.55--0.37) |
| Democratic Republic of the Congo | 57.61 (30.79-109.11) | 152.23 (79.62-275.19) | 1.64 | 0.4 (0.21-0.76) | 0.42 (0.22-0.76) | 0.29 (0.13-0.45) |
| Denmark | 7.77 (6.79-8.86) | 3.39 (2.96-3.85) | -0.56 | 0.41 (0.36-0.46) | 0.19 (0.16-0.21) | -3.01 (-3.43--2.59) |
| Djibouti | 0.62 (0.24-1.4) | 2.3 (1.01-4.25) | 2.71 | 0.35 (0.14-0.8) | 0.43 (0.19-0.79) | 0.45 (0.27-0.63) |
| Dominica | 0.12 (0.08-0.16) | 0.13 (0.09-0.17) | 0.08 | 0.39 (0.28-0.54) | 0.49 (0.34-0.66) | 0.86 (0.65-1.07) |
| Dominican Republic | 10.54 (7.86-13.73) | 14.15 (8.22-20.7) | 0.34 | 0.34 (0.26-0.45) | 0.31 (0.18-0.46) | 0.18 (-0.12-0.49) |
| Ecuador | 55.67 (49.96-61.61) | 49.6 (40.12-61.41) | -0.11 | 1.35 (1.21-1.49) | 0.68 (0.55-0.84) | -1.97 (-2.27--1.68) |
| Egypt | 13.17 (8.58-17.19) | 21.86 (14.83-30.65) | 0.66 | 0.06 (0.04-0.08) | 0.05 (0.04-0.07) | -1.61 (-2.17--1.05) |
| El Salvador | 21.06 (15.04-26.05) | 19.47 (12.95-28.62) | -0.08 | 1.01 (0.72-1.25) | 0.75 (0.5-1.1) | -0.79 (-0.97--0.6) |
| Equatorial Guinea | 0.79 (0.37-1.98) | 2.91 (1.41-5.81) | 2.68 | 0.52 (0.24-1.31) | 0.42 (0.2-0.83) | -0.74 (-1--0.47) |
| Eritrea | 4.88 (2.5-7.99) | 11.88 (6.29-21.64) | 1.43 | 0.38 (0.19-0.62) | 0.42 (0.22-0.77) | 0.34 (0.26-0.42) |
| Estonia | 8.3 (6.81-10.63) | 4.53 (3.64-5.46) | -0.45 | 1.46 (1.2-1.87) | 1.15 (0.92-1.38) | -0.82 (-1.2--0.45) |
| Eswatini | 1.4 (0.73-2.97) | 3.03 (1.57-4.93) | 1.16 | 0.46 (0.24-0.98) | 0.59 (0.31-0.97) | 0.82 (0.61-1.03) |
| Ethiopia | 45.18 (29.36-74.8) | 125.5 (56.18-216.47) | 1.78 | 0.25 (0.16-0.41) | 0.27 (0.12-0.47) | 0.19 (0.13-0.26) |
| Fiji | 1.44 (0.88-2.1) | 1.04 (0.68-1.59) | -0.28 | 0.45 (0.27-0.65) | 0.29 (0.19-0.45) | -1.71 (-1.89--1.52) |
| Finland | 14.75 (11.96-17.81) | 6.31 (5.48-7.16) | -0.57 | 0.81 (0.66-0.98) | 0.38 (0.33-0.43) | -2.1 (-2.28--1.92) |
| France | 75.36 (67.91-83.79) | 35.46 (30.85-40.66) | -0.53 | 0.34 (0.31-0.38) | 0.18 (0.16-0.2) | -1.87 (-2.1--1.64) |
| Gabon | 1.23 (0.72-2.05) | 2.77 (1.53-4.73) | 1.25 | 0.32 (0.19-0.53) | 0.37 (0.2-0.63) | 0.39 (0.27-0.51) |
| Gambia | 4.1 (2.33-7.52) | 13.97 (8.32-23.12) | 2.41 | 1.09 (0.62-2) | 1.4 (0.83-2.31) | 0.5 (0.22-0.78) |
| Georgia | 1.1 (0.85-1.39) | 5.32 (3.66-7.39) | 3.84 | 0.05 (0.04-0.07) | 0.47 (0.32-0.65) | 8.61 (7.56-9.68) |
| Germany | 157.5 (139.25-178.5) | 54.33 (46.74-62.01) | -0.66 | 0.53 (0.47-0.6) | 0.21 (0.18-0.25) | -3.59 (-3.92--3.25) |
| Ghana | 36.02 (23-54.66) | 112.62 (70.31-181.14) | 2.13 | 0.63 (0.4-0.95) | 0.79 (0.49-1.27) | 0.64 (0.53-0.74) |
| Greece | 10.14 (9.13-11.3) | 6.81 (5.96-7.67) | -0.33 | 0.27 (0.24-0.3) | 0.24 (0.21-0.28) | 0.07 (-0.22-0.37) |
| Greenland | 0.19 (0.13-0.27) | 0.09 (0.06-0.14) | -0.53 | 0.73 (0.48-1.03) | 0.44 (0.28-0.71) | -2.63 (-3.08--2.18) |
| Grenada | 0.2 (0.16-0.24) | 0.17 (0.14-0.2) | -0.15 | 0.6 (0.49-0.73) | 0.41 (0.34-0.5) | -1.33 (-1.55--1.1) |
| Guam | 0.1 (0.06-0.15) | 0.07 (0.05-0.1) | -0.3 | 0.15 (0.1-0.23) | 0.13 (0.08-0.18) | 0.34 (-0.24-0.91) |
| Guatemala | 71.7 (63.35-80.1) | 120.89 (97.54-142.53) | 0.69 | 2.43 (2.14-2.71) | 1.78 (1.43-2.09) | -0.87 (-1.21--0.54) |
| Guinea | 16.06 (7.4-31.97) | 42.52 (24.18-69.36) | 1.65 | 0.78 (0.36-1.56) | 0.82 (0.47-1.34) | 0 (-0.06-0.07) |
| Guinea-Bissau | 4.2 (2.48-6.67) | 11.35 (6.61-18.53) | 1.7 | 1.13 (0.67-1.8) | 1.34 (0.78-2.2) | 0.76 (0.68-0.85) |
| Guyana | 1.71 (1.41-2.02) | 3.13 (2.31-4.01) | 0.83 | 0.5 (0.41-0.59) | 1.01 (0.74-1.29) | 2.33 (1.5-3.15) |
| Haiti | 17.84 (10.84-27.65) | 35.68 (19.7-55.37) | 1 | 0.73 (0.45-1.14) | 0.65 (0.36-1.01) | -0.11 (-0.3-0.07) |
| Honduras | 24.48 (18.37-32.35) | 41.12 (21.7-70.17) | 0.68 | 1.42 (1.06-1.87) | 0.94 (0.49-1.6) | -1.62 (-1.74--1.5) |
| Hungary | 86.56 (77.15-95.5) | 18.43 (15.46-21.49) | -0.79 | 2.34 (2.09-2.58) | 0.67 (0.56-0.78) | -4.49 (-5.05--3.93) |
| Iceland | 0.29 (0.26-0.32) | 0.28 (0.24-0.31) | -0.03 | 0.28 (0.25-0.31) | 0.23 (0.2-0.26) | -0.14 (-0.51-0.24) |
| India | 3376.5 (2565.99-5250.57) | 4096.55 (3096.51-5162.31) | 0.21 | 0.99 (0.75-1.54) | 0.67 (0.51-0.85) | -1.21 (-1.46--0.96) |
| Indonesia | 486.95 (340.57-779.31) | 641.33 (424.42-1074.36) | 0.32 | 0.62 (0.44-1) | 0.56 (0.37-0.94) | -0.27 (-0.36--0.19) |
| Iran (Islamic Republic of) | 10.02 (6.67-13.25) | 19.42 (13.47-23.35) | 0.94 | 0.05 (0.03-0.06) | 0.06 (0.04-0.07) | 1.53 (1.08-1.97) |
| Iraq | 7.4 (4.54-10.23) | 12.04 (7.62-18.19) | 0.63 | 0.1 (0.06-0.14) | 0.07 (0.04-0.1) | -1.08 (-1.32--0.84) |
| Ireland | 2.3 (2.04-2.58) | 1.85 (1.58-2.18) | -0.2 | 0.17 (0.15-0.19) | 0.12 (0.1-0.14) | -1.05 (-1.61--0.5) |
| Israel | 3.95 (3.48-4.59) | 4.21 (3.68-4.74) | 0.07 | 0.21 (0.18-0.24) | 0.13 (0.11-0.14) | -1.41 (-1.69--1.13) |
| Italy | 49.13 (47.39-50.95) | 16.65 (15.86-17.46) | -0.66 | 0.23 (0.22-0.24) | 0.11 (0.1-0.11) | -2.48 (-2.71--2.24) |
| Jamaica | 1.47 (1.08-2.17) | 1.91 (1.25-2.6) | 0.3 | 0.15 (0.11-0.22) | 0.16 (0.1-0.22) | -0.83 (-1.4--0.25) |
| Japan | 83.54 (81.52-85.7) | 34.42 (32.56-36.36) | -0.59 | 0.19 (0.18-0.19) | 0.11 (0.1-0.11) | -1.92 (-2.07--1.77) |
| Jordan | 2.15 (1.49-2.95) | 5.18 (3.74-7.33) | 1.41 | 0.14 (0.1-0.19) | 0.1 (0.07-0.14) | -1.26 (-1.51--1) |
| Kazakhstan | 140.84 (116.68-164.63) | 152.69 (113.65-206.89) | 0.08 | 2.07 (1.72-2.42) | 2.19 (1.63-2.97) | -0.73 (-1.36--0.09) |
| Kenya | 26.01 (10.66-55.63) | 73.54 (40.25-124.46) | 1.83 | 0.3 (0.12-0.64) | 0.34 (0.19-0.57) | 0.61 (0.51-0.71) |
| Kiribati | 0.43 (0.25-0.66) | 0.63 (0.35-0.98) | 0.47 | 1.42 (0.82-2.15) | 1.27 (0.7-1.98) | -0.43 (-0.48--0.37) |
| Kuwait | 1.28 (1.09-1.5) | 2.56 (2.02-3.2) | 1 | 0.15 (0.13-0.18) | 0.12 (0.1-0.15) | -0.31 (-1.09-0.48) |
| Kyrgyzstan | 6.8 (5.9-7.81) | 24.79 (19.07-31.73) | 2.65 | 0.38 (0.33-0.43) | 0.91 (0.7-1.17) | 2.94 (2.42-3.47) |
| Lao People's Democratic Republic | 8.35 (5.08-13.52) | 16.36 (9.5-26.84) | 0.96 | 0.54 (0.33-0.87) | 0.51 (0.3-0.84) | -0.42 (-0.52--0.31) |
| Latvia | 16.24 (14.11-18.75) | 12.6 (10.58-14.87) | -0.22 | 1.7 (1.48-1.97) | 2.34 (1.96-2.76) | 0.41 (0.05-0.77) |
| Lebanon | 1.54 (0.77-2.56) | 2.4 (1.8-3.19) | 0.56 | 0.13 (0.07-0.22) | 0.1 (0.08-0.14) | -0.83 (-1.05--0.61) |
| Lesotho | 2.11 (0.65-5.49) | 4.22 (2.65-6.11) | 1 | 0.39 (0.12-1.02) | 0.51 (0.32-0.73) | 1.24 (1.04-1.43) |
| Liberia | 8.05 (4.93-13.17) | 22.28 (12.72-34.69) | 1.77 | 0.87 (0.53-1.43) | 0.99 (0.57-1.55) | 1.04 (0.67-1.41) |
| Libya | 1.44 (0.75-2.67) | 3.99 (2.58-6.29) | 1.77 | 0.09 (0.04-0.16) | 0.13 (0.09-0.21) | 2.25 (1.81-2.7) |
| Lithuania | 18.89 (15.37-24.18) | 18.79 (15.28-22.48) | -0.01 | 1.36 (1.1-1.74) | 2.33 (1.9-2.79) | 2.22 (1.65-2.8) |
| Luxembourg | 0.45 (0.4-0.52) | 0.22 (0.19-0.25) | -0.51 | 0.31 (0.27-0.35) | 0.1 (0.08-0.12) | -4.12 (-4.41--3.84) |
| Madagascar | 13.7 (7.11-22.33) | 41.28 (18.13-74.53) | 2.01 | 0.3 (0.16-0.49) | 0.35 (0.15-0.64) | 0.38 (0.19-0.58) |
| Malawi | 13.36 (7.05-22.97) | 32.74 (19.98-52.88) | 1.45 | 0.36 (0.19-0.61) | 0.4 (0.24-0.65) | 0.42 (0.27-0.57) |
| Malaysia | 41.05 (29.3-58.76) | 75.82 (51.12-117.5) | 0.85 | 0.55 (0.39-0.79) | 0.55 (0.37-0.85) | -0.84 (-1.12--0.57) |
| Maldives | 0.14 (0.07-0.25) | 0.57 (0.33-1.09) | 3.07 | 0.17 (0.09-0.3) | 0.22 (0.13-0.42) | 0.91 (0.59-1.24) |
| Mali | 29.29 (13.84-70.31) | 89.1 (46.21-189.45) | 2.04 | 0.98 (0.46-2.35) | 1 (0.52-2.13) | 0.24 (0.18-0.3) |
| Malta | 0.29 (0.26-0.33) | 0.22 (0.19-0.25) | -0.24 | 0.21 (0.19-0.24) | 0.17 (0.14-0.19) | -0.22 (-0.55-0.1) |
| Marshall Islands | 0.13 (0.08-0.2) | 0.14 (0.08-0.24) | 0.08 | 0.78 (0.46-1.17) | 0.61 (0.34-1) | -0.69 (-0.78--0.59) |
| Mauritania | 6.9 (3.36-11.81) | 14.03 (7.37-23.99) | 1.03 | 0.9 (0.44-1.54) | 0.82 (0.43-1.41) | -0.31 (-0.38--0.24) |
| Mauritius | 10.07 (8.88-11.41) | 7.2 (6.24-8.23) | -0.29 | 2.03 (1.79-2.3) | 1.58 (1.37-1.81) | -2.17 (-2.75--1.59) |
| Mexico | 327.47 (315.52-342.91) | 570.95 (516.93-626.38) | 0.74 | 0.92 (0.88-0.96) | 1.11 (1-1.22) | 0.45 (0.17-0.74) |
| Micronesia (Federated States of) | 0.32 (0.2-0.49) | 0.28 (0.15-0.47) | -0.12 | 0.8 (0.5-1.22) | 0.65 (0.36-1.11) | -0.67 (-0.72--0.62) |
| Monaco | 0.02 (0.01-0.03) | 0.02 (0.01-0.03) | 0 | 0.24 (0.16-0.36) | 0.23 (0.14-0.35) | -0.49 (-0.8--0.19) |
| Mongolia | 11.81 (8.15-17.03) | 18.78 (13.53-26.47) | 0.59 | 1.34 (0.92-1.93) | 1.49 (1.07-2.1) | -0.21 (-0.38--0.03) |
| Montenegro | 1.95 (1.43-2.57) | 1.32 (0.9-1.91) | -0.32 | 0.78 (0.57-1.03) | 0.64 (0.44-0.93) | -0.4 (-0.61--0.19) |
| Morocco | 7.99 (4.27-15.59) | 13.45 (8.29-23.94) | 0.68 | 0.08 (0.04-0.15) | 0.09 (0.06-0.16) | 0.7 (0.47-0.93) |
| Mozambique | 18.46 (8.48-33.54) | 71.88 (41.78-113.94) | 2.89 | 0.39 (0.18-0.71) | 0.6 (0.35-0.95) | 1.99 (1.82-2.16) |
| Myanmar | 190.61 (103.63-367.53) | 230.77 (124.99-444.94) | 0.21 | 1.11 (0.6-2.14) | 1.03 (0.56-1.98) | -0.41 (-0.51--0.31) |
| Namibia | 2.66 (0.92-7.01) | 5.2 (2.11-11.24) | 0.95 | 0.47 (0.16-1.25) | 0.5 (0.2-1.08) | -0.09 (-0.25-0.07) |
| Nauru | 0.03 (0.01-0.05) | 0.03 (0.01-0.05) | 0 | 0.73 (0.36-1.23) | 0.59 (0.24-1.09) | -0.83 (-1.01--0.65) |
| Nepal | 89.71 (55.11-138.64) | 115.92 (76.69-171.97) | 0.29 | 1.23 (0.75-1.9) | 0.86 (0.57-1.28) | -1.28 (-1.34--1.22) |
| Netherlands | 13.14 (11.82-14.64) | 6.92 (6.25-7.72) | -0.47 | 0.22 (0.2-0.24) | 0.13 (0.12-0.15) | -2.26 (-2.58--1.93) |
| New Zealand | 1.84 (1.67-2) | 1.56 (1.38-1.73) | -0.15 | 0.13 (0.12-0.14) | 0.09 (0.08-0.1) | -1.2 (-1.66--0.74) |
| Nicaragua | 9.35 (6.75-12.46) | 15.3 (10.84-20.18) | 0.64 | 0.63 (0.46-0.84) | 0.54 (0.38-0.71) | -0.17 (-0.35-0.02) |
| Niger | 23.56 (9.66-56.02) | 80.34 (29.46-189.59) | 2.41 | 0.85 (0.35-2.01) | 0.9 (0.33-2.13) | 0 (-0.13-0.14) |
| Nigeria | 232.11 (129.15-412.52) | 618.97 (355.64-962.19) | 1.67 | 0.68 (0.38-1.21) | 0.69 (0.4-1.07) | 0.2 (0.1-0.31) |
| Niue | 0 (0-0.01) | 0 (0-0) | NA | 0.6 (0.36-0.98) | 0.57 (0.4-0.83) | -0.62 (-0.78--0.46) |
| North Macedonia | 4.68 (3.56-6.33) | 3.11 (1.96-4.39) | -0.34 | 0.59 (0.45-0.8) | 0.41 (0.26-0.57) | -1.13 (-1.36--0.9) |
| Northern Mariana Islands | 0.06 (0.03-0.13) | 0.05 (0.03-0.07) | -0.17 | 0.28 (0.14-0.55) | 0.28 (0.19-0.4) | 0.54 (-0.31-1.4) |
| Norway | 1.88 (1.78-1.98) | 1.61 (1.49-1.72) | -0.14 | 0.12 (0.11-0.12) | 0.09 (0.08-0.1) | -1.28 (-1.74--0.82) |
| Oman | 0.63 (0.31-1.15) | 1.48 (0.83-2.81) | 1.35 | 0.08 (0.04-0.14) | 0.06 (0.04-0.12) | 0.46 (-0.14-1.06) |
| Pakistan | 284.84 (166.56-497.71) | 634.05 (413.18-895.83) | 1.23 | 0.7 (0.41-1.22) | 0.64 (0.42-0.91) | -0.76 (-0.9--0.62) |
| Palau | 0.04 (0.02-0.07) | 0.05 (0.03-0.08) | 0.25 | 0.62 (0.32-0.99) | 0.87 (0.52-1.42) | 1.06 (0.97-1.15) |
| Palestine | 1.21 (0.7-1.99) | 1.97 (1.41-2.73) | 0.63 | 0.16 (0.09-0.26) | 0.09 (0.06-0.12) | -2.06 (-2.21--1.91) |
| Panama | 3.48 (3.16-3.85) | 5.87 (4.62-7.03) | 0.69 | 0.34 (0.31-0.38) | 0.36 (0.28-0.43) | 0.41 (0.09-0.73) |
| Papua New Guinea | 5.38 (1.22-10.54) | 11.16 (4.41-20.47) | 1.07 | 0.32 (0.07-0.64) | 0.26 (0.1-0.48) | -1.11 (-1.38--0.84) |
| Paraguay | 11.19 (7.93-16.24) | 20.68 (15.12-28.02) | 0.85 | 0.71 (0.51-1.04) | 0.68 (0.49-0.92) | 0.31 (0.13-0.48) |
| Peru | 114.37 (84.62-149.91) | 114.82 (83.01-156.67) | 0 | 1.29 (0.95-1.69) | 0.77 (0.56-1.05) | -1.85 (-2.1--1.6) |
| Philippines | 97.91 (65.58-121.24) | 165.97 (122.19-227.3) | 0.7 | 0.38 (0.25-0.47) | 0.35 (0.26-0.48) | -0.42 (-0.51--0.33) |
| Poland | 223.6 (216.14-231.38) | 208.03 (190.93-225.63) | -0.07 | 1.55 (1.5-1.6) | 1.72 (1.58-1.86) | -0.04 (-0.33-0.26) |
| Portugal | 21.52 (19.29-24.14) | 6.19 (5.06-7.12) | -0.71 | 0.57 (0.51-0.64) | 0.21 (0.17-0.24) | -3.37 (-3.76--2.99) |
| Puerto Rico | 13.91 (12.61-15.31) | 4.76 (3.8-5.7) | -0.66 | 0.98 (0.89-1.08) | 0.46 (0.37-0.55) | -2.47 (-2.71--2.22) |
| Qatar | 0.41 (0.25-0.69) | 2.47 (1.42-4.13) | 5.02 | 0.17 (0.1-0.29) | 0.15 (0.09-0.25) | -0.12 (-0.41-0.18) |
| Republic of Korea | 73.45 (38.57-103.36) | 22.64 (16.44-31.68) | -0.69 | 0.35 (0.18-0.49) | 0.14 (0.1-0.2) | -3.48 (-3.77--3.2) |
| Republic of Moldova | 67.66 (59.25-78.2) | 35.6 (29.73-42.41) | -0.47 | 3.88 (3.4-4.49) | 2.87 (2.4-3.42) | -1.73 (-2.1--1.36) |
| Romania | 182.71 (157.9-208.7) | 70.37 (58.04-82.32) | -0.61 | 2.1 (1.82-2.4) | 1.31 (1.08-1.53) | -1.77 (-1.96--1.59) |
| Russian Federation | 813.77 (791.88-847.59) | 1827.14 (1598.7-1992.4) | 1.25 | 1.4 (1.36-1.46) | 3.93 (3.44-4.29) | 2.74 (1.85-3.64) |
| Rwanda | 11.29 (7.03-17.86) | 23.73 (9.83-45.74) | 1.1 | 0.41 (0.26-0.65) | 0.42 (0.17-0.81) | -0.2 (-0.61-0.22) |
| Saint Kitts and Nevis | 0.16 (0.13-0.19) | 0.13 (0.08-0.24) | -0.19 | 0.92 (0.76-1.1) | 0.59 (0.36-1.07) | -2.54 (-3.17--1.91) |
| Saint Lucia | 0.11 (0.1-0.13) | 0.14 (0.11-0.17) | 0.27 | 0.2 (0.17-0.23) | 0.21 (0.17-0.26) | 0.31 (0.02-0.6) |
| Saint Vincent and the Grenadines | 0.23 (0.21-0.26) | 0.23 (0.19-0.27) | 0 | 0.51 (0.45-0.58) | 0.56 (0.47-0.66) | 0.02 (-0.29-0.33) |
| Samoa | 0.37 (0.22-0.65) | 0.38 (0.22-0.62) | 0.03 | 0.55 (0.33-0.96) | 0.47 (0.27-0.78) | -0.57 (-0.63--0.5) |
| San Marino | 0.01 (0.01-0.01) | 0.01 (0-0.01) | 0 | 0.1 (0.07-0.13) | 0.06 (0.03-0.1) | -0.49 (-0.97-0) |
| Sao Tome and Principe | 0.29 (0.16-0.48) | 0.83 (0.43-1.47) | 1.86 | 0.68 (0.37-1.11) | 0.91 (0.47-1.61) | 0.97 (0.81-1.13) |
| Saudi Arabia | 11.19 (5.9-19.86) | 38.7 (20.22-62.22) | 2.46 | 0.17 (0.09-0.3) | 0.21 (0.11-0.34) | 1.34 (1.01-1.67) |
| Senegal | 29.92 (16.79-54.7) | 71.68 (41.98-118.6) | 1.4 | 1.08 (0.61-1.98) | 1.11 (0.65-1.84) | 0.36 (0.18-0.54) |
| Serbia | 42.35 (31.37-56.28) | 19.07 (14.51-25.4) | -0.55 | 1.18 (0.87-1.57) | 0.64 (0.49-0.86) | -1.81 (-1.89--1.72) |
| Seychelles | 0.17 (0.11-0.3) | 0.18 (0.11-0.32) | 0.06 | 0.54 (0.35-0.95) | 0.46 (0.29-0.82) | -0.44 (-0.69--0.19) |
| Sierra Leone | 11.72 (5.76-21.8) | 33.51 (18.89-55.57) | 1.86 | 0.73 (0.36-1.36) | 0.9 (0.51-1.49) | 0.77 (0.5-1.04) |
| Singapore | 3.32 (3-3.67) | 1.26 (1.12-1.41) | -0.62 | 0.22 (0.2-0.24) | 0.07 (0.06-0.07) | -4.64 (-5--4.28) |
| Slovakia | 31.07 (22.7-41.51) | 18.65 (12.97-24.67) | -0.4 | 1.52 (1.11-2.03) | 1.09 (0.76-1.44) | -0.62 (-0.82--0.41) |
| Slovenia | 7.37 (6.45-8.66) | 2.03 (1.68-2.39) | -0.72 | 0.96 (0.84-1.13) | 0.36 (0.29-0.42) | -3.31 (-3.54--3.07) |
| Solomon Islands | 0.46 (0.16-0.87) | 0.95 (0.57-1.48) | 1.07 | 0.36 (0.13-0.68) | 0.35 (0.21-0.54) | -0.12 (-0.21--0.02) |
| Somalia | 11.28 (5.43-22.08) | 29.05 (12.55-64.37) | 1.58 | 0.39 (0.19-0.76) | 0.35 (0.15-0.78) | 0.03 (-0.18-0.24) |
| South Africa | 69.23 (50.97-84.37) | 94.41 (66.02-129.14) | 0.36 | 0.44 (0.32-0.54) | 0.39 (0.27-0.53) | -0.14 (-0.64-0.36) |
| South Sudan | 7.79 (3.36-16.97) | 12.6 (7.04-21.06) | 0.62 | 0.34 (0.15-0.74) | 0.35 (0.2-0.59) | 0.13 (-0.05-0.32) |
| Spain | 82.67 (76.27-90.38) | 25.58 (22.57-28.93) | -0.69 | 0.56 (0.51-0.61) | 0.21 (0.18-0.23) | -3.49 (-3.63--3.35) |
| Sri Lanka | 24.29 (15.51-35.02) | 14.42 (8.68-22.56) | -0.41 | 0.33 (0.21-0.47) | 0.18 (0.11-0.28) | -2.25 (-2.42--2.08) |
| Sudan | 5.45 (2.86-9.5) | 18.56 (10.69-31.94) | 2.41 | 0.07 (0.04-0.12) | 0.1 (0.06-0.17) | 1.54 (1.33-1.75) |
| Suriname | 1.15 (0.79-1.52) | 1.52 (1.05-2.13) | 0.32 | 0.7 (0.49-0.93) | 0.71 (0.49-0.99) | -0.15 (-0.3-0.01) |
| Sweden | 7.24 (6.48-8.16) | 4.55 (3.95-5.25) | -0.37 | 0.25 (0.22-0.28) | 0.14 (0.12-0.16) | -1.27 (-1.67--0.86) |
| Switzerland | 5.9 (4.91-6.82) | 2.3 (1.93-2.63) | -0.61 | 0.22 (0.19-0.26) | 0.08 (0.07-0.09) | -3.66 (-3.86--3.46) |
| Syrian Arab Republic | 4.3 (2.97-5.83) | 2.95 (1.87-4.25) | -0.31 | 0.09 (0.06-0.12) | 0.06 (0.04-0.08) | -0.99 (-1.17--0.81) |
| Taiwan (Province of China) | 46.81 (42.13-51.63) | 22.76 (19.42-26.8) | -0.51 | 0.51 (0.46-0.56) | 0.3 (0.26-0.36) | -2.79 (-3.43--2.15) |
| Tajikistan | 6.53 (3.77-9.95) | 15.21 (9.61-23.72) | 1.33 | 0.31 (0.18-0.47) | 0.36 (0.23-0.57) | -0.02 (-0.41-0.38) |
| Thailand | 154.78 (89.61-250.53) | 181.52 (98.49-259.29) | 0.17 | 0.6 (0.35-0.97) | 0.86 (0.46-1.22) | 0.97 (0.64-1.29) |
| Timor-Leste | 1.92 (0.8-4.48) | 2.42 (1.05-4.46) | 0.26 | 0.6 (0.25-1.41) | 0.42 (0.18-0.78) | -1.53 (-1.98--1.08) |
| Togo | 11.9 (6.72-19.93) | 34.83 (21.48-55.12) | 1.93 | 0.87 (0.49-1.45) | 1.04 (0.64-1.64) | 0.58 (0.45-0.7) |
| Tokelau | 0 (0-0.01) | 0 (0-0) | NA | 0.68 (0.31-1.27) | 0.65 (0.43-0.95) | -0.79 (-1.04--0.53) |
| Tonga | 0.23 (0.1-0.4) | 0.28 (0.16-0.45) | 0.22 | 0.63 (0.28-1.08) | 0.71 (0.42-1.15) | 0.51 (0.28-0.74) |
| Trinidad and Tobago | 2.19 (1.94-2.44) | 2.47 (1.86-3.18) | 0.13 | 0.44 (0.39-0.49) | 0.5 (0.37-0.64) | 0.2 (-0.11-0.51) |
| Tunisia | 2.09 (1.13-4) | 3.34 (2.11-5.37) | 0.6 | 0.06 (0.03-0.12) | 0.08 (0.05-0.12) | 0.94 (0.85-1.03) |
| T眉rkiye | 47.43 (31.27-70.36) | 33.16 (24.18-44.58) | -0.3 | 0.2 (0.13-0.29) | 0.1 (0.08-0.14) | -2.04 (-2.1--1.98) |
| Turkmenistan | 7.72 (6.27-9.44) | 15.28 (10.52-21.97) | 0.98 | 0.5 (0.41-0.62) | 0.73 (0.51-1.06) | 0.78 (0.56-1) |
| Tuvalu | 0.03 (0.02-0.05) | 0.03 (0.02-0.05) | 0 | 0.79 (0.45-1.27) | 0.66 (0.42-1.02) | -0.75 (-0.88--0.61) |
| Uganda | 12.39 (6.28-23.55) | 41.63 (23.5-71.4) | 2.36 | 0.19 (0.1-0.37) | 0.24 (0.14-0.42) | 0.33 (0.16-0.5) |
| Ukraine | 446.55 (400.12-495.93) | 520.53 (373.39-694.87) | 0.17 | 2.35 (2.11-2.61) | 3.78 (2.71-5.04) | 0.57 (0.21-0.93) |
| United Arab Emirates | 1.84 (1.16-3.13) | 5.74 (3.61-8.52) | 2.12 | 0.19 (0.12-0.33) | 0.14 (0.09-0.21) | -0.72 (-0.98--0.45) |
| United Kingdom | 56.47 (55.57-57.45) | 59.75 (54.72-64.77) | 0.06 | 0.27 (0.27-0.27) | 0.27 (0.25-0.3) | 0.08 (-0.33-0.5) |
| United Republic of Tanzania | 27.42 (13.65-45.07) | 86.96 (40.83-146.66) | 2.17 | 0.28 (0.14-0.47) | 0.37 (0.17-0.63) | 1.21 (0.96-1.45) |
| United States of America | 331.26 (321.56-343.9) | 379.24 (359.8-395.89) | 0.14 | 0.32 (0.31-0.34) | 0.34 (0.32-0.36) | 0.05 (-0.19-0.3) |
| United States Virgin Islands | 0.23 (0.16-0.31) | 0.21 (0.12-0.34) | -0.09 | 0.57 (0.4-0.78) | 0.9 (0.51-1.46) | 2.28 (1.89-2.66) |
| Uruguay | 5.69 (5.14-6.29) | 5.07 (4.55-5.6) | -0.11 | 0.5 (0.45-0.55) | 0.42 (0.38-0.47) | -0.53 (-0.86--0.21) |
| Uzbekistan | 34.74 (28.02-42.76) | 77.61 (61.07-97.05) | 1.23 | 0.4 (0.33-0.5) | 0.57 (0.44-0.71) | 0.39 (-0.21-1) |
| Vanuatu | 0.46 (0.2-0.9) | 0.93 (0.5-1.56) | 1.02 | 0.78 (0.35-1.54) | 0.74 (0.4-1.25) | -0.36 (-0.52--0.21) |
| Venezuela (Bolivarian Republic of) | 35.1 (31.49-39.24) | 50.34 (36.98-65.03) | 0.43 | 0.44 (0.39-0.49) | 0.54 (0.4-0.69) | 0.84 (0.58-1.1) |
| Viet Nam | 113.89 (61.61-193.03) | 149.6 (71.24-257.81) | 0.31 | 0.4 (0.22-0.68) | 0.39 (0.19-0.67) | -0.47 (-0.7--0.24) |
| Yemen | 1.86 (0.94-3.28) | 7.61 (4.7-12.66) | 3.09 | 0.04 (0.02-0.07) | 0.06 (0.03-0.09) | 1.31 (0.94-1.68) |
| Zambia | 9.37 (5.94-14.37) | 32.53 (18.03-55.33) | 2.47 | 0.31 (0.2-0.47) | 0.4 (0.22-0.68) | 0.86 (0.69-1.04) |
| Zimbabwe | 13.33 (6.19-21.44) | 34.27 (17.46-58.17) | 1.57 | 0.34 (0.16-0.54) | 0.54 (0.28-0.92) | 1.7 (1.26-2.14) |
