# Supplementary material for: Global burden of pancreatitis among individuals aged 15–39 years: a systematic analysis from the 2021 GBD study
Source: Front Med (Lausanne). 2025 May 27;12:1572346. doi: 10.3389/fmed.2025.1572346 (PMC12150401; doi:10.3389/fmed.2025.1572346)
Supplement: Supplementary file 8 [file Supplementary_file_8.docx]

**Supplementary Table 8** The incidence of pancreatitis burden in people aged 15-39 years in global and 5 cases and rates, and the trends in age patterns from 1990 to 2021

| **location** | **Age (year)** | **Incidence cases** | | | **Incidence rates** | | |
| --- | --- | --- | --- | --- | --- | --- | --- |
|  |  | **1990 thousand**  **(95%UI)** | **2021 thousand**  **(95%UI)** | **percentage**  **Change**  **(100%)** | **1990**  **per (95%UI)** | **2021**  **per (95%UI)** | **EAPC**  **(95% CI)** |
| Global | 15-19 years | 63.05 (38.8-92.94) | 83.43 (52.72-121.44) | 0.32 (0.36-0.31) | 12.14 (7.47-17.89) | 13.37 (8.45-19.46) | 0.33 (0.31-0.36) |
| Global | 15-39 years | 607.83 (438.15-796.81) | 814.5 (606.27-1056.9) | 0.34 (0.38-0.33) | 27.73 (19.99-36.35) | 27.38 (20.38-35.53) | -0.09 (-0.16--0.03) |
| Global | 20-24 years | 86.48 (55.07-131.36) | 110.72 (72.51-162.79) | 0.28 (0.32-0.24) | 17.57 (11.19-26.7) | 18.54 (12.14-27.26) | 0.13 (0.1-0.16) |
| Global | 25-29 years | 121.11 (78.67-177.33) | 153.04 (100.84-224.31) | 0.26 (0.28-0.26) | 27.36 (17.77-40.06) | 26.01 (17.14-38.13) | -0.13 (-0.18--0.08) |
| Global | 30-34 years | 156.3 (90.27-234.52) | 217.4 (130.31-319.74) | 0.39 (0.44-0.36) | 40.55 (23.42-60.85) | 35.97 (21.56-52.9) | -0.34 (-0.45--0.23) |
| Global | 35-39 years | 180.9 (115.29-266.61) | 249.92 (161.58-363.77) | 0.38 (0.4-0.36) | 51.36 (32.73-75.69) | 44.56 (28.81-64.86) | -0.53 (-0.66--0.41) |
| Global | 15-19 years | 5.43 (3.22-8.17) | 14.46 (8.69-21.79) | 1.66 (1.7-1.67) | 10.73 (6.35-16.13) | 11.67 (7.01-17.58) | 0.34 (0.26-0.43) |
| Global | 15-39 years | 36.15 (25.25-48.08) | 91.54 (64.55-121.73) | 1.53 (1.56-1.53) | 19.61 (13.7-26.09) | 20.39 (14.38-27.11) | 0.19 (0.16-0.22) |
| Low SDI | 20-24 years | 6.23 (3.88-9.65) | 16.23 (10.14-24.84) | 1.61 (1.61-1.57) | 14.62 (9.1-22.65) | 15.56 (9.72-23.82) | 0.25 (0.2-0.3) |
| Low SDI | 25-29 years | 7.33 (4.56-11.31) | 18.28 (11.4-27.89) | 1.49 (1.5-1.47) | 20.47 (12.72-31.58) | 21.23 (13.24-32.38) | 0.14 (0.12-0.16) |
| Low SDI | 30-34 years | 8.37 (4.48-13.27) | 20.95 (11.39-33.03) | 1.5 (1.54-1.49) | 28.18 (15.08-44.69) | 28.94 (15.74-45.62) | 0.12 (0.09-0.14) |
| Low SDI | 35-39 years | 8.78 (5.2-13.44) | 21.62 (13.12-33.22) | 1.46 (1.52-1.47) | 34.42 (20.4-52.68) | 34.72 (21.06-53.35) | 0.07 (0.06-0.09) |
| Low SDI | 15-19 years | 15.65 (9.54-23.43) | 29.6 (18.03-44.15) | 0.89 (0.89-0.88) | 13.16 (8.02-19.7) | 16.04 (9.77-23.92) | 0.8 (0.65-0.96) |
| Low SDI | 15-39 years | 113.78 (79.98-150.99) | 230.94 (161.97-304.2) | 1.03 (1.03-1.01) | 25.09 (17.64-33.3) | 28.78 (20.18-37.91) | 0.52 (0.47-0.57) |
| Low SDI | 20-24 years | 18.86 (11.71-29.02) | 37.12 (23.24-57.01) | 0.97 (0.98-0.96) | 18.09 (11.23-27.83) | 21.23 (13.3-32.61) | 0.65 (0.55-0.74) |
| Low SDI | 25-29 years | 23.17 (14.57-35.81) | 46.31 (29.28-71.79) | 1 (1.01-1) | 25.85 (16.26-39.96) | 28.61 (18.09-44.35) | 0.42 (0.37-0.47) |
| Low-middle SDI | 30-34 years | 27.36 (14.96-43.45) | 57.22 (31.24-90.93) | 1.09 (1.09-1.09) | 36.23 (19.81-57.55) | 38.7 (21.14-61.51) | 0.28 (0.24-0.32) |
| Low-middle SDI | 35-39 years | 28.74 (17.27-43.79) | 60.69 (36.84-92.82) | 1.11 (1.13-1.12) | 44.16 (26.53-67.3) | 45.48 (27.61-69.56) | 0.15 (0.12-0.19) |
| Low-middle SDI | 15-19 years | 20.5 (12.18-30.56) | 22.23 (13.8-32.74) | 0.08 (0.13-0.07) | 10.94 (6.5-16.31) | 12.19 (7.57-17.96) | 0.44 (0.35-0.52) |
| Low-middle SDI | 15-39 years | 176.17 (125.46-234.13) | 236.02 (171.53-310.32) | 0.34 (0.37-0.33) | 23.41 (16.67-31.11) | 25.45 (18.49-33.46) | 0.21 (0.11-0.3) |
| Low-middle SDI | 20-24 years | 27.65 (17.13-42.65) | 30.48 (19.64-45.27) | 0.1 (0.15-0.06) | 15.51 (9.61-23.92) | 17.2 (11.08-25.54) | 0.31 (0.22-0.39) |
| Low-middle SDI | 25-29 years | 35.71 (22.37-54.27) | 44.7 (28.84-66.82) | 0.25 (0.29-0.23) | 23.65 (14.82-35.95) | 24.32 (15.69-36.35) | 0.04 (-0.06-0.14) |
| Low-middle SDI | 30-34 years | 42.42 (23.22-67.04) | 65.26 (37.71-98.25) | 0.54 (0.62-0.47) | 34.61 (18.95-54.7) | 32.71 (18.9-49.25) | -0.16 (-0.28--0.04) |
| Low-middle SDI | 35-39 years | 49.89 (30.38-75.65) | 73.35 (45.82-109.14) | 0.47 (0.51-0.44) | 43.97 (26.77-66.68) | 39.73 (24.82-59.12) | -0.31 (-0.46--0.16) |
| Middle SDI | 15-19 years | 12.69 (7.97-18.46) | 8.9 (5.71-12.43) | -0.3 (-0.28--0.33) | 13.14 (8.26-19.12) | 12.28 (7.88-17.15) | -0.47 (-0.55--0.39) |
| Middle SDI | 15-39 years | 161.24 (117.85-211.31) | 146.87 (109.23-192.98) | -0.09 (-0.07--0.09) | 35.63 (26.04-46.69) | 33.36 (24.81-43.83) | -0.28 (-0.38--0.18) |
| Middle SDI | 20-24 years | 18.93 (12.48-27.46) | 13.17 (9.04-18.43) | -0.3 (-0.28--0.33) | 19.4 (12.79-28.14) | 17.56 (12.06-24.58) | -0.49 (-0.61--0.38) |
| Middle SDI | 25-29 years | 30.78 (20.36-44.39) | 23.13 (15.81-32.19) | -0.25 (-0.22--0.27) | 33.12 (21.91-47.77) | 27.32 (18.67-38.01) | -0.41 (-0.55--0.27) |
| Middle SDI | 30-34 years | 44.81 (28.13-66.23) | 44.59 (28.74-63.37) | 0 (0.02--0.04) | 52.56 (33-77.68) | 41.79 (26.94-59.39) | -0.53 (-0.72--0.34) |
| Middle SDI | 35-39 years | 54.02 (35.1-78.99) | 57.08 (38.64-79.99) | 0.06 (0.1-0.01) | 67.34 (43.74-98.45) | 56.27 (38.09-78.86) | -0.72 (-0.92--0.53) |
| Middle SDI | 15-19 years | 8.72 (5.36-12.71) | 8.19 (5.96-10.65) | -0.06 (0.11--0.16) | 13.31 (8.18-19.41) | 13.61 (9.9-17.7) | -0.19 (-0.27--0.11) |
| Middle SDI | 15-39 years | 119.94 (89.45-154.65) | 108.59 (89.31-129.22) | -0.09 (0--0.16) | 34.57 (25.78-44.57) | 30.74 (25.28-36.58) | -0.66 (-0.8--0.52) |
| High-middle SDI | 20-24 years | 14.74 (9.77-21.77) | 13.66 (10.29-17.47) | -0.07 (0.05--0.2) | 21.39 (14.18-31.61) | 20.88 (15.74-26.71) | -0.36 (-0.46--0.26) |
| High-middle SDI | 25-29 years | 24.02 (16.17-34.43) | 20.52 (15.33-26.17) | -0.15 (-0.05--0.24) | 32.95 (22.18-47.24) | 28.74 (21.48-36.66) | -0.66 (-0.78--0.54) |
| High-middle SDI | 30-34 years | 33.18 (20.62-47.74) | 29.24 (21.29-38.42) | -0.12 (0.03--0.2) | 46.06 (28.62-66.28) | 37.68 (27.43-49.51) | -0.84 (-0.98--0.71) |
| High-middle SDI | 35-39 years | 39.28 (25.72-56.72) | 36.99 (27.01-49.01) | -0.06 (0.05--0.14) | 58.07 (38.03-83.85) | 47.02 (34.33-62.31) | -0.89 (-1.02--0.76) |
| High-middle SDI | 15-19 years | 63.05 (38.8-92.94) | 83.43 (52.72-121.44) | 0.32 (0.36-0.31) | 12.14 (7.47-17.89) | 13.37 (8.45-19.46) | 0.33 (0.31-0.36) |
| High-middle SDI | 15-39 years | 607.83 (438.15-796.81) | 814.5 (606.27-1056.9) | 0.34 (0.38-0.33) | 27.73 (19.99-36.35) | 27.38 (20.38-35.53) | -0.09 (-0.16--0.03) |
| High-middle SDI | 20-24 years | 86.48 (55.07-131.36) | 110.72 (72.51-162.79) | 0.28 (0.32-0.24) | 17.57 (11.19-26.7) | 18.54 (12.14-27.26) | 0.13 (0.1-0.16) |
| High-middle SDI | 25-29 years | 121.11 (78.67-177.33) | 153.04 (100.84-224.31) | 0.26 (0.28-0.26) | 27.36 (17.77-40.06) | 26.01 (17.14-38.13) | -0.13 (-0.18--0.08) |
| High SDI | 30-34 years | 156.3 (90.27-234.52) | 217.4 (130.31-319.74) | 0.39 (0.44-0.36) | 40.55 (23.42-60.85) | 35.97 (21.56-52.9) | -0.34 (-0.45--0.23) |
| High SDI | 35-39 years | 180.9 (115.29-266.61) | 249.92 (161.58-363.77) | 0.38 (0.4-0.36) | 51.36 (32.73-75.69) | 44.56 (28.81-64.86) | -0.53 (-0.66--0.41) |
| High SDI | 15-19 years | 5.43 (3.22-8.17) | 14.46 (8.69-21.79) | 1.66 (1.7-1.67) | 10.73 (6.35-16.13) | 11.67 (7.01-17.58) | 0.34 (0.26-0.43) |
| High SDI | 15-39 years | 36.15 (25.25-48.08) | 91.54 (64.55-121.73) | 1.53 (1.56-1.53) | 19.61 (13.7-26.09) | 20.39 (14.38-27.11) | 0.19 (0.16-0.22) |
| High SDI | 20-24 years | 6.23 (3.88-9.65) | 16.23 (10.14-24.84) | 1.61 (1.61-1.57) | 14.62 (9.1-22.65) | 15.56 (9.72-23.82) | 0.25 (0.2-0.3) |
| High SDI | 25-29 years | 7.33 (4.56-11.31) | 18.28 (11.4-27.89) | 1.49 (1.5-1.47) | 20.47 (12.72-31.58) | 21.23 (13.24-32.38) | 0.14 (0.12-0.16) |
| High SDI | 30-34 years | 8.37 (4.48-13.27) | 20.95 (11.39-33.03) | 1.5 (1.54-1.49) | 28.18 (15.08-44.69) | 28.94 (15.74-45.62) | 0.12 (0.09-0.14) |
| High SDI | 35-39 years | 8.78 (5.2-13.44) | 21.62 (13.12-33.22) | 1.46 (1.52-1.47) | 34.42 (20.4-52.68) | 34.72 (21.06-53.35) | 0.07 (0.06-0.09) |
