# Supplementary material for: Global burden of pancreatitis among individuals aged 15–39 years: a systematic analysis from the 2021 GBD study
Source: Front Med (Lausanne). 2025 May 27;12:1572346. doi: 10.3389/fmed.2025.1572346 (PMC12150401; doi:10.3389/fmed.2025.1572346)
Supplement: Supplementary file 9 [file Supplementary_file_9.docx]

**Supplementary Table 9** The DALY of pancreatitis burden in people aged 15-39 years in global and 5 cases and rates, and the trends in age patterns from 1990 to 2021

| **location** | **Age (year)** | **DALYs cases** | | | **DALYs rates** | | |
| --- | --- | --- | --- | --- | --- | --- | --- |
|  |  | **1990 thousand**  **(95%UI)** | **2021 thousand**  **(95%UI)** | **percentage**  **Change**  **(100%)** | **1990**  **per (95%UI)** | **2021**  **per (95%UI)** | **EAPC**  **(95% CI)** |
| Global | 15-19 years | 70.6 (58.44-95.63) | 69.72 (56.76-86.23) | -0.01 (-0.03--0.1) | 13.59 (11.25-18.41) | 11.17 (9.1-13.82) | -0.66 (-0.82--0.51) |
| Global | 15-39 years | 836.49 (728.56-1002.3) | 1056.43 (936.94-1238.99) | 0.26 (0.29-0.24) | 38.16 (33.24-45.73) | 35.51 (31.5-41.65) | -0.33 (-0.47--0.2) |
| Global | 20-24 years | 110.86 (91.59-137.31) | 114.81 (96.63-142.1) | 0.04 (0.06-0.03) | 22.53 (18.61-27.9) | 19.23 (16.18-23.8) | -0.67 (-0.89--0.46) |
| Global | 25-29 years | 169.49 (145.82-213.77) | 203.56 (174.31-245.44) | 0.2 (0.2-0.15) | 38.29 (32.95-48.3) | 34.6 (29.63-41.72) | -0.33 (-0.59--0.07) |
| Global | 30-34 years | 219.24 (190.81-268.94) | 290.76 (255.03-346.96) | 0.33 (0.34-0.29) | 56.88 (49.51-69.78) | 48.1 (42.19-57.4) | -0.48 (-0.67--0.3) |
| Global | 35-39 years | 266.28 (232.33-324.35) | 377.59 (333.84-450.43) | 0.42 (0.44-0.39) | 75.6 (65.96-92.08) | 67.32 (59.52-80.31) | -0.49 (-0.6--0.39) |
| Global | 15-19 years | 8.42 (6.12-12.99) | 14.68 (11.48-18.96) | 0.74 (0.88-0.46) | 16.62 (12.09-25.64) | 11.84 (9.26-15.29) | -1.11 (-1.36--0.86) |
| Global | 15-39 years | 76.49 (54.25-105.76) | 149.57 (112.42-200.76) | 0.96 (1.07-0.9) | 41.5 (29.43-57.38) | 33.31 (25.04-44.71) | -0.79 (-0.87--0.71) |
| Low SDI | 20-24 years | 11.14 (8.16-16.06) | 20.02 (15.47-27.84) | 0.8 (0.9-0.73) | 26.14 (19.14-37.67) | 19.19 (14.84-26.7) | -1 (-1.17--0.84) |
| Low SDI | 25-29 years | 16.88 (11.51-23.85) | 33.15 (24.31-44.86) | 0.96 (1.11-0.88) | 47.13 (32.13-66.58) | 38.48 (28.23-52.08) | -0.88 (-1.01--0.76) |
| Low SDI | 30-34 years | 17.26 (11.44-25.4) | 33.38 (23.94-46.31) | 0.93 (1.09-0.82) | 58.1 (38.52-85.51) | 46.1 (33.07-63.96) | -1.06 (-1.17--0.94) |
| Low SDI | 35-39 years | 22.79 (15.14-33.77) | 48.35 (32.91-68.11) | 1.12 (1.17-1.02) | 89.33 (59.33-132.38) | 77.65 (52.86-109.38) | -0.42 (-0.51--0.34) |
| Low SDI | 15-19 years | 27.05 (20.66-40.99) | 29.69 (22.2-39.2) | 0.1 (0.07--0.04) | 22.74 (17.37-34.47) | 16.09 (12.03-21.24) | -1.12 (-1.38--0.86) |
| Low SDI | 15-39 years | 238.44 (188.1-330.62) | 330.45 (269.23-412.68) | 0.39 (0.43-0.25) | 52.59 (41.49-72.92) | 41.18 (33.55-51.42) | -0.81 (-0.92--0.69) |
| Low SDI | 20-24 years | 39.06 (29.05-54.06) | 45.79 (34.68-60.04) | 0.17 (0.19-0.11) | 37.45 (27.86-51.84) | 26.2 (19.84-34.35) | -1.29 (-1.49--1.1) |
| Low SDI | 25-29 years | 52.71 (41.24-75.43) | 71.14 (56.7-91.94) | 0.35 (0.37-0.22) | 58.81 (46.02-84.16) | 43.95 (35.03-56.8) | -0.99 (-1.12--0.85) |
| Low-middle SDI | 30-34 years | 57.07 (43.48-83.21) | 83.39 (67.33-108.41) | 0.46 (0.55-0.3) | 75.59 (57.58-110.21) | 56.41 (45.54-73.33) | -0.97 (-1.07--0.86) |
| Low-middle SDI | 35-39 years | 62.55 (48.18-88.32) | 100.44 (81.51-128.85) | 0.61 (0.69-0.46) | 96.13 (74.04-135.72) | 75.27 (61.09-96.56) | -0.64 (-0.76--0.53) |
| Low-middle SDI | 15-19 years | 21.22 (17.8-25.76) | 17.04 (14.52-20.01) | -0.2 (-0.18--0.22) | 11.33 (9.5-13.75) | 9.35 (7.96-10.98) | -0.39 (-0.52--0.26) |
| Low-middle SDI | 15-39 years | 232.93 (206.14-276.4) | 281.06 (244.72-327.01) | 0.21 (0.19-0.18) | 30.95 (27.39-36.72) | 30.3 (26.39-35.26) | -0.08 (-0.12--0.04) |
| Low-middle SDI | 20-24 years | 34.17 (27.94-41.28) | 31.11 (26.5-36.76) | -0.09 (-0.05--0.11) | 19.17 (15.67-23.15) | 17.55 (14.95-20.74) | -0.24 (-0.3--0.18) |
| Low-middle SDI | 25-29 years | 49.73 (42.87-61.79) | 56.76 (48.26-66.81) | 0.14 (0.13-0.08) | 32.94 (28.4-40.93) | 30.88 (26.26-36.35) | -0.25 (-0.36--0.13) |
| Low-middle SDI | 30-34 years | 59.34 (51.09-72.18) | 80.16 (66.56-94.76) | 0.35 (0.3-0.31) | 48.41 (41.69-58.89) | 40.18 (33.37-47.5) | -0.46 (-0.58--0.34) |
| Low-middle SDI | 35-39 years | 68.47 (57.9-84.46) | 96 (82.6-115.91) | 0.4 (0.43-0.37) | 60.35 (51.04-74.44) | 52 (44.74-62.78) | -0.42 (-0.51--0.33) |
| Middle SDI | 15-19 years | 10.05 (8.47-12.38) | 5.89 (5.07-7.23) | -0.41 (-0.4--0.42) | 10.41 (8.77-12.82) | 8.13 (7-9.98) | -1.25 (-1.49--1.02) |
| Middle SDI | 15-39 years | 198.11 (180.38-222.27) | 224.56 (199.13-253.59) | 0.13 (0.1-0.14) | 43.78 (39.86-49.12) | 51.01 (45.23-57.6) | 0.26 (-0.11-0.63) |
| Middle SDI | 20-24 years | 19.01 (16.7-22.32) | 12.27 (10.5-14.4) | -0.35 (-0.37--0.35) | 19.48 (17.11-22.87) | 16.36 (14-19.21) | -1.01 (-1.69--0.32) |
| Middle SDI | 25-29 years | 35 (31.49-40.57) | 30.55 (26.13-35.55) | -0.13 (-0.17--0.12) | 37.66 (33.88-43.66) | 36.08 (30.85-41.97) | 0.14 (-0.68-0.96) |
| Middle SDI | 30-34 years | 58.6 (53.32-66.12) | 72.53 (62.71-83.44) | 0.24 (0.18-0.26) | 68.73 (62.54-77.55) | 67.98 (58.77-78.2) | 0.18 (-0.35-0.7) |
| Middle SDI | 35-39 years | 75.45 (68.41-84.83) | 103.31 (92.63-116.93) | 0.37 (0.35-0.38) | 94.04 (85.27-105.74) | 101.85 (91.32-115.28) | -0.2 (-0.47-0.08) |
| Middle SDI | 15-19 years | 3.82 (3.31-4.62) | 2.39 (2.07-2.9) | -0.37 (-0.37--0.37) | 5.84 (5.06-7.05) | 3.97 (3.44-4.82) | -1.56 (-1.68--1.43) |
| Middle SDI | 15-39 years | 89.51 (82.56-99.43) | 70.01 (64.71-77.06) | -0.22 (-0.22--0.22) | 25.8 (23.79-28.66) | 19.82 (18.32-21.81) | -1.07 (-1.2--0.95) |
| High-middle SDI | 20-24 years | 7.39 (6.62-8.57) | 5.54 (4.97-6.36) | -0.25 (-0.25--0.26) | 10.73 (9.61-12.45) | 8.47 (7.6-9.72) | -0.86 (-0.97--0.75) |
| High-middle SDI | 25-29 years | 15 (13.69-17.1) | 11.82 (10.88-13.21) | -0.21 (-0.21--0.23) | 20.58 (18.78-23.47) | 16.56 (15.24-18.5) | -0.64 (-0.74--0.53) |
| High-middle SDI | 30-34 years | 26.68 (24.35-29.73) | 21.08 (19.43-23.37) | -0.21 (-0.2--0.21) | 37.05 (33.81-41.28) | 27.17 (25.04-30.11) | -1.05 (-1.17--0.93) |
| High-middle SDI | 35-39 years | 36.61 (33.92-40.32) | 29.18 (26.89-32.01) | -0.2 (-0.21--0.21) | 54.12 (50.15-59.6) | 37.09 (34.19-40.7) | -1.46 (-1.58--1.34) |
| High-middle SDI | 15-19 years | 70.6 (58.44-95.63) | 69.72 (56.76-86.23) | -0.01 (-0.03--0.1) | 13.59 (11.25-18.41) | 11.17 (9.1-13.82) | -0.66 (-0.82--0.51) |
| High-middle SDI | 15-39 years | 836.49 (728.56-1002.3) | 1056.43 (936.94-1238.99) | 0.26 (0.29-0.24) | 38.16 (33.24-45.73) | 35.51 (31.5-41.65) | -0.33 (-0.47--0.2) |
| High-middle SDI | 20-24 years | 110.86 (91.59-137.31) | 114.81 (96.63-142.1) | 0.04 (0.06-0.03) | 22.53 (18.61-27.9) | 19.23 (16.18-23.8) | -0.67 (-0.89--0.46) |
| High-middle SDI | 25-29 years | 169.49 (145.82-213.77) | 203.56 (174.31-245.44) | 0.2 (0.2-0.15) | 38.29 (32.95-48.3) | 34.6 (29.63-41.72) | -0.33 (-0.59--0.07) |
| High SDI | 30-34 years | 219.24 (190.81-268.94) | 290.76 (255.03-346.96) | 0.33 (0.34-0.29) | 56.88 (49.51-69.78) | 48.1 (42.19-57.4) | -0.48 (-0.67--0.3) |
| High SDI | 35-39 years | 266.28 (232.33-324.35) | 377.59 (333.84-450.43) | 0.42 (0.44-0.39) | 75.6 (65.96-92.08) | 67.32 (59.52-80.31) | -0.49 (-0.6--0.39) |
| High SDI | 15-19 years | 8.42 (6.12-12.99) | 14.68 (11.48-18.96) | 0.74 (0.88-0.46) | 16.62 (12.09-25.64) | 11.84 (9.26-15.29) | -1.11 (-1.36--0.86) |
| High SDI | 15-39 years | 76.49 (54.25-105.76) | 149.57 (112.42-200.76) | 0.96 (1.07-0.9) | 41.5 (29.43-57.38) | 33.31 (25.04-44.71) | -0.79 (-0.87--0.71) |
| High SDI | 20-24 years | 11.14 (8.16-16.06) | 20.02 (15.47-27.84) | 0.8 (0.9-0.73) | 26.14 (19.14-37.67) | 19.19 (14.84-26.7) | -1 (-1.17--0.84) |
| High SDI | 25-29 years | 16.88 (11.51-23.85) | 33.15 (24.31-44.86) | 0.96 (1.11-0.88) | 47.13 (32.13-66.58) | 38.48 (28.23-52.08) | -0.88 (-1.01--0.76) |
| High SDI | 30-34 years | 17.26 (11.44-25.4) | 33.38 (23.94-46.31) | 0.93 (1.09-0.82) | 58.1 (38.52-85.51) | 46.1 (33.07-63.96) | -1.06 (-1.17--0.94) |
| High SDI | 35-39 years | 22.79 (15.14-33.77) | 48.35 (32.91-68.11) | 1.12 (1.17-1.02) | 89.33 (59.33-132.38) | 77.65 (52.86-109.38) | -0.42 (-0.51--0.34) |
