# Supplementary material for: Global burden of pancreatitis among individuals aged 15–39 years: a systematic analysis from the 2021 GBD study
Source: Front Med (Lausanne). 2025 May 27;12:1572346. doi: 10.3389/fmed.2025.1572346 (PMC12150401; doi:10.3389/fmed.2025.1572346)
Supplement: Supplementary file 10 [file Supplementary_file_10.docx]

**Supplementary Table 10** The deaths of pancreatitis burden in people aged 15-39 years in global and 5 cases and rates, and the trends in age patterns from 1990 to 2021

| **location** | **Age (year)** | **Deaths cases** | | | **Deaths rates** | | |
| --- | --- | --- | --- | --- | --- | --- | --- |
|  |  | **1990 thousand**  **(95%UI)** | **2021 thousand**  **(95%UI)** | **percentage**  **Change**  **(100%)** | **1990**  **per (95%UI)** | **2021**  **per (95%UI)** | **EAPC**  **(95% CI)** |
| Global | 15-19 years | 885.15 (716.32-1209.36) | 861.85 (701.17-1096.75) | -0.03 (-0.02--0.09) | 0.17 (0.14-0.23) | 0.14 (0.11-0.18) | -0.7 (-0.87--0.53) |
| Global | 15-39 years | 13069.17 (11366.38-15762.89) | 16799.91 (14826.03-19749.17) | 0.29 (0.3-0.25) | 0.6 (0.52-0.72) | 0.56 (0.5-0.66) | -0.29 (-0.44--0.15) |
| Global | 20-24 years | 1511.61 (1231.95-1927.34) | 1554.35 (1288.06-1965.28) | 0.03 (0.05-0.02) | 0.31 (0.25-0.39) | 0.26 (0.22-0.33) | -0.71 (-0.93--0.48) |
| Global | 25-29 years | 2509.23 (2142.8-3204.68) | 3032.72 (2568.34-3712.93) | 0.21 (0.2-0.16) | 0.57 (0.48-0.72) | 0.52 (0.44-0.63) | -0.33 (-0.6--0.06) |
| Global | 30-34 years | 3512.45 (3058.86-4408.76) | 4690.77 (4072.68-5640.71) | 0.34 (0.33-0.28) | 0.91 (0.79-1.14) | 0.78 (0.67-0.93) | -0.47 (-0.67--0.28) |
| Global | 35-39 years | 4650.73 (4058.92-5751.66) | 6660.22 (5823.63-7968.7) | 0.43 (0.43-0.39) | 1.32 (1.15-1.63) | 1.19 (1.04-1.42) | -0.46 (-0.57--0.35) |
| Global | 15-19 years | 109.22 (78.12-171.91) | 185.25 (142.11-244.03) | 0.7 (0.82-0.42) | 0.22 (0.15-0.34) | 0.15 (0.11-0.2) | -1.2 (-1.46--0.94) |
| Global | 15-39 years | 1229.87 (848.44-1726.6) | 2394.37 (1766.5-3228.14) | 0.95 (1.08-0.87) | 0.67 (0.46-0.94) | 0.53 (0.39-0.72) | -0.8 (-0.88--0.72) |
| Low SDI | 20-24 years | 156.23 (111.84-227.35) | 274.66 (210.51-395.57) | 0.76 (0.88-0.74) | 0.37 (0.26-0.53) | 0.26 (0.2-0.38) | -1.07 (-1.25--0.9) |
| Low SDI | 25-29 years | 259 (173.57-371.44) | 503.5 (363.29-687.15) | 0.94 (1.09-0.85) | 0.72 (0.48-1.04) | 0.58 (0.42-0.8) | -0.92 (-1.05--0.79) |
| Low SDI | 30-34 years | 287.01 (186.41-427.31) | 548.68 (388.11-775.6) | 0.91 (1.08-0.82) | 0.97 (0.63-1.44) | 0.76 (0.54-1.07) | -1.11 (-1.23--0.99) |
| Low SDI | 35-39 years | 418.42 (272.5-626.52) | 882.27 (595.38-1262.03) | 1.11 (1.18-1.01) | 1.64 (1.07-2.46) | 1.42 (0.96-2.03) | -0.44 (-0.53--0.35) |
| Low SDI | 15-19 years | 354.62 (269.61-542.9) | 379.34 (277.82-511.45) | 0.07 (0.03--0.06) | 0.3 (0.23-0.46) | 0.21 (0.15-0.28) | -1.21 (-1.48--0.93) |
| Low SDI | 15-39 years | 3816.18 (2975.04-5335.11) | 5283.06 (4281.03-6597.72) | 0.38 (0.44-0.24) | 0.84 (0.66-1.18) | 0.66 (0.53-0.82) | -0.81 (-0.92--0.69) |
| Low SDI | 20-24 years | 553.62 (406.29-776.03) | 635.17 (476.49-837.15) | 0.15 (0.17-0.08) | 0.53 (0.39-0.74) | 0.36 (0.27-0.48) | -1.37 (-1.58--1.17) |
| Low SDI | 25-29 years | 810.79 (628.36-1178.14) | 1079.18 (855.07-1406.02) | 0.33 (0.36-0.19) | 0.9 (0.7-1.31) | 0.67 (0.53-0.87) | -1.03 (-1.17--0.89) |
| Low-middle SDI | 30-34 years | 952.63 (717.71-1412.57) | 1372.38 (1092.77-1827.23) | 0.44 (0.52-0.29) | 1.26 (0.95-1.87) | 0.93 (0.74-1.24) | -1.01 (-1.12--0.9) |
| Low-middle SDI | 35-39 years | 1144.51 (872.93-1637.27) | 1816.98 (1434.01-2358.68) | 0.59 (0.64-0.44) | 1.76 (1.34-2.52) | 1.36 (1.07-1.77) | -0.67 (-0.8--0.55) |
| Low-middle SDI | 15-19 years | 267.8 (220.99-327.15) | 209.58 (176.99-247.24) | -0.22 (-0.2--0.24) | 0.14 (0.12-0.17) | 0.11 (0.1-0.14) | -0.45 (-0.59--0.31) |
| Low-middle SDI | 15-39 years | 3681.65 (3222.81-4392.21) | 4482.1 (3875.57-5245.12) | 0.22 (0.2-0.19) | 0.49 (0.43-0.58) | 0.48 (0.42-0.57) | -0.06 (-0.1--0.02) |
| Low-middle SDI | 20-24 years | 470.04 (383.9-568.98) | 422.33 (357.21-504.09) | -0.1 (-0.07--0.11) | 0.26 (0.22-0.32) | 0.24 (0.2-0.28) | -0.28 (-0.34--0.22) |
| Low-middle SDI | 25-29 years | 746.71 (634.31-935.24) | 847.57 (715.77-1008.94) | 0.14 (0.13-0.08) | 0.49 (0.42-0.62) | 0.46 (0.39-0.55) | -0.27 (-0.39--0.15) |
| Low-middle SDI | 30-34 years | 972.25 (829.48-1196.55) | 1300.01 (1065.64-1543.67) | 0.34 (0.28-0.29) | 0.79 (0.68-0.98) | 0.65 (0.53-0.77) | -0.49 (-0.62--0.37) |
| Low-middle SDI | 35-39 years | 1224.85 (1026.44-1539.36) | 1702.6 (1435.05-2090.34) | 0.39 (0.4-0.36) | 1.08 (0.9-1.36) | 0.92 (0.78-1.13) | -0.44 (-0.53--0.35) |
| Middle SDI | 15-19 years | 112.48 (97.12-133) | 63.47 (58.53-71.72) | -0.44 (-0.4--0.46) | 0.12 (0.1-0.14) | 0.09 (0.08-0.1) | -1.36 (-1.61--1.11) |
| Middle SDI | 15-39 years | 2972.72 (2807.32-3206.13) | 3570.79 (3199.19-3968.81) | 0.2 (0.14-0.24) | 0.66 (0.62-0.71) | 0.81 (0.73-0.9) | 0.38 (-0.02-0.78) |
| Middle SDI | 20-24 years | 241.26 (216.41-273.54) | 154.82 (134.68-178.4) | -0.36 (-0.38--0.35) | 0.25 (0.22-0.28) | 0.21 (0.18-0.24) | -1.05 (-1.78--0.31) |
| Middle SDI | 25-29 years | 485.51 (453.22-533.22) | 438.18 (379.11-496.27) | -0.1 (-0.16--0.07) | 0.52 (0.49-0.57) | 0.52 (0.45-0.59) | 0.2 (-0.67-1.08) |
| Middle SDI | 30-34 years | 889.04 (832.24-965.17) | 1145.24 (994.36-1287.87) | 0.29 (0.19-0.33) | 1.04 (0.98-1.13) | 1.07 (0.93-1.21) | 0.26 (-0.3-0.82) |
| Middle SDI | 35-39 years | 1244.43 (1168.32-1345.81) | 1769.08 (1606.87-1962.19) | 0.42 (0.38-0.46) | 1.55 (1.46-1.68) | 1.74 (1.58-1.93) | -0.12 (-0.42-0.19) |
| Middle SDI | 15-19 years | 40.48 (37.68-43.66) | 23.77 (22.38-25.37) | -0.41 (-0.41--0.42) | 0.06 (0.06-0.07) | 0.04 (0.04-0.04) | -1.87 (-2.07--1.68) |
| Middle SDI | 15-39 years | 1352.08 (1283.93-1404.9) | 1057.09 (1018.24-1102.8) | -0.22 (-0.21--0.22) | 0.39 (0.37-0.4) | 0.3 (0.29-0.31) | -1.11 (-1.24--0.97) |
| High-middle SDI | 20-24 years | 89.16 (83.34-94.12) | 66.29 (62.99-69.48) | -0.26 (-0.24--0.26) | 0.13 (0.12-0.14) | 0.1 (0.1-0.11) | -0.9 (-1.04--0.75) |
| High-middle SDI | 25-29 years | 204.73 (192.78-216.94) | 162.23 (154.87-169.98) | -0.21 (-0.2--0.22) | 0.28 (0.26-0.3) | 0.23 (0.22-0.24) | -0.6 (-0.72--0.48) |
| High-middle SDI | 30-34 years | 406.64 (382.13-428.82) | 320.91 (304.51-339.85) | -0.21 (-0.2--0.21) | 0.56 (0.53-0.6) | 0.41 (0.39-0.44) | -1.05 (-1.18--0.93) |
| High-middle SDI | 35-39 years | 611.07 (576.2-637.72) | 483.88 (463.57-509.54) | -0.21 (-0.2--0.2) | 0.9 (0.85-0.94) | 0.62 (0.59-0.65) | -1.5 (-1.63--1.37) |
| High-middle SDI | 15-19 years | 885.15 (716.32-1209.36) | 861.85 (701.17-1096.75) | -0.03 (-0.02--0.09) | 0.17 (0.14-0.23) | 0.14 (0.11-0.18) | -0.7 (-0.87--0.53) |
| High-middle SDI | 15-39 years | 13069.17 (11366.38-15762.89) | 16799.91 (14826.03-19749.17) | 0.29 (0.3-0.25) | 0.6 (0.52-0.72) | 0.56 (0.5-0.66) | -0.29 (-0.44--0.15) |
| High-middle SDI | 20-24 years | 1511.61 (1231.95-1927.34) | 1554.35 (1288.06-1965.28) | 0.03 (0.05-0.02) | 0.31 (0.25-0.39) | 0.26 (0.22-0.33) | -0.71 (-0.93--0.48) |
| High-middle SDI | 25-29 years | 2509.23 (2142.8-3204.68) | 3032.72 (2568.34-3712.93) | 0.21 (0.2-0.16) | 0.57 (0.48-0.72) | 0.52 (0.44-0.63) | -0.33 (-0.6--0.06) |
| High SDI | 30-34 years | 3512.45 (3058.86-4408.76) | 4690.77 (4072.68-5640.71) | 0.34 (0.33-0.28) | 0.91 (0.79-1.14) | 0.78 (0.67-0.93) | -0.47 (-0.67--0.28) |
| High SDI | 35-39 years | 4650.73 (4058.92-5751.66) | 6660.22 (5823.63-7968.7) | 0.43 (0.43-0.39) | 1.32 (1.15-1.63) | 1.19 (1.04-1.42) | -0.46 (-0.57--0.35) |
| High SDI | 15-19 years | 109.22 (78.12-171.91) | 185.25 (142.11-244.03) | 0.7 (0.82-0.42) | 0.22 (0.15-0.34) | 0.15 (0.11-0.2) | -1.2 (-1.46--0.94) |
| High SDI | 15-39 years | 1229.87 (848.44-1726.6) | 2394.37 (1766.5-3228.14) | 0.95 (1.08-0.87) | 0.67 (0.46-0.94) | 0.53 (0.39-0.72) | -0.8 (-0.88--0.72) |
| High SDI | 20-24 years | 156.23 (111.84-227.35) | 274.66 (210.51-395.57) | 0.76 (0.88-0.74) | 0.37 (0.26-0.53) | 0.26 (0.2-0.38) | -1.07 (-1.25--0.9) |
| High SDI | 25-29 years | 259 (173.57-371.44) | 503.5 (363.29-687.15) | 0.94 (1.09-0.85) | 0.72 (0.48-1.04) | 0.58 (0.42-0.8) | -0.92 (-1.05--0.79) |
| High SDI | 30-34 years | 287.01 (186.41-427.31) | 548.68 (388.11-775.6) | 0.91 (1.08-0.82) | 0.97 (0.63-1.44) | 0.76 (0.54-1.07) | -1.11 (-1.23--0.99) |
| High SDI | 35-39 years | 418.42 (272.5-626.52) | 882.27 (595.38-1262.03) | 1.11 (1.18-1.01) | 1.64 (1.07-2.46) | 1.42 (0.96-2.03) | -0.44 (-0.53--0.35) |
